# Triku: a feature selection method based on nearest neighbors for single-cell data

**DOI:** 10.1093/gigascience/giac017

**Published:** 2022-03-12

**Authors:** Alex M Ascensión, Olga Ibáñez-Solé, Iñaki Inza, Ander Izeta, Marcos J Araúzo-Bravo

**Affiliations:** Biodonostia Health Research Institute, Computational Biology and Systems Biomedicine Group, Paseo Dr. Begiristain, s/n, Donostia-San Sebastian, 20014, Spain; Biodonostia Health Research Institute, Tissue Engineering Group, Paseo Dr. Begiristain, s/n, Donostia-San Sebastian, 20014, Spain; Biodonostia Health Research Institute, Computational Biology and Systems Biomedicine Group, Paseo Dr. Begiristain, s/n, Donostia-San Sebastian, 20014, Spain; Biodonostia Health Research Institute, Tissue Engineering Group, Paseo Dr. Begiristain, s/n, Donostia-San Sebastian, 20014, Spain; Intelligent Systems Group, Computer Science Faculty, University of the Basque Country, Donostia-San Sebastian, 20018, Spain; Biodonostia Health Research Institute, Tissue Engineering Group, Paseo Dr. Begiristain, s/n, Donostia-San Sebastian, 20014, Spain; Biodonostia Health Research Institute, Computational Biology and Systems Biomedicine Group, Paseo Dr. Begiristain, s/n, Donostia-San Sebastian, 20014, Spain; Max Planck Institute for Molecular Biomedicine, Roentgenstr. 20, 48149 Muenster, German; IKERBASQUE, Basque Foundation for Science, Euskadi plaza 5, Bilbao, 48009, Spain; Department of Cell Biology and Histology, Faculty of Medicine and Nursing, University of Basque Country (UPV/EHU), 48940 Leioa, Spain

**Keywords:** sc-RNAseq, feature selection, machine learning, bioinformatics, Python

## Abstract

**Background:**

Feature selection is a relevant step in the analysis of single-cell RNA sequencing datasets. Most of the current feature selection methods are based on general univariate descriptors of the data such as the dispersion or the percentage of zeros. Despite the use of correction methods, the generality of these feature selection methods biases the genes selected towards highly expressed genes, instead of the genes defining the cell populations of the dataset.

**Results:**

Triku is a feature selection method that favors genes defining the main cell populations. It does so by selecting genes expressed by groups of cells that are close in the *k*-nearest neighbor graph. The expression of these genes is higher than the expected expression if the *k*-cells were chosen at random. Triku efficiently recovers cell populations present in artificial and biological benchmarking datasets, based on adjusted Rand index, normalized mutual information, supervised classification, and silhouette coefficient measurements. Additionally, gene sets selected by triku are more likely to be related to relevant Gene Ontology terms and contain fewer ribosomal and mitochondrial genes.

**Conclusion:**

Triku is developed in Python 3 and is available at https://github.com/alexmascension/triku.

## Introduction

Single-cell RNA sequencing (scRNA-seq) is a powerful technology to study the biological heterogeneity of tissues at the individual cell level, allowing the characterization of new cell populations and cell states—i.e., cell types responding to different environmental stimuli—previously undetected owing to their low frequency within the tissue and the lack of individual resolution of bulk methods [1,2].

Gene expression datasets are highly dimensional, as the expression of tens of thousands of genes is measured in any given experiment. A direct consequence of this is the curse of dimensionality, where the amount of data needed to fill the sampling space increases exponentially with the dimensions, resulting in a sparsity of the data [3]. Additionally, this sparsity is exacerbated by the low capture efficiency of messenger RNA (mRNA) in single-cell experiments, owing to the tiny amounts genetic material to be amplified, even though there are considerable recovery differences across methods [4]. This sparsity affects downstream methods such as cell type detection or differential gene expression [5].

A common task when working with multidimensional datasets is feature selection (FS). FS, alongside feature extraction (FE), responds to the need of obtaining a reduced dataset with a smaller dimensionality [6]. While FE methods such as principal component analysis (PCA) extract new features on the basis of combinations of the original features, FS methods aim to select a subset of the features that best explains the original dataset.

There are 3 main types of FS methods: filter, wrapper, and embedded methods [6].

- Univariate filter methods look at intrinsic properties of the data (e.g., variance, correlation), calculating a univariate score per feature, ranking them and removing the low-scoring ones. These techniques are usually fast and scalable and are independent of downstream methods. Common examples of univariate filter techniques are selection by variance, χ^2^, *t*-test, ANOVA, or information gain ratio.
- Wrapper methods embed the subsequent supervised algorithm within the feature search process. They perform a heuristic search in the space of feature subsets and score each subset of features with the scoring associated to the classification model. A common wrapper method type is genetic algorithms.
- Embedded methods include the search of the subset of features within the model construction. A common embedded method type is decision trees.

Current FS methods in scRNA-seq analysis are filter methods because common downstream analysis steps do not embed the FS within the pipeline [7]. FS methods represent a key step in processing pipelines of bioinformatic datasets [8] and provide several advantages [6]: they reduce model overfitting risk, improve clustering quality, and favor a deeper insight into the underlying processes that generated the data (features [genes] that contain random noise do not contribute to the biology of the dataset and are removed). Specifically, in scRNA-seq, removing non-informative features can improve results in downstream analyses such as differential gene expression.

Early methods for FS in scRNA-seq data were based on the idea that genes whose expression shows a greater dispersion across the dataset are the ones that best capture the biological structure of the dataset [9,10]. Conversely, genes that are evenly expressed across cells are unlikely to define cell types or cell functions in a heterogeneous dataset. The most straightforward way of selecting genes that are not evenly expressed is to look at a measure of dispersion of the counts of each gene and to select those genes that have a dispersion exceeding a threshold.

However, the correlation between mean expression and dispersion introduces a bias whereby genes with higher expression are more likely to be selected by FS methods. However, biological gene markers that define minor cell types are usually expressed in a medium to small subset of cells. Therefore, new FS methods based on dispersion are designed to correct for this dispersion/expression correlation to select genes with a broader expression spectrum.

Brennecke et al. [9] developed a FS method that introduces a correction over the dispersion that accounts for differences in the mean expression of genes. It does so by setting a threshold to the correlation between the average gene expression and its coefficient of variation across cells. Newer FS methods have arisen after different corrections, such as the one originally described by Stuart et al. [11] implemented in Seurat, later adapted to scanpy [12], a later evolution of the method developed in sctransform [13], or the one implemented in scry [14].

Early studies observed that the read distribution of most of the single-cell studies could be fitted to a negative binomial (NB) [15]. More specifically, read counts produced a zero-inflated bimodal distribution, whereas unique molecular identifier (UMI) counts produced an NB distribution [16]. These results were later replicated by Svensson, stating that the proportion of zeros in droplet-based scRNA-seq data, originally assumed to be dropouts, was tightly related to the mean expression of genes, following an NB curve [17]. Genes with an expected lower percentage of zeros tend to have an even expression across the entire set of cells. Conversely, genes with a higher than expected percentage of zeros might possess biological relevance because they are expressed in fewer cells than expected, and these cells might be associated to a specific cell type or state.

This finding opened the path for new FS methods that would rely on genes that showed a greater than expected proportion of zeros, according to their mean expression. These methods are based on a null distribution of some property of the dataset, and genes whose behavior differs from the expected are selected. The FS method nbumi, an NB method based on m3drop [18], works under this premise. nbumi fits the NB zero-count probability distribution to the dataset and selects genes of interest, calculating *P*-values of observed dropout rates. m3drop works similarly by fitting a Michaelis-Menten model instead of the NB from nbumi.

**Figure 1:**
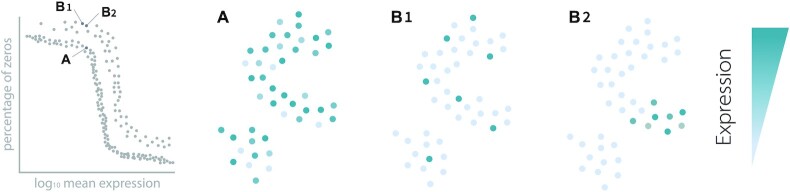
: Distribution of gene expression in 3 scenarios. There are 3 main patterns of expression for any particular gene in a single-cell dataset: (A) The gene is expressed evenly across cells in the dataset, which probably means it does not define any particular cell type. (B) A gene shows an unexpected distribution of zeros, because it is only expressed by a subset of cells. Within case B, there are 2 possible patterns. (B1) The gene is highly expressed by a subset of transcriptomically different cells (i.e., cells that are not collocalized in the dimensionally reduced map) and (B2) the gene is highly expressed by cells that share an overall transcriptomic profile. Triku preferentially selects the genes shown in the B2 pattern. When looking at the proportion of zeros, genes in cases B1 and B2 show an increased proportion of zeros with respect to A, but they are indistinguishable from each other by that metric.

In summary, existing FS methods assume that an unexpected distribution of counts for a particular gene in a dataset is explained by cells belonging to different cell types. However, we consider that there are 3 main patterns of expression according to the distribution of zeros of a particular gene and overall transcriptional similarity (expression of all genes), as explained in detail in Fig. 1: (A) a gene evenly expressed across cells, or a gene expressed by a subset of cells, which can be (B1) transcriptomically separate or (B2) transcriptomically similar. These patterns can be seen, e.g., in genes *Dhx30, Cog3*, and *Lyz2* in Supplementary Fig. S8. Thus, in some cases a particular gene shows an unexpected distribution of counts because a subset of cells are expressing it but those cells might not be transcriptomically similar.

**Figure 2:**
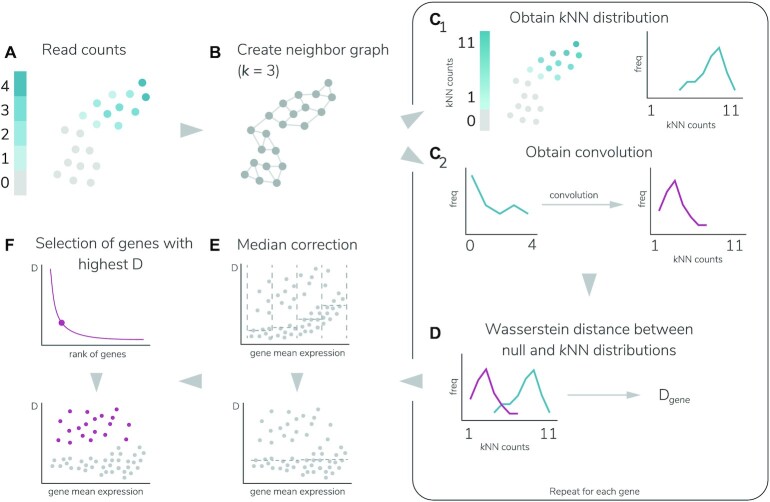
: Graphical abstract of triku workflow. (A) DR representation of the gene expression from the count matrix from a dataset, where each dot represents a cell. (B) KNN graph representation with 3 neighbors. For each cell the *k* transcriptomically most similar cells are selected (3 in this example). (C1) Considering the graph in (B) for each cell with positive expression, the expression of its *k* neighbors is summed to yield the KNN distribution in blue. (C2) With the distribution of reads (blue line), the null distribution is estimated by sampling *k* random cells. (D) The null and KNN distributions of each gene are compared using the Wasserstein distance. (E) For each gene, its distance is plotted against the log mean expression, and divided into *w* windows (4 in this example). For each window, the median of the distances is calculated and subtracted from the distances in that window. (F) All corrected distances are ranked and the cut-off point is selected.

Here we present triku, an FS method that selects genes that show an unexpected distribution of zero counts and whose expression is localized in cells that are transcriptomically similar. Figure 2 summarizes the feature selection process. Triku identifies genes that are locally overexpressed in groups of neighboring cells by inferring the distribution of counts in the vicinity of a cell and computing the expected distribution of counts. Then, the Wasserstein distance between the observed and the expected distributions is computed and genes are ranked according to that distance. Higher distances imply that the gene is locally expressed in a subset of transcriptomically similar cells. Finally, a subset of relevant features is selected using a cut-off value for the distance. Triku outperforms other feature selection methods on benchmarking and artificial datasets, using unbiased evaluation metrics such as normalized mutual information (NMI) or Silhouette. Of note, features selected by triku are more biologically meaningful, as compared to other methods.

## Results

The objective of FS methods is to select the features that are the most relevant in order to understand and explain the structure of the dataset. In the context of single-cell data, this means finding the subset of genes that, when given as input to a clustering method, will yield a clustering solution where each cluster can be annotated as a putative cell type.

Initially, we generated artificial datasets with the splatter package [19], so that cells belonging to the same cluster have a similar gene expression. All datasets contained the same number of genes, cells, and populations, but differed in the de.prob parameter value. This parameter was set so that higher values indicate a higher probability of genes being differentially expressed, resulting in more resolved populations. A combination of 8 de.prob values, from 0.0065 to 0.3, were used (see Methods). In addition, we tested triku on 2 biological benchmarking datasets by Ding et al. [20] and Mereu et al. [21] that have been expert-labeled using a semi-supervised procedure. Both benchmarking datasets are composed of individual subsets of data with different library preparation methods (e.g., 10X, SMART-seq2) in human peripheral blood mononuclear cells (PBMCs) (Mereu and Ding) and mouse colon (Mereu) and cortex (Ding) cells.

We have evaluated the relevance of the features selected by triku by comparing them to those selected using other feature selection methods, similar to [8,9,14,18]. The relevance of the features was first measured using metrics associated to the efficacy of clustering, and then using metrics to evaluate the quality of the genes selected.

We made 6 types of comparisons between the subsets of genes selected by each feature selection method: (i) the ability to recover basic dataset structure (main cell types) in artificial and biological datasets, (ii) the ability to obtain transcriptomically distinct cell clusters, (iii) the overlap of features between different FS methods, (iv) the localized pattern of expression of the features selected, (v) the ability to avoid the overrepresentation of mitochondrial and ribosomal genes, (vi) the biological relevance of the genes by studying the composition and quality of the gene ontology (GO) terms obtained, and (vii) the resolution of cell types and subtypes on the UMAP.

### triku efficiently recovers cell populations present in sc-RNAseq datasets

The first set of metrics evaluates the ability to recover the original cell types based on the NMI index, and the cluster separation and cohesion using the Silhouette coefficient.

#### NMI

NMI measures the correspondence between a labeling considered as the ground truth and the clustering solution that we obtained using the genes selected by triku and other FS methods.

**Figure 3:**
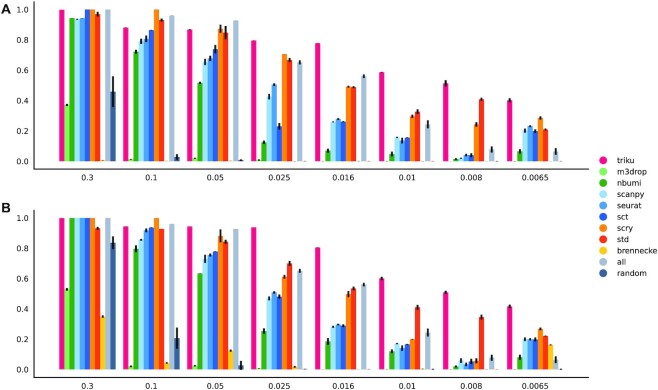
: Comparison of NMI for FS methods on artificial datasets. Bar plots of the NMI for all FS methods with different artificial datasets, using the top 250 (A) and 500 (B) features of each FS method. The probability of the selected genes being differentially expressed between clusters (de.prob) is shown in the x-axis. Higher NMI values mean better recovery of the cell populations. Note that in category "all," all features are selected, not the top 250 or 500; therefore their NMI values are the same in both graphs.

First, we evaluated how well the clustering using the genes selected by the FS methods was able to recover the same populations that were defined when generating the artificial datasets. Figure 3 shows that triku is among the 3 best feature selection methods for a wide range of de.prob values. For low values of de.prob (<0.05), where the selection of genes that lead to a correct recovery of cell populations is more challenging, triku notably outperforms the rest of the FS methods. NMI values obtained with triku are 0.1–0.2 higher than the second and third best FS methods. In addition, the results obtained when using the first 250 selected genes were comparable to those obtained when selecting 500 genes, showing that this efficiency is independent of the amount of genes chosen within a sensible range. These results are replicated using the adjusted Rand index (ARI), used by others [14,22,23], in Supplementary Fig. S1.

**Figure 4:**
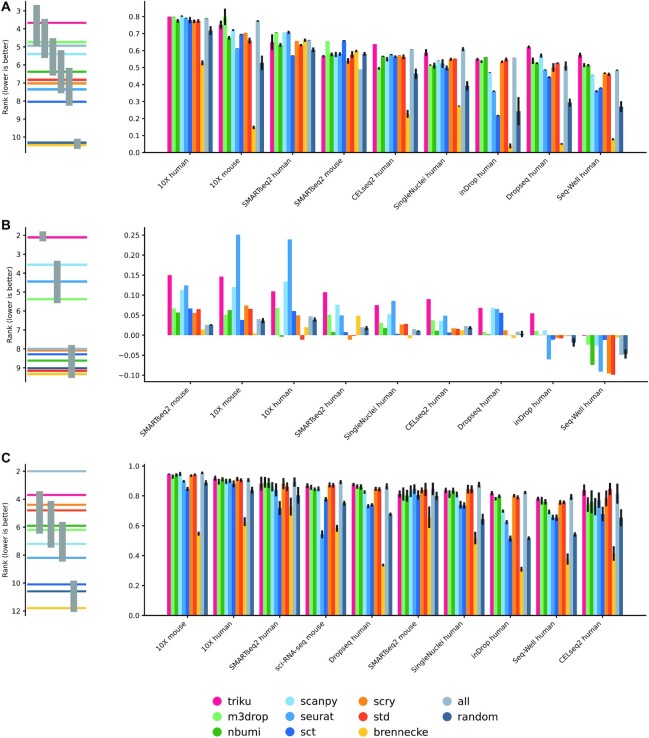
: NMI, silhouette of annotated cell types and decision tree metrics in Ding datasets. Bar plots of the 3 represented metrics. Each bar plot represents the mean over 5 runs, and the error bar is the standard deviation. (A) NMI between clustering solutions and annotated cell types. (B) Silhouette coefficient of annotated cell types. (C) Decision tree classifier accuracy using a 10-fold cross validation of annotated cell types. The plot on the left is a critical difference diagram, where each horizontal bar represents the mean rank for all datasets. If 2 or more bars are linked by a gray vertical bar, the mean ranks for those FS methods are not significantly different (Quade test, α = 0.05).

**Figure 5:**
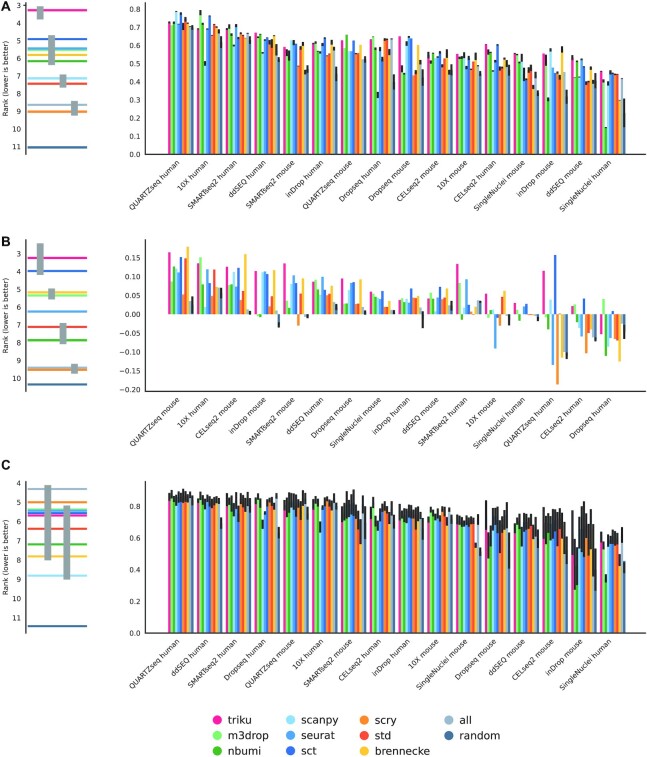
: NMI, silhouette of annotated cell types and decision tree metrics in Mereu datasets. Bar plots of the 3 represented metrics. Each bar plot represents the mean over 5 runs, and the error bar is the standard deviation. (A) NMI between clustering solutions and annotated cell types. (B) Silhouette coefficient of annotated cell types. (C) Decision tree classifier accuracy using a 10-fold cross validation of annotated cell types. The plot on the left is a critical difference diagram, where each horizontal bar represents the mean rank for all datasets. If 2 or more bars are linked by a gray vertical bar, the mean ranks for those FS methods are not significantly different (Quade test, α = 0.05).

We also studied how well the selected genes led to a clustering solution that was similar to the manually assigned cell labels in the biological benchmarking datasets, as shown in Figs 4A and 5A. For each dataset, the variability between NMI scores was quite low, meaning that features selected with the different methods yielded clustering solutions that were quite similar to the manually labeled cell types, although there are some exceptions to this rule—e.g., brennecke in Ding datasets, or scanpy in some Mereu datasets, which showed notably reduced NMI values. In some datasets, e.g., 10X human, QUARTZseq human, and SMARTseq2 human from Mereu’s benchmarking set, features selected by FS methods did not lead to increased NMI values as compared with randomly selected genes.

Despite the differences in NMI between methods being small for each particular dataset, post hoc analysis revealed that triku is the best ranked method across all datasets. To do the post hoc analysis, we ranked for each dataset the NMI of each FS method. Figures 4A and 5A (left) show the mean rank of each FS method across datasets. Triku is the best-ranked FS method in both Mereu and Ding benchmarking datasets, with a mean rank of 3.2 and 3.7, respectively. m3drop is the second and third best-ranked FS method for Mereu and Ding datasets, respectively. For Mereu datasets this difference is statistically significant, whereas for Ding datasets, the difference is not statistically significant compared to the second best method, m3drop, but it is significant compared to the rest (Quade test, *P* < 0.05).

Supplementary Figs S2A and S3A show similar results using ARI instead of NMI, where triku is statistically significantly the best FS method for Mereu datasets, and the best together with m3drop for Ding datasets.

#### Silhouette coefficient

Another important aspect of the genes selected by FS methods in scRNA-seq data analysis is their ability to cluster data into well-separated groups that are transcriptomically similar. We used the Silhouette coefficient to measure the compactness and separation-degree of cell communities obtained with a clustering method. When the same clustering algorithm is used on a dataset but using 2 different FS methods, the differences in the resulting Silhouette coefficients can be entirely attributed to the features selected by those methods. We assume that FS methods that increase the separation between clusters and the compactness within clusters are better at recovering the cell types present in the dataset.

Figures 4B and 5B show the Silhouette coefficients obtained with the different FS methods. For the Mereu and Ding datasets, we observed that triku was the best-ranked method (mean rank of 3.1 and 2.1), and the second best-ranked methods were m3drop and scanpy, with a mean rank of 3.9 and 3.5, respectively. In Ding datasets, the difference between triku and the second-ranked method was statistically significant (Quade test, *P* < 0.05).

We performed an additional analysis using the labels obtained with Leiden clustering instead of the manually curated cell types (Supplementary Figs S2B and S3B). Again, triku outperformed the rest of the FS methods, showing a statistically significant best mean rank, for both benchmark datasets.

#### Supervised cell type classifiers

As an additional measure of FS method accuracy, we trained 2 classifiers, decision tree and KNN, using a 10-fold cross-validation, as shown in [24]. The results for decision tree classifier in Ding and Mereu are shown in Figs 4C and 5C, and the results for KNN classifier are shown in Supplementary Figs S2C and S3C.

In general, classifier efficiency shows high variance, even more for Mereu datasets, and therefore the results are not completely conclusive. From the critical difference diagrams, we can state that triku is in range with most of the FS methods for decision tree and KNN classifier. In general, we see a decrease of efficiency in Ding datasets for seurat, sct, and sometimes m3drop, which act similar to a random choice of features, whereas efficiency of brennecke is worse than a random choice of features.

For triku, nbumi, scry, std, and selecting all features, their efficiencies are similar.

Despite the 10-fold cross validation accuracy of decision trees ranking the highest when trained on the whole set of features, the actual difference in performance between triku and using all features is negligible. Overall, FS does not have a strong effect on classifier accuracy.

### Genes selected by different FS methods show limited overlap

Next, we studied the characteristics of the genes selected by triku and compared them to the genes selected by other methods.

**Figure 6:**
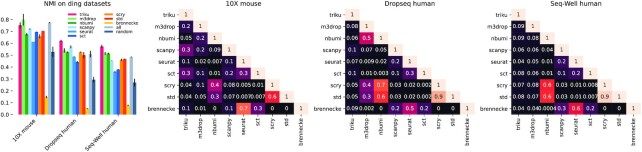
: Heat maps of overlap of features between pairs of methods. Bar plot on the left represents the NMI values for the 3 selected datasets on the overlap heat maps on the right. For each pair of methods, the value represents the proportion of features that are shared between the 2 methods. The number of genes selected in each method is the automatic cut-off by triku.

Initially, we studied the level of consistency between the results obtained using different FS methods by studying their degree of overlap, as shown in Fig. 6. To compare between equally sized gene lists, we ranked the genes based on *P*-values or scoring value from each FS method and set the number of genes selected by triku as a cut-off to select the first genes. Although the genes selected by the different methods yielded clustering solutions that are highly consistent, as shown in the previous section, we did not see any clear gene overlap pattern between pairs of FS methods. Actually, there is no correlation between the degree of overlap between the genes selected by the different methods and the clustering solutions that are obtained when using those genes as input.

For instance, we found an overlap of 7% between the genes selected by scanpy and std for the 10x mouse dataset, yet the NMI between the clustering solutions obtained with each of them and the expert-labeled cell types was 0.7. On the other hand, the overlap between seurat and brennecke is one of the highest across datasets (ranging from 50 to 70%), yet the differences between their corresponding NMI scores are 0.45.

### triku selects genes that are biologically relevant

On the basis of these results, we studied the biological relevance of the genes selected by different FS methods in 3 alternative ways.

Genes whose expression, or lack thereof, is limited to a single population are more likely to be cell-type specific and thus might be better candidates as positive or negative cell population markers. Therefore, we studied which are the best FS methods to select genes showing a localized expression pattern.

Mitochondrial and ribosomal genes are usually highly expressed, and many FS methods tend to overselect them despite them not being particularly relevant in most single-cell studies. In fact, they are commonly excluded from downstream analysis [22,25,26]. Assuming that in these benchmarking datasets ribosomal and mitochondrial genes are not as relevant to the biology of the dataset, we measured the percentage of these genes in the subset of genes selected by triku and compared it to other FS methods.

Last, we analyzed the biological pertinence of the selected genes by performing gene ontology enrichment analysis (GOEA) on a dataset of immune cell populations whose underlying biologic characteristics are well understood, as a robust indicator of FS quality; and analyzed UMAP visual quality of different samples to assert that cell subtypes were independently represented in the UMAP.

#### Selection of locally expressed genes

We first studied the expression pattern of genes selected by triku and other methods, as shown in Supplementary Fig. S4. We observed that of the 9 populations of the artificial dataset, when a gene is selected by triku—exclusively or together with other FS methods—1 of the populations had a markedly higher or lower expression compared to the rest. On the other hand, when a gene is selected by other FS methods and not by triku, we do not observe any population-specific expression pattern. For instance, genes exclusively selected by scanpy had a wide expression variation across clusters, but they were not exclusive of 1 or 2 clusters. Features selected by std and scry showed some variation, but it was overshadowed by the high expression of the gene and therefore not relevant under the previous premise.

To evaluate the cluster expression of selected genes in benchmarking datasets, for each gene we scaled its expression to the 0–1 range and sorted the clusters so that the first one had the greatest expression. Supplementary Fig. S5 shows the expression patterns for several benchmarking datasets. We see that, in most datasets, triku showed more biased expression patterns; i.e., genes selected by triku were expressed, on average, on fewer clusters than the genes selected by other FS methods. The following best methods were scanpy, seurat, sct, and brennecke, with similar or slightly less biased expression patterns as compared to triku. With these methods, up to 80% of the expression of the gene was usually restricted to the 2–3 clusters that most expressed it.

m3drop and nbumi performed similarly, and showed an expression distribution across clusters similar to a random selection of genes, which was slightly biased towards 3–5 clusters accumulating up to 80% of the expression of the gene. Last, std and scry methods were the least biased, and showed almost a linear decrease of expression percentage across clusters, with 4–6 clusters accumulating up to 80% of the expression of the gene.

#### Avoidance of mitochondrial and ribosomal genes

Table 1 shows the percentage of genes that code for ribosomal and mitochondrial proteins within the genes selected by different FS methods in the 2 sets of benchmarking datasets. We observed that std and scry, followed by m3drop, were the only methods that tended to overselect mitochondrial and ribosomal genes. Among the rest of the methods, triku showed percentages that were comparable to the rest of the methods, and slightly lower for the Ding datasets.

**Table 1.** Percentage of ribosomal protein (RBP) and mitochondrial (MT) genes appearing within the selected genes by each FS method

Method	Mereu		Ding	
	% RBP	% MT	% RBP	% MT
**triku**	1.92	0.08	0.12	0.01
**m3drop**	3.83	0.43	0.69	0.11
**nbumi**	1.87	0.16	0.44	0.09
**scanpy**	1.56	0.09	0.27	0.04
**seurat**	1.96	0.20	0.25	0.01
**sct**	1.34	0.18	0.04	0.03
**scry**	4.20	0.61	1.31	0.27
**std**	5.04	0.58	2.00	0.33
**brennecke**	1.01	0.09	0.03	0.01

#### Selection of genes based on gene ontologies

We assessed the quality of the GO output by studying its term composition. We selected 2 PBMC datasets from the Ding datasets. We used PBMC datasets for this analysis because their cell-to-cell variability has been extensively studied using single-cell technologies such as fluorescence-activated cell sorting (FACS) and scRNA-seq [27–31]. Using these datasets, we measured the proportion of GO terms obtained in the output that were tightly related to the biological system under study.

**Figure 7:**
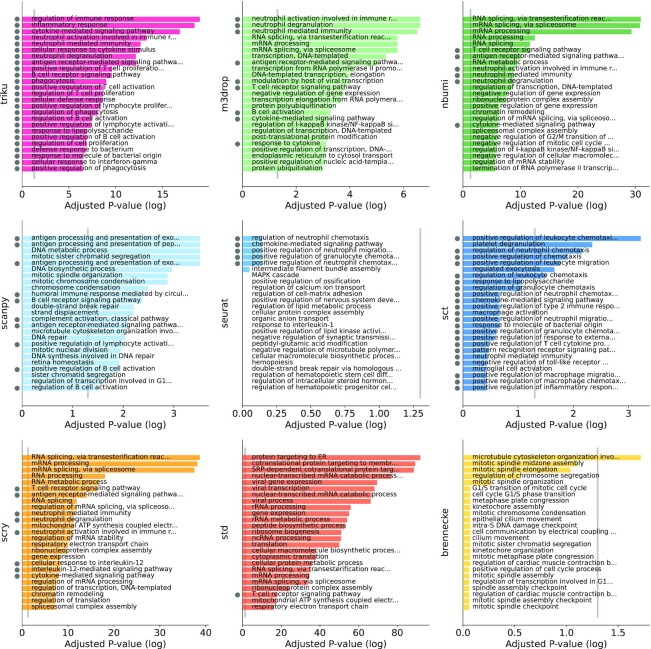
: Bar plot of *P*-values of GOEA. Each bin represents the number of features selected for each method, in Ding et al. [20] human Dropseq dataset. The y-value is the −log10 adjusted *P*-value for the best 25 ontologies. On the bottom, the bar plot shows the names of the ontology terms for the case with the best 1,000 features. In immune datasets, gray dots at the left of each term represent that that term is directly related to an immune process. Non-dotted terms refer to more general processes that may or may not be related to immune processes.

Figure 7 and Supplementary Fig. S6 show the first 25 GO terms obtained with the genes selected by each FS method on the 2 PBMC datasets (10X human and Dropseq human) where the terms tightly related to immune processes—chosen by 3 independent assessors—have been highlighted. We observed that triku was the FS method that yielded the most terms directly related to immune processes, with 25/25 + 15/25 = 40/50 related terms in the Ding Dropseq and 10X datasets, respectively. Examples of terms that we considered to be tightly related to immune processes included "B cell receptor signalling pathway," "neutrophil degranulation," and "regulation of T cell proliferation." The next methods were scanpy and m3drop, whose performances were comparable to or better than that of triku for the 10X dataset (21/25 and 15/25) but less robust for the Dropseq dataset (10/25 and 9/25 related terms), summing up to a total of 33/50 and 24/50. The rest of the FS methods mainly selected genes that were related to general cell functions such as RNA processing, protein processing, and cell-cycle regulation.

#### Cell subtype distribution on UMAP

UMAP and clustering are common steps within single-cell pipelines. To assess the quality of FS on the UMAP representation, we analyzed whether different cell types appeared as different entities in the UMAPs. In other words, if 2 cell types that have different transcriptional profiles appear mixed within the UMAP, it is possible that some of the features from the transcriptional profile of the cell types are not selected as relevant.

**Figure 8:**
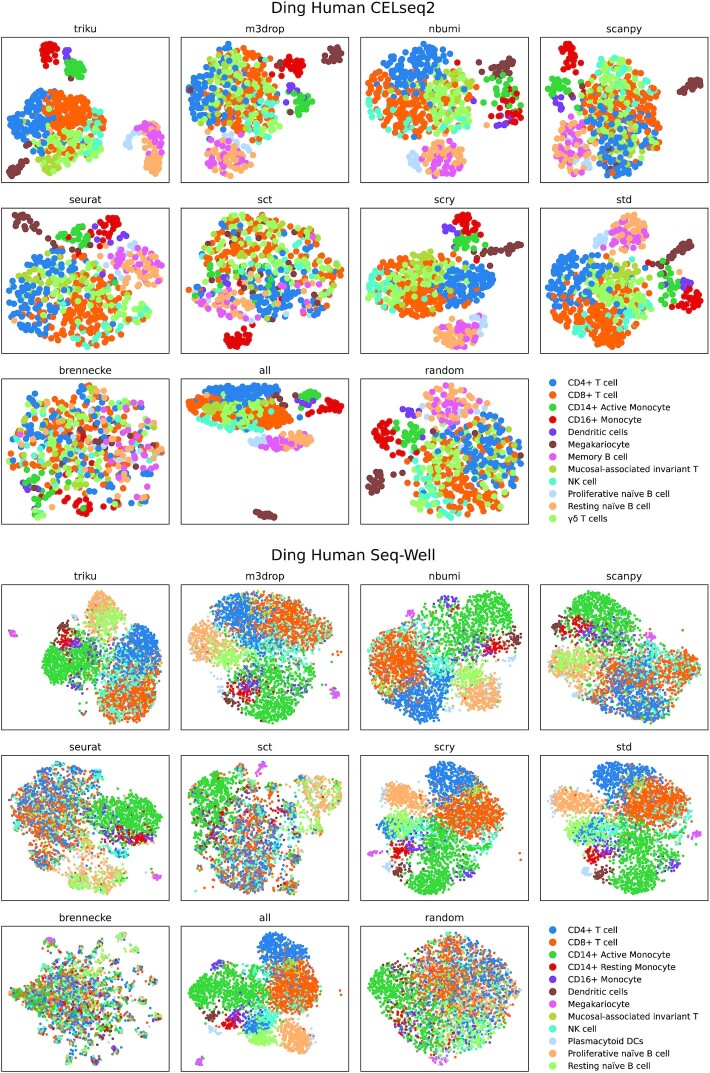
: UMAP plots based on features selected by different methods. The UMAPs show cell subtypes from 2 Ding datasets in human PBMCs. For each FS method, the features were set as highly variable, and UMAP was run based on the neighborhood graph constructed from the selection of features using min_score=0.1. To assign the cell types, Leiden was run to produce a high number of clusters, and a cell-matching algorithm was used based on a set of markers to assign clusters to cell types. Each cell subtype should ideally appear in a separate group of cells and not be mixed with other cell subtypes.

We analyzed 2 Ding human PBMC datasets, CELseq2 and Seq-Well, as shown in Fig. 8. In general, we observe a high mixture of cell types within brennecke and random FS throughout datasets, where major cell types were mixed and, therefore, would be highly uninformative for cell type characterization.

From the CELseq2 dataset we observe that the mucosal-associated invariant T-cell population is more diffuse in m3drop, seurat, sctransform, and all; and γδ T cells showed a higher mixture degree in scanpy, seurat, and sctransform. Additionally, the dendritic cell population, which appeared near CD14^+^ monocytes, appears less separated in std and all and is mixed with CD16^+^ monocytes in seurat. Finally, memory and resting naive B cells appear less separated in m3drop, nbumi, scanpy, seurat, and sct.

Regarding the Seq-Well dataset we observe that mucosal-associated invariant T cells are less defined in triku, scanpy, m3drop, seurat, and sctransform; and seurat and sctransform show a high degree of mixture of major T-cell populations (CD4 and CD8). Additionally, resting naive B cells are mixed with proliferative naive B cells in scanpy, seurat, and sct; and with CD4^+^ T cells in scry and std.

Therefore from these results we conclude that using triku as FS method produces well-defined cell type populations and subpopulations. Interestingly, using no FS method also yields good visual results, even better than other FS methods, probably because PCA takes into consideration all the information from the genes, and the PCA projection automatically excludes nonrepresentative features. Nevertheless, this effect should be addressed with other datasets and other steps in the analysis pipeline.

## Discussion

FS methods are a key step in any scRNA-seq sequencing analysis pipeline because they help us obtain a dimensionally reduced version of the dataset that captures the most relevant information and eases the interpretation and understanding of its underlying biology. However, every FS method relies on a set of assumptions regarding what characteristics make a gene relevant. FS methods that sort genes according to their dispersion assume that gene expression variability is indicative of its biological relevance. FS methods like nbumi and m3drop assume that genes showing a proportion of zero-counts that is greater than expected (according to a null distribution) are more likely to be informative. Triku assumes that genes that have a localized expression in a subset of cells that share an overall transcriptomic similarity are more likely to define cell types. A general trend in FS method design has been to refine the requirements that a gene must meet in order for it to be selected, from the more general dispersion-based to more sophisticated formulations. It is noteworthy that the requirements in triku are consistent with the previous dispersion-based and zero-count–based formulations but involve a new aspect that we consider essential for an accurate gene selection: a localized expression in neighboring cells. Another important advantage of triku over FS methods that consider the zero-count distribution is that, unlike m3drop and nbumi, triku does not assume gene counts to follow any particular distribution, because it estimates the null distribution from the dataset, thus extending the range of single-cell technologies that it can use beyond droplet-based technologies.

We verified the locality of the genes selected by triku in different artificial and real scRNA-seq datasests and concluded that, on average, the expression of triku-selected genes is restricted to fewer, well-defined clusters. In addition, the clusters obtained when using triku-selected genes as input for unsupervised clustering in both artificially generated and biological datasets have a better resolved pattern structure, as shown by their increased Silhouette coefficients. In the case of artificial datasets, where the degree of mixture between clusters can be predefined, triku proved to be able to recover the originally defined cell populations. In fact, we found that the higher the degree of mixture between clusters, the more obvious the advantage of triku over the rest of the FS methods tested.

In general, a single metric is not sufficient to properly evaluate a novel computational method, but rather, all the results have to be considered as a whole to provide a general view of how the different FS methods work. In the present work, we have used a number of metrics (NMI, ARI, Silhouette, cross-validation accuracy of 2 supervised classifiers—decision tree and KNN—and degree of separation of distinct cell populations on UMAP plots). Although individual results may be more or less conclusive, we have a strong view that triku works among the best for that wide range of metrics.

An important difficulty in the interpretation of single-cell data is that we must consider that cell-to-cell variability has both technical and biological components. That is, it is difficult to know whether a set of genes is differentially expressed between cell clusters owing to technical reasons (differences in the efficiency of mRNA capture, amplification, and sequencing) or whether it constitutes a biological signal. Moreover, there is a wide range of sources of biological variability within a dataset, some of which might not be of interest depending on the experimental context. For instance, fluctuations in genes that regulate the cell cycle constitute a source of biological variability that is often disregarded. This has been extensively studied and addressed in a number of ways: normalization, regression of unwanted sources of variation, and so forth [13, 32–34].

The expression of genes whose variability is associated with technical reasons tends to have a high dispersion, but their expression is usually not restricted to a few clusters. A good example of these genes is the ribosomal and mitochondrial genes, which are expressed across all cell types at different levels. Our results show that these genes are in fact selected by the majority of compared FS methods due to their high expression and cell-to-cell variability but are less likely to be selected by triku because they do not usually meet the locality requirement. Additionally, when performing GOEA, we observed that the list of genes obtained with triku were more enriched for terms that are specifically related to a biological process of the system under study.

In our work, we have observed that the genes selected by different FS methods might show little overlap between them. This phenomenon has been described elsewhere [35]. In fact, gene covariation and redundancy is a well-characterized effect that has been observed in omics studies. The effect of redundancy arises from the fact that different cell types must have a common large set of pathways to be active. The difference between cell type and cell state is that 2 cell types might have large sets of pathways that are different between each other, and 2 cell states will only differ in a few pathways. Because pathways are composed of many genes, only choosing a reduced set of genes from a set of pathways from cell type A and B might be enough to differentiate them, and we might not need to select all genes from all pathways. This “paradigm” explains several effects. Qiu described that scRNA-seq datasets could preserve basic structure after gene expression binarization [36] or by conducting very shallow sequencing experiments [5]. This can be explained by the fact that only a few genes are necessary to describe the main cell populations in a single-cell dataset, and the presence/absence of a certain marker is often more informative than its expression level. This is related to the notion that despite the high dimensionality of omics studies, most biological systems can be explained in a reduced number of dimensions. Moreover, some authors have claimed this low dimensionality to be a natural and fundamental property of gene expression data [5]. This highlights the importance of designing accurate FS methods that extract the fundamental information from single-cell datasets.

Triku Python package is available at Github [37] and can be downloaded using PyPI. Triku has been designed to be compatible with scanpy syntax, so that scanpy users can easily include triku into their pipelines. Notebooks developed for figure production and additional results are located in GitHub [38] and in Zenodo [39].

## Methods

The triku workflow, parameter robustness, and run times are further described in Supplementary Methods.

### Artificial and benchmarking datasets

To perform the evaluation of the FS methods we used a set of artificial and biological benchmarking datasets. Artificial datasets were constructed using the splatter R package (v 1.10.1). Each dataset contains 10,000 cells and 15,000 genes and consists of 9 populations with abundances in the dataset of {25%, 20%, 15%, 10%, 10%, 7%, 5.5%, 4%, 3.5%} of the cells. Each dataset contains a parameter, de.prob, that controls the probability that a gene is differentially expressed. Lower de.prob values (<0.05) imply that different populations have fewer differentially expressed genes between them and, therefore, are more difficult to differentiate. Selected values of de.prob are {0.0065, 0.008, 0.01, 0.016, 0.025, 0.05, 0.1, 0.3}. Populations in datasets with de.prob values >0.05 are completely separated in the low-dimensionality representation with UMAP, even without feature selection (Supplementary Fig. S7).

Regarding biological datasets, 2 benchmarking datasets have been recently published by Mereu et al. [21] and Ding et al. [20]. The aim of these 2 works is to analyze the diversity of library preparation methods, e.g., 10X, SMART-seq2, CEL-seq2, single nucleus or inDrop. Mereu et al. use mouse colon cells and human PBMCs to perform the benchmarking, whereas Ding et al. use mouse cortex and human PBMCs. There are a total of 14 datasets in Mereu et al. and 9 in Ding et al. An additional characteristic of these datasets is that they have been manually annotated, and this annotation is useful as a semi ground truth. Ding dataset files were downloaded from Single Cell Portal (accession Nos. SCP424 and SCP425), and cell type metadata are located within the downloaded files. Mereu datasets were downloaded from GEO database (accession GSE133549), and cell type metadata were provided by the authors after personal request.

### FS methods

Triku is compared to the following FS methods:

- m3drop [18] (distribution-based): fits a Michaelis-Menten equation to the percentage of zeros versus μ, and selects features with higher percentages of zeros than expected. The features are selected with the M3DropFeatureSelection function from M3Drop R package.
- nbumi (distribution-based): acts in the same manner as m3drop but fitting an NB equation instead of a Michaelis-Menten equation. The features are selected with the NBumiFeatureSelectionCombinedDrop function.
- scanpy [12] (dispersion-based): selects features on the basis of a *z*-scored deviation, adapted from Seurat’s method. The features are selected with the sc.pp.highly_variable_genes function from scanpy (v 1.6.0).
- seurat [40] (dispersion-based): uses the FindVariableFeatures function, which fits a line to the relationship of log(variance) and log(mean) using local polynomial regression (loess). It then standardizes the feature values using the observed mean and expected variance.
- sctransform (sct) [13] (dispersion-based): Seurat implementation of SCTransform function applies NormalizeData, ScaleData, and FindVariableFeatures with vst mode (default in Seurat).
- scry [14] (dispersion-based): computes a deviance statistic for counts based on a multinomial model that assumes that each feature has a constant rate. The features are selected with the devianceFeatureSelection function from scry R package (v 0.99.0).
- Standard deviation (std) (dispersion-based): is computed directly using Numpy (v 1.18.3).
- brennecke [9] (dispersion-based): fits a curve based on the square of the coefficient of variation (CV^2^) versus the mean expression (μ) of each gene and selects the features with higher CV^2^ and μ. The features are selected with the BrenneckeGetVariableGenes function from M3Drop R package (v 1.12.0).

#### FS and dataset preprocessing

To make the comparison between FS methods, each feature is ranked on the basis of the score provided by each FS method. Calculating the ranking instead of just selecting the features allow us to select different numbers of features when needed. By default, the number of features is the one automatically selected by triku. Additionally, in some contexts, analyses are performed with all features or with a random selection of features.

After the ranking of genes is computed, dataset processing is performed equally for all methods, in artificial and benchmarking datasets. Datasets are first log transformed (if required by the method), and PCA with 30 components is calculated. Then, the *k*-nearest neighbors (KNN) matrix is computed setting *k* as (*n*_cells_)^1/2^. UMAP (v 0.3.10) is then applied to reduce the dimensionality for plotting. If community detection is required, Leiden (v 0.7.0) is applied, selecting the resolution that matches the number of cell types manually annotated in the dataset. This procedure is repeated with 10 different seeds. This conditions the output of triku, random FS, PCA projection, neighbor graph, Leiden community detection, and UMAP.

#### ARI and NMI calculation in artificial and benchmarking datasets

To compare the Leiden community detection results with the ground-truth labels from artificial and biological datasets, we used the ARI and the NMI scores [41].

The ARI is a revision of the RI, with correction of the expected RI:

\begin{eqnarray*}
\mathrm{ARI} = \frac{\mathrm{RI} - \mathrm{RI}_{\mathrm{Expected}}}{\mathrm{RI_{\mathrm{max}}} - \mathrm{RI}_{\mathrm{Expected}}}
\end{eqnarray*}

If *T* and *L* are the labels of the cell types (true populations) and Leiden communities, respectively, the NMI between *T* and *L* is

\begin{eqnarray*}
NMI(T, L) = \frac{2I(T;L)}{H(T) + H(L)}
\end{eqnarray*}

where *H*(*X*) is the entropy of the labels and *I*(*T; L*) is the mutual information between the 2 sets of labels. This value is further described in [42]. We used the scikit-learn (v 0.23.1) implementation of NMI, sklearn.metrics.adjusted_mutual_info_score.

One of the advantages of NMI against other mutual information methods is that it performs better with label sets with class imbalance, which are common in single-cell datasets, where there are differences in the abundance of cell types.

On artificial datasets, Leiden was applied using the first 250 and 500 selected features, and the resulting community labels were compared with the population labels from the dataset. On benchmarking datasets, Leiden was applied with the manually curated cell types.

#### Silhouette coefficient in benchmarking datasets

To assess the clustering performance of the communities obtained with benchmarking datasets we used the Silhouette coefficient. The Silhouette coefficient compares the distances of the cells within each cluster (intracluster) and between clusters (intercluster) within a measurable space. The distance between 2 cells is the cosine distance between their gene expression vectors, considering only the genes selected by each FS method. The greater the distance between cells that belong to different clusters and the smaller the distance between cells from different cluster, the greater the Silhouette score.

To calculate the Silhouette coefficient for a cell *c* within cluster *C_i_* (out of *n* clusters), the mean distance between the cell and the rest of the cells within the cluster is computed using the gene expression:

\begin{eqnarray*}
a(c) = \frac{1}{|C_i| - 1} \sum _{j \in C_i, c\ne j}d(c,j) \end{eqnarray*}

Then, the minimum mean distance between that cell and the rest of the cells from other clusters is computed:

\begin{eqnarray*}
b(c) = \min _{C_k \ne C_i}\Big \lbrace \frac{1}{C_k} \sum _{j\in C_k}d(i,j) \Big \rbrace \,\,\,\, k \in {1, \cdots , n}
\end{eqnarray*}

Then the Silhouette coefficient is computed as

\begin{eqnarray*}
s(c) = \frac{b(c) - a(c)}{\max {b(c), a(c)}}
\end{eqnarray*}

Higher Silhouette scores imply a better separation between clusters and, therefore, a better performance of the FS method. We used the scikit-learn implementation of Silhouette, sklearn.metrics.silhouette_score.

#### Effect of FS on supervised classification

Two different supervised classifiers (decision tree and KNN classifier from scikit-learn) were trained on the Ding and Mereu benchmarking datasets. Their performance on the different feature selected datasets was measured by computing the 10-fold cross-validation score. The same feature numbers as in the NMI and ARI analysis were used to train the classifiers.

#### Overlap between gene lists

To calculate the overlap between selected features for each FS method, we applied the Jaccard index [43]: $\mathrm{ jaccard}(\mathbf {i},\mathbf {j}) = |\mathbf {i} \cap \mathbf {j}|/|\mathbf {i} \cup \mathbf {j}|$, where $\mathbf {i}, \mathbf {j}$ are the sets of genes selected by the 2 FS methods.

#### Performance of gene selection and locality measures

To assess the performance of different FS methods selecting genes that are relevant for the dataset, we applied 2 different strategies for artificial and biological datasets.

For artificial datasets, we selected 4 representative genes of each of the combinations of genes shown in Supplementary Fig. S4. Then we calculated the mean expression of each of them for genes in each population, and we represent this information in the bar plots.

For benchmarking datasets, to represent Supplementary Fig. S5, for each dataset and FS method we used the following procedure: for each gene, the expression was scaled to sum 1 across all cells. Then, Leiden clustering was run with resolution parameter value 1.2. For each cluster, the proportion of the expression was calculated, and the clusters were ordered so that the first cluster is the one that concentrates the majority of the expression. To create Supplementary Fig. S5, the average value of the proportion of expression is calculated.

#### Proportion of ribosomal and mitochondrial genes

When calculating the proportion of mitochondrial and ribosomal genes, the list of existing ribosomal and mitochondrial proteins was calculated by extracting the genes starting with *RPS, RPL*, or *MT-*. The proportion of mitochondrial or ribosomal genes is the quotient between the genes of the previous list that appear selected by that FS method, and the genes in the list.

#### GO enrichment analysis

To calculate the sets of gene ontologies enriched for the selected features of each FS method, we used Python gseapy (v 0.9.17) module gseapy.enrichr function with the list of the first 1,000 selected features against the GO_Biological_Process_2018 ontology. From the list of enriched ontologies, the 25 with the smallest adjusted *P*-value were selected.

#### Ranking and CD

During calculation of NMI and Silhouette coefficients, to evaluate the overall performance of the FS methods across different datasets, the FS methods are ranked, where 1 is the best rank. The methodology proposed by Demšar [44] is used to test for significant differences among FS methods in the datasets: the Friedman rank test is applied to test whether the mean rank values for all FS methods are similar (null hypothesis). If the Friedman rank test rejects the null hypothesis (α < 0.05), this implies a statistically significant difference among ≥2 FS methods. If the null hypothesis is refuted, we apply the Quade post hoc test between all pairs of FS methods to check which pairs of FS methods are significantly different (α < 0.05). These results are then plotted in a critical difference diagram.

#### Cell subtype quality representation in UMAP

For this analysis we use 2 Ding human PBMC datasets—CELseq2 and Seq-Well. To make the analysis more precise, we subdivided some of the cell types designed in the datasets into different subtypes, based on relevant markers from bibliography, which were also robust across datasets [45–48]. The UMAP coordinates were the ones originally assigned to each dataset, so that we can see the effect of the FS method in the UMAP construction. UMAPs were calculated with min_score=0.1.

The cell type division into cell subtypes was the following: B cells were divided into resting naive B cells (IGHD^+^TCL1A^+^CD79A^+^), proliferative naive B cells (IGHD^+^CD69^+^CCR7^+^), memory B cells (IGHG1^+^CD79A^+^CD27^+^), and plasma cells (MZB1^+^JCHAIN^+^IGHA2^+^); CD14^+^ monocytes were divided into CD14^+^ resting monocytes (CIITA^+^CLEC12A^+^NAIP^+^) and CD14^+^ active monocytes (EGR1^+^IFITM3^+^IER2^+^); CD4^+^ and CD8^+^ T cells were subdivided into 2 additional cell types, γδ T cells (TRDC^+^KLRC1^+^A2M-AS1^+^) and mucosal-associated invariant T cells (SLC4A10^+^DPP4^+^).

To assign these new cell subtypes we used the population-matching algorithm described below. The unsupervised populations used to match the cell subtypes based on their markers were Leiden clusters produced with a high resolution value (8 for both datasets), so that several clusters can be assigned to the same subtype.

### Population-matching algorithm

The aim of this algorithm is to assign a set of clusters to a set of labels, where each label contains a list of representative markers. For each label we extract the matrix of counts of the genes belonging to the label. Then, we create a new matrix, where we assign to each cell and gene the sum of the counts of the gene within its KNN, divided by the number of neighbors. These steps reduce the noisiness of the expression and also increase the local expression of a gene and dampen the expression of sparse genes.

Gene expression values are substituted by the ranked index of their expression, and the values are divided by the largest index to sum 1. Therefore, the cell with the highest expression will have a value of 1 for that gene, while the lowest expressed cell will have a value of near 0. After this normalization is applied to the rest of genes within the label, the mean of the normalized values across genes is computed, so that each cell has a single value for that label.

After the previous steps are computed for the rest of the labels, a new matrix with the number of clusters by the number of labels is computed. For each label and each cluster, the percentile of the normalized values within cells of that cluster is computed (percentile 70 by default). This helps reduce noise on normalized values and assign a unique number per cluster.

This algorithm makes it possible to choose intermediate states, i.e., cell labels with a high similarity. By default, the label with the highest score per cluster is chosen. With the intermediate state option, labels that have a similar value as the label with the highest value are included. The difference in values is set as a threshold (0.05 by default), and labels with difference in value greater than the threshold are not merged.

This algorithm can be found in its corresponding GitHub repository [49] and can be installed via PyPI as pip install cellassign.

## Data Availability

Project name: Triku

Project home page: https://github.com/alexmascension/triku

Notebook repository: https://github.com/alexmascension/triku_notebooks

Notebook output repository: https://doi.org/10.5281/zenodo.5521361

Operating system: Platform independent

Programming language: Python

License: BSD 3

RRID:SCR_020977

An archival copy of the code and notebooks is also available via the *GigaScience* database GigaDB [50].

## Additional Files

**Supplementary Material**

**Supplementary Figure S1**: Comparison of ARI for FS methods on artificial datasets. Bar plots of the ARI for all FS methods with different artificial datasets, using the top 250 (top) and 500 (bottom) features of each FS method. The probability of the selected genes being differentially expressed between clusters (de.prob) is shown in the x-axis. Higher ARI values mean better recovery of the cell populations. Note that in category "all," all features are selected, not the top 250 or 500, therefore their ARI values are the same in both graphs.

**Supplementary Figure S2**: ARI, silhouette of Leiden clusters, and KNN metrics in Ding datasets. Bar plots of the 3 represented metrics. Each bar plot represents the mean over 5 runs, and the error bar is the standard deviation. (A) ARI between clustering solutions and annotated cell types. (B) Silhouette coefficient of Leiden clusters. (C) KNN classifier accuracy using a 10-fold cross validation of annotated cell types, and 10 neighbors. The plot on the left is a critical difference diagram, where each horizontal bar represents the mean rank for all datasets. If ≥2 bars are linked by a gray vertical bar, the mean ranks for those FS methods are not significantly different (Quade test, α = 0.05).

**Supplementary Figure S3**: NMI, silhouette of annotated cell types and decision tree metrics in Mereu datasets. Bar plots of the 3 represented metrics. Each bar plot represents the mean over 5 runs, and the error bar is the standard deviation. (A) ARI between clustering solutions and annotated cell types. (B) Silhouette coefficient of Leiden clusters. (C) KNN classifier accuracy using a 10-fold cross validation of annotated cell types, and 10 neighbors. The plot on the left is a critical difference diagram, where each horizontal bar represents the mean rank for all datasets. If 2 or more bars are linked by a gray vertical bar, the mean ranks for those FS methods are not significantly different (Quade test, α = 0.05).

**Supplementary Figure S4**: Selected features for triku,scanpy,std, and scry in artificial dataset with de.prob 0.01 and 250 genes. The top row shows Wasserstein distance versus log mean expression scatter plots with the features selected by each FS method. The next rows show, for 4 genes, the mean expression per group of cells for each gene. The 4 features selected for each row are represented on the squares on the right: features selected only by triku, by triku and scanpy, by scanpy, by all FS methods, or by std and scry. We see that features selected by triku, with any other combination, have a group of cells where that gene is over- or underexpressed, whereas features selected by other FS methods do not show groups with relevant over- or underexpression.

**Supplementary Figure S5**: Distribution of gene expression across clusters in triku is biased to fewer clusters. For each of the datasets, and each gene, the expression of that gene was scaled to sum 1. Then, for each of the clusters obtained with Leiden (resolution 1.2), the proportion of the whole expression is calculated, and the clusters are ranked, so that the cluster 0 has the highest proportion of expression compared to the rest of clusters. The lines in each plot represent the mean of the proportions for all selected genes for each FS method. For instance, in Ding’s 10X human dataset, the most expressed cluster in features selected by triku expresses, on average, 50% of the expression of the gene, and the one with the second highest level of expression, 20%. Ding’s CELseq2 and inDrop in mouse datasets do not exist and are not shown in the figure.

**Supplementary Figure S6**: Bar plot of *P*-values of GOEA. Each bin represents the number of features selected for each method, in Ding et al. human 10X dataset. The represented value is the −log10 adjusted *P*-value for the best 25 ontologies. On the bottom, the bar plot shows the names of the ontology terms for the case with the best 1,000 features. In immune datasets, gray dots at the left of each term represent that that term is directly related to an immune process. Non-dotted terms refer to more general processes that may or may not be related to immune processes.

**Supplementary Figure S7**: Effect of scatter de.prob parameter on dimensionality reduction. UMAPs of scatter datasets with different de.prob parameter values. UMAP and community detection were done without feature selection. Datasets with de.prob >0.05 are completely resolved in UMAP, whereas lower values make scatter groups less distinguishable.

**Supplementary Figure S8**: Effect of proportion of zeros in gene expression patterns. For each gene, the plot on the left represents the KNN count distribution of that gene, whereas the plot on the right is the UMAP DR representation with the KNN counts for each cell. The genes on the left (*Lyz2, Vtn, Sparc*) have a higher percentage of zeros and are, therefore, expressed in a subset of cells, whereas the genes on the right (*Cog3, Gpr107, Dhx30*) are more thoroughly expressed, and their KNN count distributions are not as heavy-tailed as for the genes on the left.

**Supplementary Figure S9**: Comparison of convolution and read distribution in KNNs. The genes on the left (*Rac2, Vwa1*, and *Opcml*) are candidates to be selected by triku, whereas the genes on the right (*Tpcn2, Usp20, Rbm6*) are not candidates for selection. In each 6-block graph, the column on the left represents the counts (blue histogram) and convolution (orange line) of cells with positive expression, and their KNN; whereas the column on the right represents the counts and convolution of all cells, and their respective KNN. The number within each plot represents the Wasserstein distance between the convolution-based distribution and the KNN count distribution. The dataset used for this visualization is 10X neuron dataset, preprocessed in a similar fashion as the set of benchmarking datasets.

**Supplementary Figure S10**: Robustness of triku parameters. Overlap values for the number of *k* (M1, D1), PCA components (M2, D2), and number of windows (M3, D3) for benchmarking datasets from Mereu et al. [21] (M1, M2, M3) and Ding et al. [20] (D1, D2, D3). Lines in blue represent the overlap between the first 1,000 features, and the extremes of the shaded regions represent the overlap between the first 500 (top) and 2,500 (bottom) features.

**Supplementary Figure S11**: Difference between mean and median correction in triku. Results for Ding mouse SMARTseq2 (A), Ding mouse 10X (B), and Mereu human inDrop (C) datasets. For each dataset, the first plot shows the uncorrected distances on the y-axis, and the log mean expression on the x-axis. The red and blue lines represent the mean and median expression values per window. Dots in blue, red, and dark gray represent selected genes using mean, median correction, or with both cases, respectively. Second and third plots show the corrected distances; therefore the mean and median values are zero. The last plot shows the Jaccard index between the *i* top genes using mean and median correction, for different *i* values ranging from 50 to the default number of selected genes using median (default method).

**Supplementary Table S1**: Number of features automatically selected for each dataset. The cases marked with a hyphen do not exist.

**Supplementary Table S2**: Computation times of different steps of a standard single-cell processing pipeline. For each dataset, different steps of the processing pipelines were performed, and their processing times were computed. Datasets with >1 batch were corrected using harmony and bbknn.

**Abbreviations**
ANOVA: Analysis of variance
FACS: Fluorescence-activated cell sorting
FE: Feature extraction
FS: Feature selection
GO: Gene ontology
GOEA: Gene ontology enrichment analysis
KNN: *k*-nearest neighbors
mRNA: Messenger RNA
NB: Negative binomial
NMI: Normalized mutual information
PBMC: Peripheral blood mononuclear cell
PCA: Principal component analysis
scRNA-seq: Single-cell RNA sequencing
UMAP: Uniform manifold approximation and projection
UMI: Unique molecular identifier

## Competing Interests

The authors declare that they have no competing interests.

### Funding

This work was supported by grants from Instituto de Salud Carlos III (AC17/00012 and PI19/01621), cofunded by the European Union (European Regional Development Fund/European Science Foundation, Investing in your future) and the 4D-HEALING project (ERA-Net program EracoSysMed, JTC-2 2017); Diputación Foral de Gipuzkoa, and the Department of Economic Development and Infrastructures of the Basque Government (KK-2019/00006, KK-2019/00093); European Union FET project Circular Vision (H2020-FETOPEN, Project 899417), Ministry of Science and Innovation of Spain; and PID2020-119715GB-I00 funded by MCIN/AEI/10.13039/501100011033 and by “ERDF A way of making Europe. A.M.A. was supported by a Basque Government Postgraduate Diploma fellowship (PRE_2020_2_0081), and O.I.S. was supported by a Postgraduate Diploma fellowship from la Caixa Foundation (identification document 100010434; code LCF/BQ/IN18/11660065).

### Authors' Contributions

Conceptualization: A.M.A.; Funding Acquisition: M.J.A.-B.; Investigation: A.M.A., O.I.-S., M.J.A.-B., A.I.; Methodology: A.M.A., O.I.-S., II; Project Administration: A.I., M.J.A.-B.; Resources: M.J.A.-B.; Software: A.M.A., O.I.-S.; Supervision: I.I., A.I., M.J.A.-B.; Visualization: A.M.A., O.I.-S.; Writing - Original Draft Preparation: A.M.A., O.I.-S.; Writing - Review and Editing: A.M.A., O.I.-S., I.I., M.J.A.-B., A.I.

## Supplementary Material

giac017_GIGA-D-21-00110_Original_Submission

giac017_GIGA-D-21-00110_Revision_1

giac017_GIGA-D-21-00110_Revision_2

giac017_GIGA-D-21-00110_Revision_3

giac017_Response_to_Reviewer_Comments_Revision_1

giac017_Response_to_Reviewer_Comments_Revision_2

giac017_Response_to_Reviewer_Comments_Revision_3

giac017_Reviewer_1_Report_Original_Submission Christoph Ziegenhain -- 6/9/2021 Reviewed

giac017_Reviewer_1_Report_Revision_1 Christoph Ziegenhain -- 10/8/2021 Reviewed

giac017_Reviewer_2_Report_Original_Submission Rhonda Bacher -- 6/21/2021 Reviewed

giac017_Reviewer_2_Report_Revision_1 Rhonda Bacher -- 10/11/2021 Reviewed

giac017_Reviewer_3_Report_Original_Submission James J Cai -- 6/26/2021 Reviewed

giac017_Supplemental_File
