# Supplementary material for: Triku: a feature selection method based on nearest neighbors for single-cell data
Source: Gigascience. 2022 Mar 12;11:giac017. doi: 10.1093/gigascience/giac017 (PMC8917514; doi:10.1093/gigascience/giac017)
Supplement: giac017_GIGA-D-21-00110_Revision_1 [file giac017_giga-d-21-00110_revision_1.pdf]

GigaScience

Triku: a feature selection method based on nearest neighbors for single-cell data

--Manuscript Draft--

**Manuscript Number:**	GIGA-D-21-00110R1
**Full Title:**	Triku: a feature selection method based on nearest neighbors for single-cell data
**Article Type:**	Research
**Funding Information:**	Instituto de Salud Carlos III (AC17/00012) — Dr. Marcos J. Araúzo-Bravo<br>Instituto de Salud Carlos III (PI19/01621) — Dr. Marcos J. Araúzo-Bravo<br>Diputación Foral de Gipuzkoa (KK-2019/00006) — Dr. Ander Izeta<br>Diputación Foral de Gipuzkoa (KK-2019/00093) — Dr. Ander Izeta<br>Hezkuntza Saila, Eusko Jaurlaritzako (ES) (PRE_2020_2_0081) — Mr. Alex M. Ascensión<br>“la Caixa” Foundation (LCF/BQ/IN18/11660065) — Ms. Olga Ibáñez-Solé<br>European Union FET project Circular Vision ((H2020-FETOPEN, Project 899417)) — Dr. Marcos J. Araúzo-Bravo<br>MICINN/AEI/FEDER, UE ((PID2020- 119715GB-I00) co-funded by the European Regional Development Fund (ERDF/ESF, Investing in your future)) — Dr. Marcos J. Araúzo-Bravo
**Abstract:**	Background, Feature selection is a relevant step in the analysis of single-cell RNA sequencing datasets. Most of the current feature selection methods are based on general univariate descriptors of the data such as the dispersion or the percentage of zeros. Despite the use of correction methods, the generality of these feature selection methods biases the genes selected towards highly expressed genes, instead of the genes defining the cell populations of the dataset.<br>Results, Triku is a feature selection method that favors genes defining the main cell populations. It does so by selecting genes expressed by groups of cells that are close in the k nearest neighbor graph. The expression of these genes is higher than the expected expression if the k cells were chosen at random. Triku efficiently recovers cell populations present in artificial and biological benchmarking datasets, based on ARI, NMI, supervised classification, and silhouette coefficient measurements. Additionally, gene sets selected by triku are more likely to be related to relevant Gene Ontology terms and contain fewer ribosomal and mitochondrial genes.<br>Conclusions, Triku is developed in Python 3 and is available at https://github.com/alexmascension/triku.
**Corresponding Author:**	Marcos J. Araúzo-Bravo<br>Biodonostia Health Research Institute<br>Donostia - San Sebastián, SPAIN
**Corresponding Author Secondary Information:**	
**Corresponding Author's Institution:**	Biodonostia Health Research Institute
**Corresponding Author's Secondary Institution:**	
**First Author:**	Alex M. Ascensión
**First Author Secondary Information:**	
**Order of Authors:**	Alex M. Ascensión<br>Olga Ibáñez-Solé

Powered by Editorial Manager® and ProduXion Manager® from Aries Systems Corporation

	Iñaki Inza
	Ander Izeta
	Marcos J. Araúzo-Bravo
**Order of Authors Secondary Information:**	
**Response to Reviewers:**	Reviewer #1: In the present manuscript, Ascension and colleagues introduce a new feature selection method for scRNA-seq data to increase the relevance of selected genes for downstream analysis such as clustering. I am happy to see that the tool is deposited as an open-source package that seems easy to install and plugs in seamlessly into the commonly used scanpy workflow / AnnData data structure. The documentation is sufficient to get users started. While the method has merit as a smarter approach to feature selection, the manuscript would benefit from some additional work in terms of both text and analysis.<br><br>We thank reviewer #1 for the positive and constructive comments.<br><br>Major points<br>1) While Triku's strategy is being introduced as superior to preexisting methods, it seems that the strong improvements (at least for the NMI summary statistic) in synthetic data turns rather incremental in the real world datasets of Mereu and Deng et al. The authors should discuss reasons for this difference. In the light of small differences and the fact that the performance is only measured in abstract summarized scores, it would be more convincing if the authors presented concrete cases where the application of Triku yields a difference in clustering or downstream analysis of biological relevance. The currently presented Gene Ontology / Geneset enrichment analysis are too diffuse and do not provide the reader with a feeling of the impact Triku could make on their analysis.<br><br>We thank the reviewer for their comment and acknowledge that some complementary clarifications and key references were missing in the original manuscript. We have added those references and modified the manuscript in order to provide the reader with a short overview of the methods that have been used in other articles to validate feature selection methods. We have included both articles introducing novel feature selection methods and articles benchmarking existing tools. We understand that these references were crucial to understand the context of our analysis.<br><br>It is very challenging to obtain evidence of superior performance of a particular feature selection method using biological datasets, for a number of reasons. On the one hand, the intrinsic read distribution of biological datasets is unknown, unlike that of artificial datasets, where it can be set by the user. On the other hand, many datasets differ in the level of granularity used in their annotation (cell types, subtypes, and states). This has an effect on accuracy: the finer the clustering the higher the chance of some clusters being misslabelled. We observe this in Ding PBMC datasets, where major cell types, such as B cells, can be subdivided into more specific cell subtypes—this point is extended later in the response, and in the revised manuscript—. Therefore, when automatic clustering finds clusters referring to minor cell types, some of these clusters will not be assigned to major cell types, and NMI values may be lower. Lastly, we are aware that Ding and Mereu datasets are heterogeneous in nature (6 to 9 different library preparation methods in 3 different tissues –PBMC, cortex and colon–, in human and mouse) and therefore, there will be unrecognizable intrinsic factors that make NMI statistics highly variable.<br><br>This is why most published Feature Selection (FS) methods up to date have either used metrics based on artificial datasets, or metrics on biological datasets while acknowledging that there is no "gold standard" to label cell populations. Here we provide a list of the references that we have added to the manuscript after revision:<br>• Andrews and Hemberg, 2018 [M3Drop/nbumi] used [1] data simulated from either a zero-inflated negative binomial (ZINB) to match three different Smartseq/2 datasets or data simulated from a negative binomial model with variability in library size (LS-NB), and computed AUC; [2] 5 biological datasets and computed Reproducibility (replication between HVG and genes appearing across all datasets) and Importance (Average fold-change in expression of reproducible features).<br>• Brennecke et al., 2013 [Brennecke] used enrichment of GO terms.

Powered by Editorial Manager® and ProduXion Manager® from Aries Systems Corporation

• Townes et al., 2019 [scry] [1] used calculation of ARI on Zheng 4k and 8k datasets' assigned cell types.
• Aevermann et al. 2020 used [1] visual inspection of t-SNE embeddings using the method, all and a random selection of genes.
• Su et al, 2021 used [1] ARI on 12 datasets using different number of features, comparing clustering results between SC3 defaults and SC3 using the selected features.

We believe that the results from any single analysis cannot be taken as a whole measure of truth but, rather, all the results have to be considered as a whole to provide a general view of how the different FS methods work. In that case, although individual results may be more or less conclusive, we have a strong view that triku works among the best for that wide range of metrics. Following the reviewer comment, we have attempted to better characterize the performance of triku using additional validation tests that we have included either in the revised manuscript or in the supplementary material.

Firstly, we have used ARI as a complement to NMI, which is supported in the literature [Freytag et al. 2018, Townes et al. 2019, Lall et al. 2021]. The new results are shown in the figures S2A and S3A of the revised manuscript.

Secondly, we have trained two classifiers (decision tree and kNN) using a mean 10-fold cross validation accuracy, based on [Hemphill, 2014]. The new results are shown in the figures 4C, 5C, S2C and S3C of the revised manuscript.

In order to comply with more biologically relevant tests, we have then calculated the overlap between the DEGs in different Ding Human datasets, and the selected features for different FS methods. This overlap refers to the fraction of DEGs (100 DEGs per leiden cluster) that appears also as selected by the FS method. From what we observe in the following table, for all datasets, all the DEGs are also selected features. Therefore, we believe that using DEG overlap as a representation of goodness of the FS method is not discriminative enough. (See table if the attached pdf of the comments to the reviewers)
Thus, to gain more insight into the biological relevance of the FS methods, we compared UMAP visual appearance on different Ding Human datasets. To do that comparison we performed an in-depth cell subtype characterization, as we observed that the main cell types named in the datasets were not deep enough to appreciate details on the UMAPs. For instance, we divided B cells in 3 subtypes, added gdT and MAIT cells as subtypes of CD4/CD8/NK cells, or divided CD14+ monocytes into resting or active monocytes. All these subdivisions were based on markers shared across datasets and supported by the literature (Wilk et al. 2020, Stewart et al. 2021, Sanz et al. 2019, Shi et al. 2021). The results of this new analysis are shown in Figure 8. We observe that, in comparison with triku, other FS methods tend to mix cell subtypes, thus losing granularity of cell subtype characterization.

The detailed results can be observed in the 1B notebook, section “DEG and UMAP analysis” within the zenodo repository (10.5281/zenodo.4016714).

We hope the added analyses respond adequately to the request of further demonstration on Triku performance.

2) Comparison to other FS methods: Currently, the most widely used method would probably be Seurat's FindVariableFeatures. It would be good to run the presented example data also via Seurat and include it in all comparisons (eg. Fig. 3-6).

We have updated the main figures of the text to include the results from Seurat and ScTransform. From the results we conclude that the performances of Seurat and ScTransform lay within the ranges of scanpy, scry of nbumi for most of the metrics (ARI/NMI/Silhouette scores), and triku is still overall better than the rest of FS methods. These results are shown in the updated Figures 3, S1, 4, S2, 5 and S3, as well as in figures of secondary biological results, such as S5, 6 and S6 of the revised manuscript.

3) Precision of text: There are quite a few statements throughout the text that seem slightly inaccurate and the authors should work in their revision on precision and

Powered by Editorial Manager® and ProduXion Manager® from Aries Systems Corporation

guiding the reader better through the background & performed work with a bit more clarity. Example: discussion of observed zeroes in UMI-data being well described by the Poisson or NB distributions was not realized by Svensson et al but rather had been described several years before. Compare Vieth et al., 2017 Bioinformatics & Chen et al., 2018 Genome Biology

We have now introduced the references stated by the reviewer, as follows: "Early studies observed that the read distribution of most of the single-cell studies could be fitted to a negative binomial (NB) [Vieth et al, 2017]. More specifically, read counts produced a zero-inflated bimodal distribution, whereas UMI counts produced a NB distribution [Chen et al, 2018]. These results were later replicated by Svensson, stating that the proportion of zeros in droplet-based scRNA-seq data, originally assumed to be dropouts, was tightly related to the mean expression of genes, following a NB curve [Svensson, 2020]."

4) One of the main assumptions of Triku is that import genes get "switched on", ie. change their state from rather not expressed to a relatively high expression level. I am wondering if the authors can comment on the performance of Triku in cases where the main difference between cells is a gradual change in already expressed genes and whether such difference might get lost/masked by the selection performed by Triku.

Thanks for this important comment. To answer the reviewer's question, we have applied triku on datasets with differentiation trajectories. The processed datasets are a Pancreas dataset, from Bastidas-Ponce et al. (2019); and a Dentate gyrus dataset, from Hochgerner et al. (2018). Both datasets were downloaded and processed using the scvelo package. We followed the standard processing pipeline to reproduce their findings.

Additionally, we run triku on the processed dataset to get the features of interest, and used the scvelo.tl.rank_velocity_genes() function to obtain the relevant genes involved in the differentiation trajectory. To ensure that different kinds of genes are obtained, we ranged the min_corr parameter between 0.2 and 0.95, in 0.05 intervals.

min_corr parameter refers to the Minimum Spearman correlation coefficient between spliced and unspliced mRNAs. As explained in the RNA velocity basics page (https://scvelo.readthedocs.io/VelocityBasics/):
"The black line corresponds to the estimated ‘steady-state’ ratio, i.e. the ratio of unspliced to spliced mRNA abundance which is in a constant transcriptional state. RNA velocity for a particular gene is determined as the residual, i.e. how much an observation deviates from that steady-state line. Positive velocity indicates that a gene is up-regulated, which occurs for cells that show higher abundance of unspliced mRNA for that gene than expected in steady state. Conversely, negative velocity indicates that a gene is down-regulated."

Therefore, genes with high correlation imply that the unspliced/spliced ratio is proportional and, therefore, that the gene expression is located within the steady-state regime. Thus, that gene is not especially important in the differentiation trajectory. Genes with lower correlation values tend to show a greater unspliced/spliced ratio, and might be drivers of differentiation. The values of correlation cannot be very low (~ 0), otherwise we would be selecting random noise genes.

To compare the genes selected by triku and the genes selected by scvelo, we calculated the ratio of the set of genes detected by triku and scvelo, divided by all the genes selected by scvelo. This ratio, thus, explains the proportion of genes that are associated to differentiation trajectories, and which are selected by triku.

For both the pancreas and the dentate gyrus datasets, for the whole range of min_corr values, this ratio is 1, that is, all the trajectory-relevant genes are also genes detected by triku. Therefore, triku detects all genes associated to differentiation trajectories.

The detailed results can be observed in the 1B notebook, section “Analysis on continuous datasets” within the zenodo repository (10.5281/zenodo.4016714).

These results are expected due to the nature of triku and of the trajectory-related

genes. These genes, despite being expressed in more than one cell type, are somewhat "localized" in comparison to the whole dataset, and therefore are candidates to be selected based on the rationale of triku.

Minor points

5) What is the rationale for selecting the % of zero expression as the descriptive statistics within the knn neighborhood? If a gene occurs in less cells but with higher expression, it's dispersion would be higher too. It would be needed to justify this more precisely and ideally the authors would add a version of Triku that works on dispersion (to show possible differences).

Our intention is not to use the percentage of zeros as the descriptive statistic within the kNN neighbor. We observed that excluding (or including) zeros to calculate the observed and expected count distributions for the calculation of the Wasserstein distance could have a direct effect on the distance calculation. For more information we kindly ask the reviewer to revise the “Why cells with zero counts are not considered to build the kNN count distribution” section in Supplementary Results.

Additionally, the percentage of zeros proposed in the rationale is already an accurate descriptor that acts in the same manner -if not better- than only the dispersion. For instance, in the example provided by the reviewer, a gene with high expression in fewer cells would yield a high percentage of zeros. On the other hand, a gene with low expression in few cells, in spite of having a high percentage of zeros and a low dispersion, can be a candidate for election by Triku if the expression is localized in a small subset of similar cells. In fact, this effect was described before (Wu and Zhang, 2020): "Despite having low overall expression levels within a cell, such genes [transcription factors] might be important in uncovering the fate of that cell.".

The use of the dispersion might only be inefficient for gene classification. In Supplementary Figure 8 we now show examples of genes with low, medium and high expression levels and similar dispersions, but different colocalization levels. For each level of expression, it is easy to find genes that are expressed in similar cells, and genes expressed in dissimilar cells. These later genes would not be discerned by using dispersion-based FS methods alone.

We kindly ask the reviewer to consult the 2A notebook in our zenodo repository (10.5281/zenodo.4016714) to see extended information on the rationale and initial design stages of triku.

6) Three main types of feature selection methods are introduced but not defined/explained further (p. 2)

We have included a more precise description of the methods.

7) Since Triku performs more calculations/steps than existing methods for FS, the runtime is presumably higher. The authors should compare and comment on runtime.

Thank you very much for this important remark. Following your suggestion, we have spent some more time trying to optimize the computational efficiency of our method, which has resulted in a new version (v2) of the method with improved running times. Additionally, we have added a discussion on the runtime efficiency of our method in the supplementary materials.

Computational cost optimization was done by enabling triku to work both with dense and sparse matrices, as well as by identifying and resolving bottlenecks. We also made the decision to rely the k-NN graph calculation to the original from scanpy (which also uses UMAP neighbors’ function to calculate the graph). This decision was based on two factors. Firstly, oftentimes the neighbor graph is previously computed; therefore, computing it again is a waste of time that can be avoided by directly using the graph computed by scanpy. Secondly, if the user runs the neighbor graph using scanpy, and this graph is again computed using triku, the graphs might be different (due to a different default number of neighbors, or due to intrinsic differences in the heuristics of neighbor calculation of both methods). This makes the results less reproducible, since the graph computed by triku is discarded once the method is run. Therefore, for v2 we

Powered by Editorial Manager® and ProduXion Manager® from Aries Systems Corporation

made the decision to deprecate PCA/kNN graph calculation, using the graph computed by scanpy instead. If the graph is not computed, the user has to compute it first (an error is raised).

Regarding the cost-efficiency of triku compared to other feature selection methods, we must consider that most feature selection methods rely on the calculation of a single univariate measurement, whose calculation is virtually immediate. However, other steps in single-cell RNAseq analysis pipelines are much more time consuming. We consider that triku has an acceptable running time, compared to other steps from a common single-cell RNAseq processing pipeline and that its use does not perceivably increase the overall running time of the analysis. In order to show this, we downloaded datasets with a range of cells (901 to 242106), also considering different batches, and ran a standard processing pipeline consisting of the following steps: QC and filtering, PCA, kNN, triku, UMAP/t-SNE, leiden/louvain, and PAGA. For datasets with batches, bbknn and harmony are used.

In general, we observed that triku shows similar runtimes to other steps in the processing pipeline, such as PCA or kNN. The results of this analysis can be found in the section “triku processing times” in the Supplementary Materials.

Reviewer #2: The manuscript presents a new method, Triku, for feature selection in single-cell RNA-seq data. Feature selection is performed upstream of tasks such as clustering and differential expression to reduce the effect of genes with noisy expression. Triku uses a KNN approach to identify features that are unexpected within cells that are transcriptionally close. Overall, the manuscript is well-written, presented clearly, and is a promising new method for feature selection. The figures are also very nice.

We thank reviewer #2 for the positive and constructive comments.

Major:
1. In Figure 4, it is not obvious why different methods would rank so differently between the two datasets. What methods did those papers originally use for feature selection (if available). Does that partially explain the differences?

The authors of the original papers state the following in their methods section:

- Ding et al.: "We then performed principal-component analysis using all genes, did clustering analysis (Louvain clustering of the k-nearest neighbor (k-NN) graph built from the first 50 principal components [...] followed by differential gene expression analysis with the FindAllMarkers command in Seurat to find cluster-specific (up-regulated) marker genes."
- Mereu et al.: "We selected HVGs by evaluating the relationship between gene dispersion (y.cutoff = 0.5) and the log mean expression"

Thus, Ding et al. do not use any form of feature selection (FS) method, since FindAllMarkers is a differentially expressed gene selection method; and Mereu et al. apply a method based on dispersion, such as the one based in Scanpy or Seurat. Therefore, the differences of the methods cannot be directly explained by the FS method used, since otherwise the method "all" (use all genes) would rank the highest in Ding et al., and methods such as scanpy or seurat would rank highest. However, this is not the case. We believe that the main driver in the results is the decision of cell labelling which, being semi-supervised, might not be completely accurate in the sense that some important cell subtypes might be missing (because they would be merged).

2. Figure 6, the left-most plot does not belong? It is not described in the legend.

We apologize for this omission. We have now updated the caption of the figure to explain the subplot referring to the NMI values of the datasets.

3. It would be helpful to note somewhere which category of methods the others belong to (i.e. variance based or distribution based).

We have updated the information in the Materials and Methods section of the paper.

Powered by Editorial Manager® and ProduXion Manager® from Aries Systems Corporation

Now, in the presentation of the FS methods we include the category they belong to.

4. Some additional results and discussion on the number of genes selected. 250-500 is quite low and may explain the poor overlap between genes selected. In my experience with commonly used methods from the scran or Seurat package a more typical number of genes selected is around 2,000. What are the typical numbers used/recommended for the other methods compared to here? Does the performance difference remain when expanded to the top 2,000 genes? And is the performance better for Triku on 250 compared to 2,000?

The case of 250-500 does only apply to artificial datasets generated by scatter. Those datasets are not used for the creation of the overlap figure. Instead, for that figure, the genes selected are the ones automatically detected by triku. As referenced now in Supplementary Figure 1, the number of selected features for most of the datasets are in the range 1500 to 3000, which are commonly used values. Therefore, we consider that the poor overlap between features is justified based on the number of features.
As for the election of 250 to 500 features in artificial dataset, we made the choice based on the fact that the count properties (mean, distribution, noise, outliers, etc.) of artificial datasets differ from biological ones, and require a lower set of features to explain the variability of the dataset. We performed some analyses and observed that, for datasets with poor overlaps (0.0065 - 0.025) the number of automatically-selected features ranged between 400 and 700 features, which is in range with the examples of 250 and 500 features.

5. In methods, "By default, the number of features is the one automatically selected by triku." These values should be put into the supplement to get a better idea of how many genes are being selected by default.

According to the reviewer's suggestions, we have added in Supplementary Table 1 the number of features selected for each method.

Minor:
1. In Figure 3, I would label the top and bottom as A and B, I initially misread the legend as top 250 and bottom 500 genes.

For improved clarity, the suggested changes have been applied in the corrected figure.

2. What are the approximate run times a user can expect for this method?

Please see the answer to the related point 7 of reviewer #1.

Reviewer #3: This manuscript introduces a k-NN based feature selection method, Triku, as one key step to secure informative features in analyzing single cell RNA sequencing datasets. The authors argue that most of the current feature selection methods bias to the highly expressed genes instead of the actual gene markers defining the cell populations. Instead, they focus on the local signature of gene expression for each gene and compute how each of them deviates from their null distributions. The ranked gene list concerning the deviation will be derived after the median correction. The authors use Silhouette coefficient to validate their conclusion of better modularity by comparing to other methods. Additionally, the randomness and the robustness of the method are well discussed. In general, this article is well-organized and well-written. The examples of artificial and benchmark datasets showing certain aspects of improvements compared to current methods are illustrative.

Triku will be a valuable contribution to the single cell analysis field.

We thank reviewer #3 for the positive and constructive comments.

The reviewer has some minor comments to help improve the manuscript further:
1. The authors compare Triku to many other widely-used benchmark methods but excluding Seurat. Although Seurat method is adopted in Scanpy, as they claim in the "FS methods", the default flavor of Scanpy is "Seurat" instead of "Seurat_v3", the default feature selection method in the latest version of Seurat. It might be good to

make it clear. Also, another alternative yet popular method, sctransform, from Seurat is not on the comparing list.

We have updated the main figures of the text to include the results from Seurat and ScTransform. From the results we conclude that Seurat and ScTransform lay within the ranges of scanpy, scry of nbumi for most of the metrics (ARI/NMI/Silhouette scores), and triku is still overall better than the rest of FS methods. These results can be observed in the updated Figures 3, S1, 4, S2, 5 and S3, as well as in figures of secondary biological results, such as S5, 6 and S6 of the revised manuscript.

2. The evidence of "we observed that in certain datasets the Wasserstein distances tend to slightly increase with the mean expression of the genes" could be shown to introduce the necessity of further correction. And the reason why the median correction outperforms other correction methods is left unexplained. For example, Seurat, which also considers binning correction method, uses mean to control the strong relationship between variability and average expression.

The choice of using median for correction instead of mean is because we could not assume that the distribution of Wasserstein distances per bin is normally distributed, hence a non-bias central descriptor is more appropriate. Nonetheless, in the version 2.1.2 of the package we have included the option for the user to choose between mean and median correction.

The comparison between mean and median for distance correction can be observed in the section “Difference between mean and median for window correction” of Supplementary Materials.

We see that for some datasets this trend is apparent and can affect gene selection, that is, genes with higher mean will be selected only because of having higher mean, which is somewhat related to the Wasserstein distance. Regarding the use of mean or median, we do not observe abrupt differences. In some cases, using mean selects highly-expressed genes. However, the overlap of selected genes (by Jaccard index) is always greater than 0.9, which implies that the most relevant genes are always selected.

3. Since the authors integrate into the pipeline the k-NN module, which is considered computationally expensive, it would be great to evaluate the time complexity/running speed compared with other methods.

Please see the answer to the related point 7 of reviewer #1.

4. Triku assumes that the local transcriptomic similarity is more likely to define cell types. Apart from clustering, which might be better-quality after Triku, it would be interesting to show any potential effects to other popular downstream analyses in the single cell field, such as trajectory inference, given that Triku is subject to locality.

Please see the answer to the related point 4 of reviewer #1.

5. Triku builds k-NN graph on UMAP all the way around. To validate the robustness of Triku, one could also discuss alternative low embedding methods like t-SNE in the section of "robustness".

In the latest version of triku the neighbor graph is delegated to scanpy, which by default uses also UMAP. The name UMAP in both the previous manner of choosing the neighbors, and the one implemented by scanpy, are not related to the dimensionality reduction method by itself, but rather by the fact that this neighbor graph method and the dimensionality reduction methods are located within the same package.

6. Since Triku is likely to identify locally over-expressed genes, it would be interesting to see the overlap between features selected by Triku and the differential expressed genes, if the setting is possible to arrange to make the two comparable.

Please see the answer to the related point 1 of reviewer #1.

Powered by Editorial Manager® and ProduXion Manager® from Aries Systems Corporation

	7. In the section of previous work, some claims were made without references. For instance, "Early methods for FS in scRNA-seq data were based on the idea that genes whose expression show a greater dispersion across the dataset are the ones that best capture the biological structure of the dataset". Another example of relevant references missing is https://pubmed.ncbi.nlm.nih..<br><br>We apologize for this omission. We have now added support to that claim using the papers by Brennecke et al. (2013) and Osorio et al. (2019). "Early methods for FS in scRNA-seq data were based on the idea that genes whose expression show a greater dispersion across the dataset are the ones that best capture the biological structure of the dataset [Brennecke et al, 2013, Osorio et al, 2019]."<br><br>8. Fig. S2 does not show exact gene names. For artificial data, why those four genes are representative is left unexplained.<br><br>The exact gene names are not relevant because, for being artificial, they are represented simply as numbers. As for the choice of those genes specifically, we tested the effect on many other gene combinations and, after seeing that they had the same effect, we chose one of the combinations of 4 genes to keep the reproducibility of the plots.<br><br>9. The authors classify reference 11, the dropout-based method as "a new generation". As far as I know, the benchmark M3Drop was published in 2018.<br><br>We have modified the statement to the following: "This finding opened the path for new FS methods that would rely on genes that showed a greater than expected proportion of zeros, according to their mean expression."
**Additional Information:**	
**Question**	**Response**
Are you submitting this manuscript to a special series or article collection?	No
**Experimental design and statistics**<br><br>Full details of the experimental design and statistical methods used should be given in the Methods section, as detailed in our Minimum Standards Reporting Checklist. Information essential to interpreting the data presented should be made available in the figure legends.<br><br>Have you included all the information requested in your manuscript?	Yes
**Resources**<br><br>A description of all resources used, including antibodies, cell lines, animals and software tools, with enough information to allow them to be uniquely identified, should be included in the Methods section. Authors are strongly	No

encouraged to cite Research Resource Identifiers (RRIDs) for antibodies, model organisms and tools, where possible.<br><br>Have you included the information requested as detailed in our Minimum Standards Reporting Checklist?	
If not, please give reasons for any omissions below.<br><br>as follow-up to "**Resources**<br><br>A description of all resources used, including antibodies, cell lines, animals and software tools, with enough information to allow them to be uniquely identified, should be included in the Methods section. Authors are strongly encouraged to cite Research Resource Identifiers (RRIDs) for antibodies, model organisms and tools, where possible.<br><br>Have you included the information requested as detailed in our Minimum Standards Reporting Checklist?<br><br>"	The research does not involve any use of organisms or antibodies.
**Availability of data and materials**<br><br>All datasets and code on which the conclusions of the paper rely must be either included in your submission or deposited in publicly available repositories (where available and ethically appropriate), referencing such data using a unique identifier in the references and in the "Availability of Data and Materials" section of your manuscript.<br><br>Have you have met the above requirement as detailed in our Minimum Standards Reporting Checklist?	Yes

Powered by Editorial Manager® and ProduXion Manager® from Aries Systems Corporation

Manuscript
Click here to access/download;Manuscript;Triku_main_2021_09_24.pdf

OXFORD (GIGA)^n SCIENCE

GigaScience, 2017, 1–15

doi: xx.xxxx/xxxx
Manuscript in Preparation
Research

RESEARCH

Triku: a feature selection method based on nearest neighbors for single-cell data

Alex M. Ascensión[1, 2†], Olga Ibáñez-Solé[1, 2†], Iñaki Inza[3], Ander Izeta[2] and Marcos J. Araúzo-Bravo[1, 4, 5,*]

[1]Biodonostia Health Research Institute, Computational Biology and Systems Biomedicine Group, Paseo Dr. Begiristain, s/n, Donostia-San Sebastian, Spain and [2]Biodonostia Health Research Institute, Tissue Engineering Group, Paseo Dr. Begiristain, s/n, Donostia-San Sebastian, Spain and [3]Intelligent Systems Group, Computer Science Faculty, University of the Basque Country, Donostia-San Sebastian, Spain and [4]Max Planck Institute for Molecular Biomedicine, Roentgenstr. 20, 48149, Muenster, Germany and [5]IKERBASQUE, Basque Foundation for Science; Bilbao, Spain.

*mararabra@yahoo.co.uk
†Contributed equally.

Abstract

Background, Feature selection is a relevant step in the analysis of single-cell RNA sequencing datasets. Most of the current feature selection methods are based on general univariate descriptors of the data such as the dispersion or the percentage of zeros. Despite the use of correction methods, the generality of these feature selection methods biases the genes selected towards highly-expressed genes, instead of the genes defining the cell populations of the dataset.
Results, Triku is a feature selection method that favors genes defining the main cell populations. It does so by selecting genes expressed by groups of cells that are close in the k nearest neighbor graph. The expression of these genes is higher than the expected expression if the k cells were chosen at random. Triku efficiently recovers cell populations present in artificial and biological benchmarking datasets, based on ARI, NMI, supervised classification, and silhouette coefficient measurements. Additionally, gene sets selected by triku are more likely to be related to relevant Gene Ontology terms and contain fewer ribosomal and mitochondrial genes.
Conclusions, Triku is developed in Python 3 and is available at https://github.com/alexmascension/triku.

Key words: sc-RNAseq, feature selection, machine learning, bioinformatics, python

Introduction

Single-cell RNA sequencing (scRNA-seq) is a powerful technology to study the biological heterogeneity of tissues at the individual cell level, allowing the characterization of new cell populations and cell states–i.e. cell types responding to different environmental stimuli– previously undetected due to their low frequency within the tissue and the lack of individual resolution of bulk methods [1, 2].

scRNA-seq datasets are multidimensional, i.e. the expression profile per cell consists of multiple genes. Two common characteristics of multidimensional datasets are their high dimensionality and their sparsity, which are worsened in single-cell datasets due the high proportion of zeros from low signal recovery [3]. This sparsity affects downstream methods such as cell type detection or differential gene expression [4].

A common task when working with multidimensional datasets is feature selection (FS). FS, alongside with feature extraction (FE), responds to the need of obtaining a reduced dataset with a smaller dimensionality [5]. While FE methods

Compiled on: September 24, 2021.
Draft manuscript prepared by the author.

like Principal Component Analysis (PCA) extract new features based on combinations of the original features, FS methods aim to select a subset of the features that best explains the original dataset.

There are three main types of FS methods: filter, wrapper and embedded methods [5].

- Univariate filter methods look at intrinsic properties of the data (e.g., variance, correlation), calculating a univariate score per feature, ranking them and removing the low-scoring ones. These techniques are usually fast and scalable, and are independent of downstream methods. Common examples of univariate filter techniques are selection by variance, χ^2, t-test, ANOVA, or information gain ratio.
- Wrapper methods embed the subsequent supervised algorithm within the feature search process. They perform an heuristic search in the space of feature subsets and score each subset of features with the scoring associated to the classification model. A common wrapper method type is genetic algorithms.
- Embedded methods include the search of the subset of features within the model construction. A common embedded method type is decision trees.

Current FS methods in scRNA-seq analysis are filter methods because common downstream analysis steps do not embed the FS within the pipeline [6]. FS methods represent a key step in processing pipelines of bioinformatic datasets [7] and provide several advantages [5]: they reduce model overfitting risk, improve clustering quality, and favour a deeper insight into the underlying processes that generated the data (features –genes– that contain random noise do not contribute to the biology of the dataset and are removed). Specifically, in scRNA-seq, removing non-informative features can improve results in downstream analyses such as differential gene expression.

Early methods for FS in scRNA-seq data were based on the idea that genes whose expression show a greater dispersion across the dataset are the ones that best capture the biological structure of the dataset [8, 9]. Conversely, genes that are evenly expressed across cells are unlikely to define cell types or cell functions in a heterogeneous dataset. The most straightforward way of selecting genes that are not evenly expressed is to look at a measure of dispersion of the counts of each gene and to select those genes that have a dispersion over a threshold.

However, the correlation between mean expression and dispersion introduces a bias whereby genes with higher expression are more likely to be selected by FS methods. However, biological gene markers that define minor cell types are usually expressed in a medium to small subset of cells. Therefore, new FS methods based on dispersion are designed to correct for this dispersion/expression correlation to select genes with a broader expression spectrum.

Brennecke et al. [8] developed a FS method that introduces a correction over the dispersion that accounts for differences in the mean expression of genes. It does so by setting a threshold to the correlation between the average gene expression and its coefficient of variation across cells. Newer FS methods have arisen after different corrections, like the one originally described by Satija et al. [10] implemented in Seurat, later adapted to *scanpy* [11], a later evolution of the method developed in sctransform [12], or the one implemented in *scry* [13].

Early studies observed that the read distribution of most of the single-cell studies could be fitted to a negative binomial (NB) [14]. More specifically, read counts produced a zero-inflated bimodal distribution, whereas UMI counts produced a NB distribution [15]. These results were later replicated by Svensson, stating that the proportion of zeros in droplet-based scRNA-seq data, originally assumed to be dropouts, was tightly related to the mean expression of genes, following a NB curve [16]. Genes with an expected lower percentage of zeros tend to have an even expression across the entire set of cells. Conversely, genes with a higher than expected percentage of zeros might possess biological relevance because they are expressed in fewer cells than expected, and these cells might be associated to a specific cell type or state.

This finding opened the path for new FS methods that would rely on genes that showed a greater than expected proportion of zeros, according to their mean expression. These methods are based on a null distribution of some property of the dataset, and genes whose behavior differs from the expected are selected. The FS method *nbumi*, a negative binomial method based on *m3drop* [17], works under this premise. *nbumi* fits the NB zero-count probability distribution to the dataset, and selects genes of interest calculating p-values of observed dropout rates. *m3drop* works similarly by fitting a Michaelis-Menten model instead of the NB from *nbumi*.

In summary, existing FS methods assume that an unexpected distribution of counts for a particular gene in a dataset is explained by cells belonging to different cell types. However, we observe that there are three main patterns of expression according to the distribution of zeros of a particular gene and overall transcriptional similarity (expression of all genes), as explained in detail in Figure 1: a) a gene evenly expressed across cells, or a gene expressed by a subset of cells, which can be b1) transcriptomically separate or b2) transcriptomically similar. Thus, in some cases a particular gene shows an unexpected distribution of counts because a subset of cells are expressing it but those cells might not be transcriptomically similar.

Here we present *triku*, a FS method that selects genes that show an unexpected distribution of zero counts and whose expression is localized in cells that are transcriptomically similar. Figure 2 summarizes the feature selection process. *Triku* identifies genes that are locally overexpressed in groups of neighboring cells by inferring the distribution of counts in the vicinity of a cell and computing the expected distribution of counts. Then, the Wasserstein distance between the observed and the expected distributions is computed and genes are ranked according to that distance. Higher distances imply that the gene is locally expressed in a subset of transcriptomically similar cells. Finally, a subset of relevant features is selected using a cutoff value for the distance. *Triku* outperforms other feature selection methods on benchmarking and artificial datasets, using unbiased evaluation metrics such as Normalized Mutual Information (NMI) or Silhouette. Of note, features selected by *triku* are more biologically meaningful, as compared to other methods.

Results

The objective of FS methods is to select the features that are the most relevant in order to understand and explain the structure of the dataset. In the context of single-cell data, this means finding the subset of genes that, when given as input to a clustering method, will yield a clustering solution where each cluster can be annotated as a putative cell type.

Initially, we generated artificial datasets with the *splatter* package [18], so that cells belonging to the same cluster have a similar gene expression. All datasets contained the same number of genes, cells and populations, but differed in the `de.prob` parameter value. This parameter was set so that higher values indicate a higher probability of genes being differentially expressed, resulting in more resolved populations. A combination of 8 `de.prob` values, from 0.0065 to 0.3 were used (see

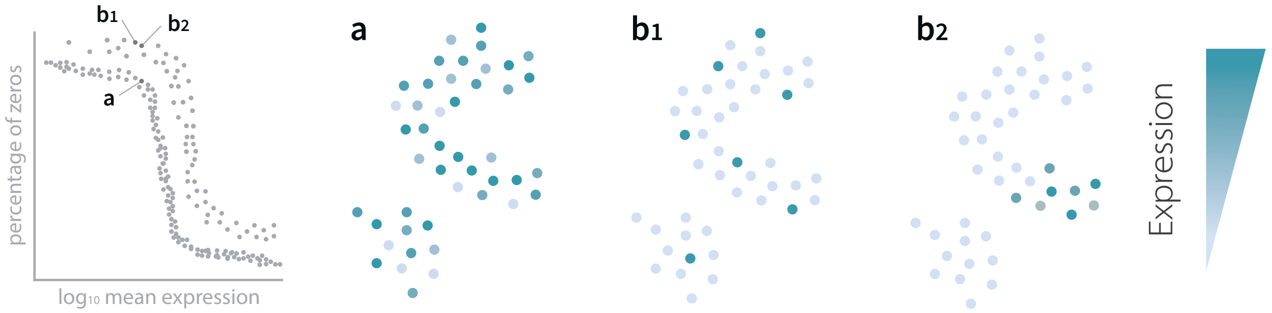

Figure 1. Distribution of gene expression in three scenarios. There are three main patterns of expression for any particular gene in a single-cell dataset: a) The gene is expressed evenly across cells in the dataset, which probably means it does not define any particular cell type. b) A gene shows an unexpected distribution of zeros, because it is only expressed by a subset of cells. Within case b, there are two possible patterns. b1) The gene is highly expressed by a subset of transcriptomically different cells (i.e. cells that are not collocalized in the dimensionally reduced map) and b2) the gene is highly expressed by cells that share an overall transcriptomic profile. *Triku* preferentially selects the genes shown in the b2 pattern. When looking at the proportion of zeros, genes in cases b1 and b2 show an increased proportion of zeros with respect to a, but they are indistinguishable from each other by that metric.

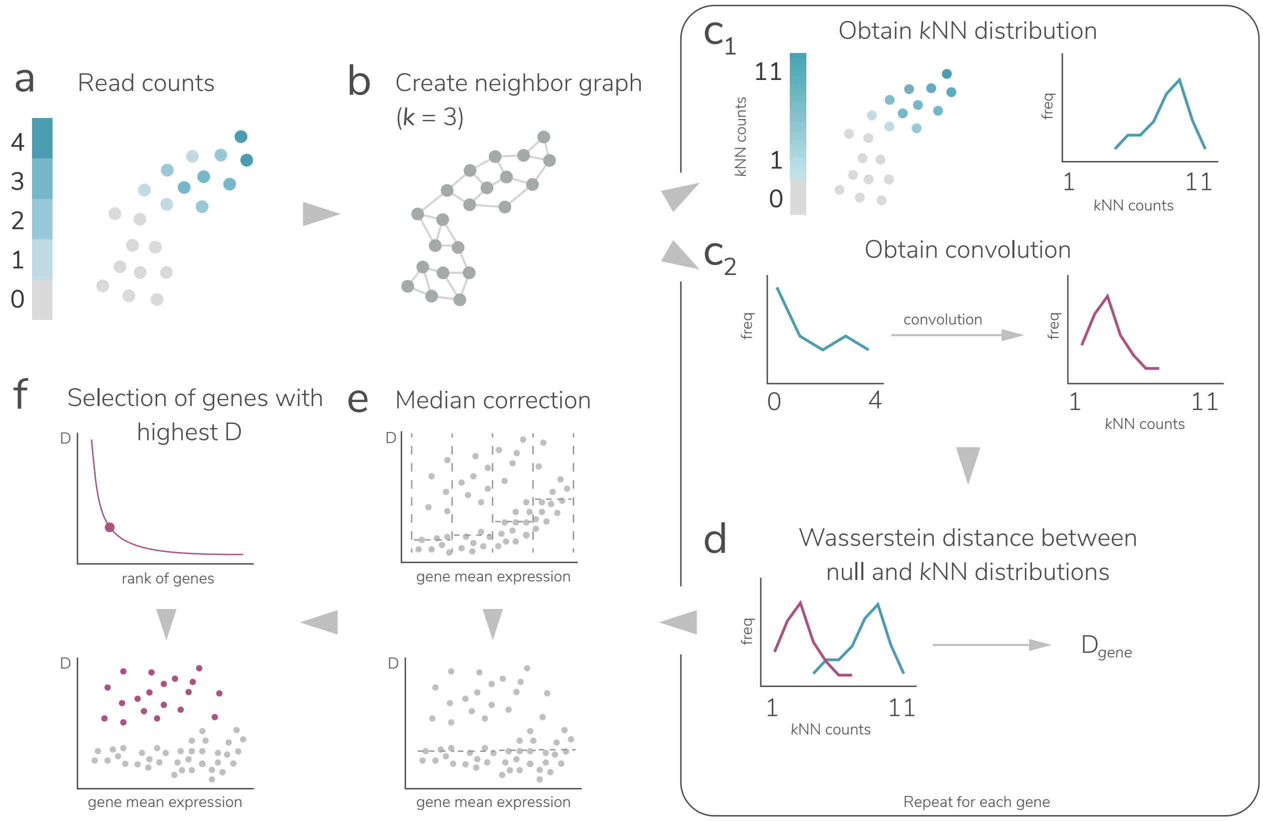

Figure 2. Graphical abstract of *triku* workflow. a) DR representation of the gene expression from the count matrix from a dataset, where each dot represents a cell. b) *k*NN graph representation with 3 neighbors. For each cell the *k* transcriptomically most similar cells are selected (3 in this example). c1) Considering the graph in b) for each cell with positive expression, the expression of its *k* neighbors is summed to yield the *k*NN distribution in blue. c2) With the distribution of reads (blue line), the null distribution is estimated by sampling *k* random cells. d) The null and *k*NN distributions of each gene are compared using the Wasserstein distance. e) For each gene, its distance is plotted against the log mean expression, and divided into *w* windows (4 in this example). For each window, the median of the distances is calculated and subtracted to the distances in that window. f) All corrected distances are ranked and the cutoff point is selected.

Methods). In addition, we tested *triku* on two biological benchmarking datasets by Ding et al. [19] and Mereu et al. [20] that have been expert-labeled using a semi-supervised procedure. Both benchmarking datasets are composed of individual subsets of data with different library preparation methods (10X, SMART-seq2, etc.) in human Peripheral Blood Mononuclear Cells (PBMCs) (Mereu and Ding) and mouse colon (Mereu) and cortex (Ding) cells.

We have evaluated the relevance of the features selected by *triku* by comparing them to the ones selected using other feature selection methods, similar to [8, 13, 17, 7]. The relevance of the features was first measured using metrics associated to the efficacy of clustering, and then using metrics to evaluate the quality of the genes selected.

We made six types of comparisons between the subsets of genes selected by each feature selection method: 1) the ability to recover basic dataset structure (main cell types) in artificial and biological datasets, 2) the ability to obtain transcriptomically distinct cell clusters, 3) the overlap of features between different FS methods, 4) the localized pattern of expression of

the features selected, 5) the ability to avoid the overrepresentation of mitochondrial and ribosomal genes, 6) the biological relevance of the genes by studying the composition and quality of the gene ontology (GO) terms obtained and 7) the resolution of cell types and subtypes on the UMAP.

triku efficiently recovers cell populations present in scRNAseq datasets

The first set of metrics evaluates the ability to recover the original cell types based on the NMI index, and the cluster separation and cohesion using the Silhouette coefficient.

NMI

NMI measures the correspondence between a labeling considered as the ground truth and the clustering solution that we obtained using the genes selected by *triku* and other FS methods.

First, we evaluated how well the clustering using the genes selected by the FS methods was able to recover the same populations that were defined when generating the artificial datasets. Figure 3 shows that *triku* is among the best three feature selection methods for a wide range of `de.prob` values. For low values of `de.prob` –below 0.05–, where the selection of genes that lead to a correct recovery of cell populations is more challenging, *triku* notably outperforms the rest of the FS methods. NMI values obtained with *triku* are 0.1 to 0.2 higher than the second and third best FS methods. In addition, the results obtained when using the first 250 selected genes were comparable to those obtained when selecting 500 genes, showing that this efficiency is independent of the amount of genes chosen within a sensible range. These results are replicated using the adjusted Rand index (ARI), used by others [13, 21, 22], in Supplementary Figure S1.

We also studied how well the selected genes led to a clustering solution that was similar to the manually-assigned cell labels in the biological benchmarking datasets, as shown in Figures 4A and 5A. For each dataset, the variability between NMI scores was quite low, meaning that features selected with the different methods yielded clustering solutions that were quite similar to the manually-labeled cell types, although there are some exceptions to this rule–e.g. *brennecke* in Ding datasets, or *scanpy* in some Mereu datasets, which showed notably reduced NMI values–. In some datasets, for instance, 10X human, QUARTZseq human and SMARTseq2 human from Mereu's benchmarking set, features selected by FS methods did not lead to increased NMI values as compared with randomly selected genes.

Despite the differences in NMI between methods being small for each particular dataset, post-hoc analysis revealed that *triku* is the best ranked method across all datasets. To do the post-hoc analysis, we ranked for each dataset the NMI of each FS method. Figure 4A and 5A (left) shows the mean rank of each FS method across datasets. *Triku* is the best-ranked FS method in both Mereu and Ding benchmarking datasets, with a mean rank of 3.2 and 3.7, respectively. *m3drop* is the second and third best-ranked FS method for Mereu and Ding datasets, respectively. For Mereu datasets this difference is statistically significant, whereas for Ding datasets, the difference is not statistically significant compared to the second best method, *m3drop*, but it is significant compared to the rest (Quade test, $p < 0.05$).

Supplementary Figures S2A and S3A show similar results using ARI instead of NMI, where *triku* is statistically significantly the best FS method for Mereu datasets, and the best together with *m3drop* for Ding datasets.

Silhouette coefficient

Another important aspect of the genes selected by FS methods in scRNA-seq data analysis is their ability to cluster data into well-separated groups that are transcriptomically similar. We used the Silhouette coefficient to measure the compactness and separation-degree of cell communities obtained with a clustering method. When the same clustering algorithm is used on a dataset but using two different FS methods, the differences in the resulting Silhouette coefficients can be entirely attributed to the features selected by those methods. We assume that FS methods that increase the separation between clusters and the compactness within clusters are better at recovering the cell types present in the dataset.

Figures 4B and 5B show the Silhouette coefficients obtained with the different FS methods. For the Mereu and Ding datasets, we observed that *triku* was the best-ranked method–mean rank of 3.1 and 2.1–, and the second best-ranked methods were *m3drop* and *scanpy* with a mean rank of 3.9 and 3.5, respectively. In Ding datasets, the difference between *triku* and the second-ranked method was statistically significant (Quade test, $p < 0.05$).

We performed an additional analysis using the labels obtained with leiden clustering instead of the manually curated cell types (Supplementary Figures S2B and S3B). Again, *triku* outperformed the rest of the FS methods showing a statistically significant best mean-rank, for both benchmark datasets.

Supervised cell type classifiers

As an additional measure of FS method accuracy, we trained two classifiers –decision tree and *k*NN– using a 10-fold cross validation, as shown in [23]. The results for decision tree classifier in Ding and Mereu are shown in Figures 4C and 5C; and the results for *k*NN classifier are shown in Supplementary Figures S2C and S3C.

In general, classifier efficiency shows high variance, even more for Mereu datasets, and therefore the results are not completely conclusive. From the critical difference diagrams, we can state that *triku* is in range with most of the FS methods for decision tree and *k*NN classifier. In general, we see a decrease of efficiency in Ding datasets for *seurat*, *sct*, and sometimes *m3drop*; which act similar to a random choice of features; whereas efficiency of *brennecke* is worse than a random choice of features.

For *triku*, *nbumi*, *scry*, *std*, and selecting all features, their efficiencies are similar.

Despite the 10-fold cross validation accuracy of decision trees ranking the highest when trained on the whole set of features, the actual difference in performance between triku and using all features is negligible. Overall, FS does not have a strong impact on classifier accuracy.

Genes selected by different FS methods show limited overlap

Next, we studied the characteristics of the genes selected by *triku* and compared them to the genes selected by other methods.

Initially, we studied the level of consistency between the results obtained using different FS methods by studying their degree of overlap, as shown in Figure 6. In order to compare between equally-sized gene lists, we ranked the genes based on *p*-values or scoring value from each FS method and set the number of genes selected by *triku* as a cutoff to select the first genes. Although the genes selected by the different methods yielded clustering solutions that are highly consistent, as shown in the previous section, we did not see any clear gene overlap pattern between pairs of FS methods. Actually, there is no correlation

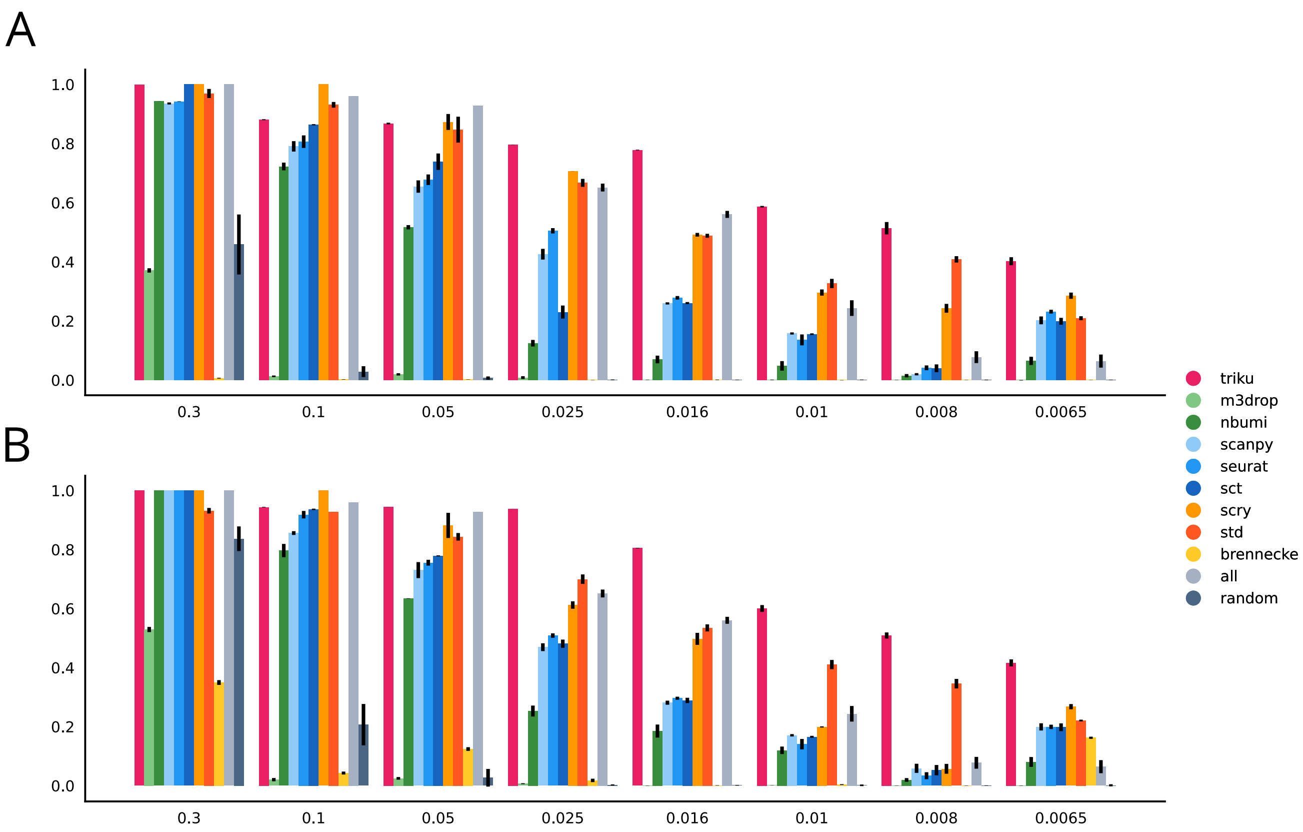

Figure 3. Comparison of NMI for FS methods on artificial datasets. Barplots of the NMI for all FS methods with different artificial datasets, using the top 250 (top) and 500 (bottom) features of each FS method. The probability of the selected genes being differentially expressed between clusters (*de.prob*) is shown in the X axis. Higher NMI values mean better recovery of the cell populations. Note that in category *all*, all features are selected, not the top 250 or 500, therefore their NMI values are the same in both graphs.

between the degree of overlap between the genes selected by the different methods and the clustering solutions that are obtained when using those genes as input.

For instance, we found an overlap of 7% between the genes selected by *scanpy* and *std* for the 10x mouse dataset, yet the NMI between the clustering solutions obtained with each of them and the expert-labeled cell types was 0.7. On the other hand, the overlap between *seurat* and *brennecke* is one of the highest across datasets (ranging from 50 to 70%), yet the differences between their corresponding NMI scores are 0.45.

triku selects genes that are biologically relevant

Based on these results, we studied the biological relevance of the genes selected by different FS methods in three alternative ways.

Genes whose expression, or lack thereof, is limited to a single population are more likely to be cell-type specific and thus might be better candidates as positive or negative cell population markers. Therefore, we studied which are the best FS methods to select genes showing a localized expression pattern.

Mitochondrial and ribosomal genes are usually highly expressed and many FS methods tend to overselect them despite them not being particularly relevant in most single-cell studies. In fact, they are commonly excluded from downstream analysis [21, 24, 25]. Assuming that in these benchmarking datasets ribosomal and mitochondrial genes are not as relevant to the biology of the dataset, we measured the percentage of these genes in the subset of genes selected by *triku* and compared it to other FS methods.

Lastly, we analyzed the biological pertinence of the selected genes by performing Gene Ontology Enrichment Analysis (GOEA) on a dataset of immune cell populations whose underlying biology is well understood, as a robust indicator of FS quality; and analyzed UMAP visual quality of different samples to assert that cell subtypes were independently represented in the UMAP.

Selection of locally-expressed genes

We first studied the expression pattern of genes selected by *triku* and other methods, as shown in Figure S4. We observed that out of the 9 populations of the artificial dataset, when a gene is selected by *triku*–exclusively or together with other FS methods–, one of the populations had a markedly higher or lower expression compared to the rest. On the other hand, when a gene is selected by other FS methods and not by *triku*, we do not observe any population-specific expression pattern. For instance, genes exclusively selected by *scanpy* had a wide expression variation across clusters, but they were not exclusive of one or two clusters. Features selected by *std* and *scry* showed some variation, but it was overshadowed by the high expression of the gene, and therefore not relevant under the previous premise.

To evaluate the cluster expression of selected genes in benchmarking datasets, for each gene we scaled its expression to the 0-1 range, and sorted the clusters so that the first one had the greatest expression. Figure S5 shows the expression patterns for several benchmarking datasets. We see that, in most datasets, *triku* showed more biased expression patterns, that is, genes selected by *triku* were expressed, on average, on fewer clusters than the genes selected by other FS methods. The following best methods were *scanpy*, *seurat*, *sct* and *bren-*

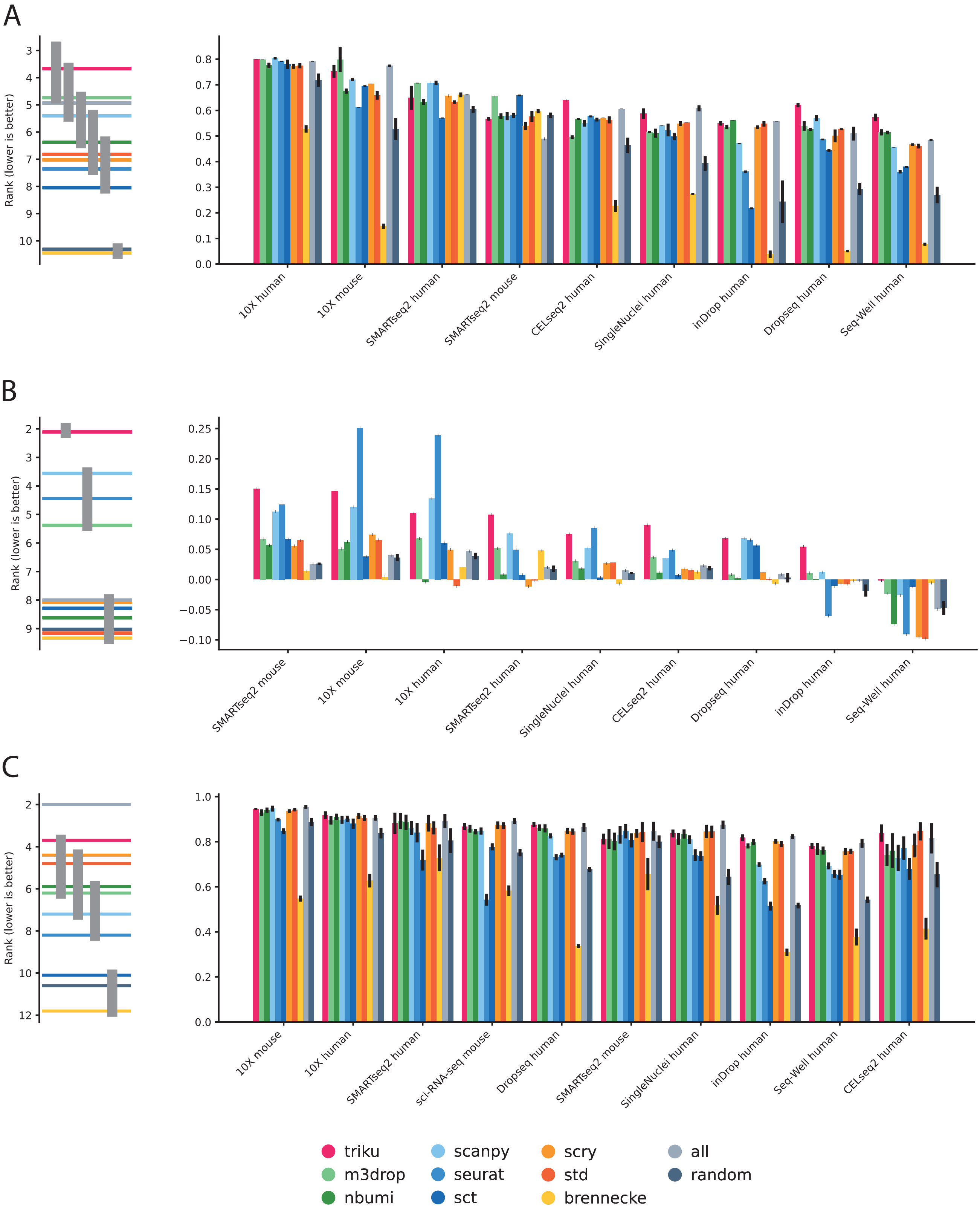

Figure 4. NMI, silhouette of annotated cell types and decision tree metrics in Ding datasets. Barplots of the three represented metrics. Each barplot represents the mean over 5 runs, and the vertical bar is the standard deviation. (A) NMI between clustering solutions and annotated cell types. (B) Silhouette coefficient of annotated cell types. (C) Decision tree classifier accuracy using a 10-fold cross validation of annotated cell types. The plot on the left is a critical difference diagram, where each horizontal bar represents the mean rank for all datasets. If two or more bars are linked by a vertical bar, the mean ranks for those FS methods are not significantly different (Quade test, $\alpha = 0.05$).

necke, with similar or slightly less biased expression patterns as compared to *triku*. With these methods, up to 80% of the expression of the gene was usually restricted to the 2 to 3 clusters that most expressed it.

m3drop and *nbumi* performed similarly, and showed an expression distribution across clusters similar to a random selec-

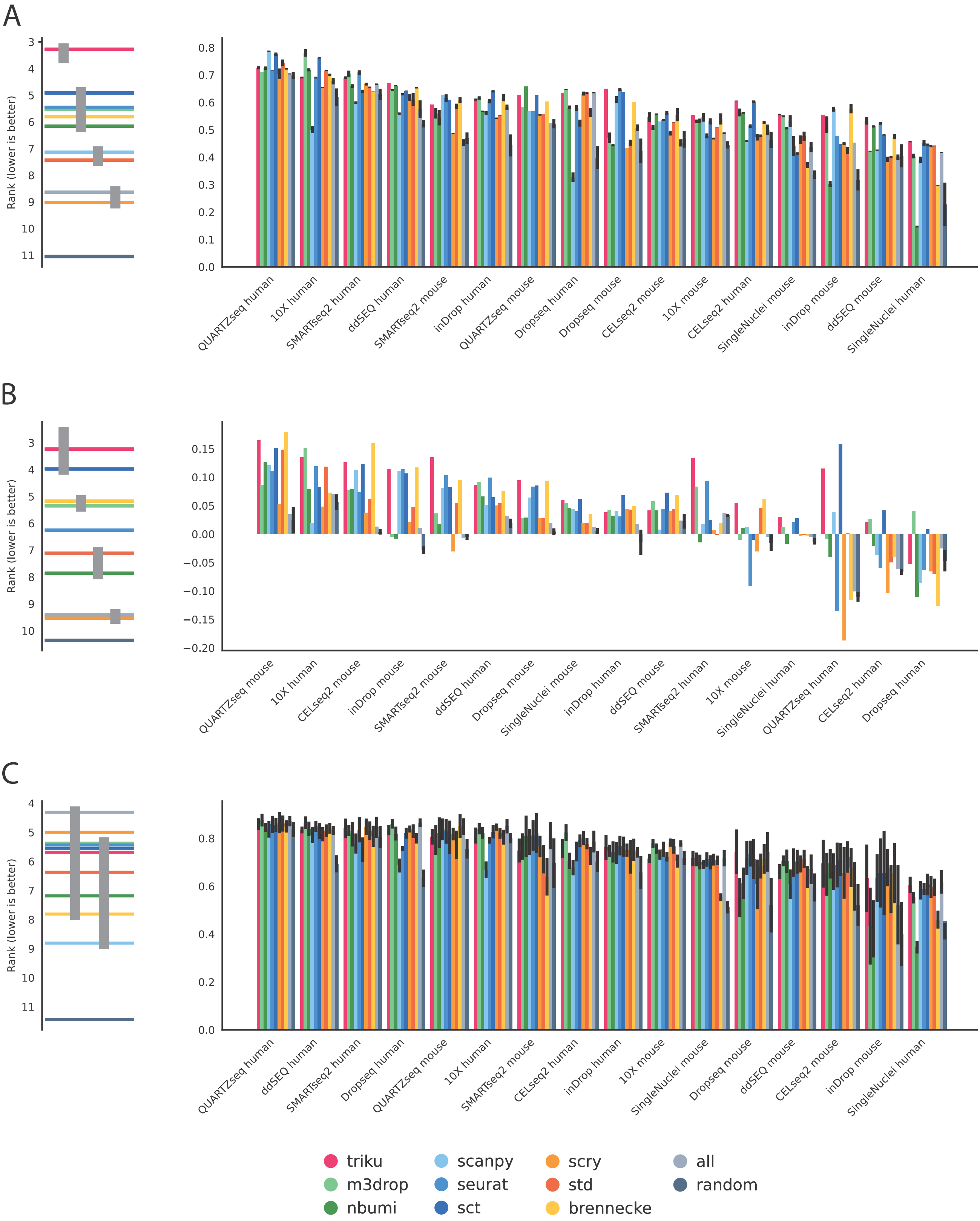

Figure 5. NMI, silhouette of annotated cell types and decision tree metrics in Mereu datasets. Barplots of the three represented metrics. Each barplot represents the mean over 5 runs, and the vertical bar is the standard deviation. (A) NMI between clustering solutions and annotated cell types. (B) Silhouette coefficient of annotated cell types. (C) Decision tree classifier accuracy using a 10-fold cross validation of annotated cell types. The plot on the left is a critical difference diagram, where each horizontal bar represents the mean rank for all datasets. If two or more bars are linked by a vertical bar, the mean ranks for those FS methods are not significantly different (Quade test, α = 0.05).

tion of genes, which was slightly biased towards 3 to 5 clusters accumulating up to 80 % of the expression of the gene. Lastly, *std* and *scry* methods were the least biased, and showed almost a linear decrease of expression percentage across clusters, with 4 to 6 clusters accumulating up to 80 % of the expression of the gene.

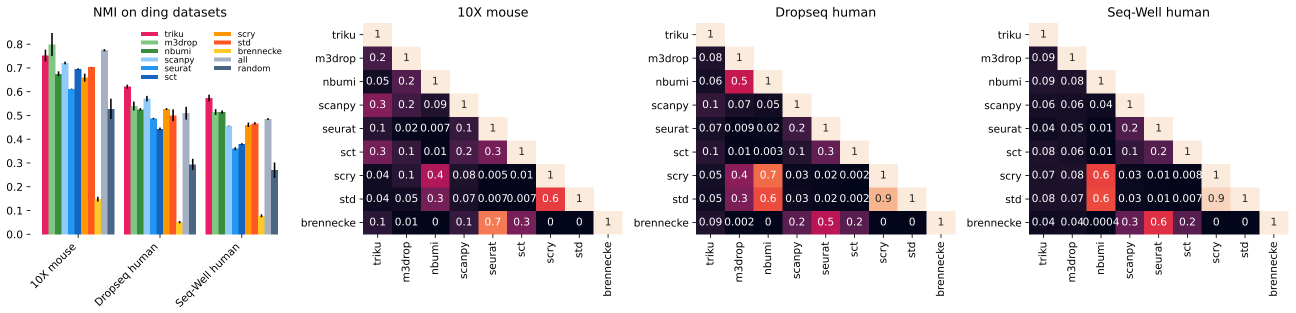

Figure 6. Heatmaps of overlap of features between pairs of methods. Barplot on the left represents the NMI values for the three selected datasets on the overlap heatmaps on the right. For each pair of methods, the value represents the proportion of features that are shared between the two methods. The number of genes selected in each method is the automatic cutoff by *triku*.

Avoidance of mitochondrial and ribosomal genes

Table 1 shows the percentage of genes that code for ribosomal and mitochondrial proteins within the genes selected by different FS methods in the two sets of benchmarking datasets. We observed that *std* and *scry*, followed by *m3drop*, were the only methods that tended to overselect mitochondrial and ribosomal genes. Among the rest of the methods, *triku* showed percentages that were comparable to the rest of the methods, and slightly lower for the Ding datasets.

Table 1. Percentage of ribosomal protein (RBP) and mitochondrial (MT) genes appearing within the selected genes by each FS method.

	Mereu		Ding	
	**% RBP**	**% MT**	**% RBP**	**% MT**
**triku**	1.92	0.08	0.12	0.01
**m3drop**	3.83	0.43	0.69	0.11
**nbumi**	1.87	0.16	0.44	0.09
**scanpy**	1.56	0.09	0.27	0.04
**seurat**	1.96	0.20	0.25	0.01
**sct**	1.34	0.18	0.04	0.03
**scry**	4.20	0.61	1.31	0.27
**std**	5.04	0.58	2.00	0.33
**brennecke**	1.01	0.09	0.03	0.01

Selection of genes based on gene ontologies

We assessed the quality of the GO output by studying its term composition. We selected two PBMC datasets from the Ding datasets. We used PBMC datasets for this analysis because their cell-to-cell variability has been extensively studied using single-cell technologies as Fluorescence Activated Cell Sorting (FACS) and scRNA-seq [26, 27, 28, 29, 30]. Using these datasets, we measured the proportion of GO terms obtained in the output that were tightly related to the biological system under study.

Figures 7 and S6 show the first 25 GO terms obtained with the genes selected by each FS method on the two PBMC datasets–10X human and Dropseq human–, where the terms tightly related to immune processes–chosen by three independent assessors–have been highlighted. We observed that *triku* was the FS method that yielded the most terms directly related to immune processes, with 25/25 + 15/25 = 40/50 related terms in the Ding Dropseq and 10X datasets, respectively. Examples of terms that we considered to be tightly related to immune processes included *B cell receptor signalling pathway*, *neutrophil degranulation* and *regulation of T cell proliferation*. The next methods were *scanpy* and *m3drop*, whose performances were comparable or better to that of *triku* for the 10X dataset–21/25 and 15/25–but less robust for the Dropseq dataset–10/25 and 9/25 related terms–, summing up to a total of 33/50 and 24/50. The rest of the FS methods mainly selected genes that were related to general cell functions such as RNA processing, protein processing and cell-cycle regulation.

Cell subtype distribution on UMAP

UMAP and clustering are common steps within single-cell pipelines. To assess the quality of FS on the UMAP representation, we analyzed whether different cell types appeared as different entities in the UMAPs. In other words, if two cell types–having different transcriptional profiles–appear mixed within the UMAP, it is possible that some of the features from the transcriptional profile of the cell types are not selected as relevant.

We analyzed two Ding human PBMC datasets–CELseq2 and Seq-Well–, as shown in Figure 8. In general, we observe a high mixture of cell types within *brennecke* and *random* FS throughout datasets, where major cell types were mixed and, therefore, would be highly uninformative for cell type characterisation.

From the CELseq2 dataset we observe that the mucosal-associated invariant T cell population is more diffuse in *m3drop*, *seurat*, *sctransform* and *all*; and $\gamma\delta$ T cells showed a higher mixture degree in *scanpy*, *seurat*, *sctransform*. Additionally, the dendritic cell population, which appeared near CD14^{+} monocytes, appears less separated in *std* and *all*, and is mixed with CD16^{+} monocytes in *seurat*. Finally, memory and resting naïve B cells appear less separated in *m3drop*, *nbumi*, *scanpy*, *seurat*, and *sct*.

Regarding the Seq-Well dataset we observe that mucosal-associated invariant T cells are less defined in *triku*, *scanpy*, *m3drop*, *seurat* and *sctransform*; and *seurat* and *sctransform* show a high degree of mixture of major T cell populations (CD4 and CD8). Additionally, resting naïve B cells are mixed with proliferative naïve B cells in *scanpy*, *seurat*, and *sct*; and with CD4^{+} T cells in *scry* and *std*.

Therefore from these results we conclude that using *triku* as FS method produces well-defined cell type populations and subpopulations. Interestingly, using no FS method also yields good visual results, even better than other FS methods, probably because PCA takes in consideration all the information from the genes, and the PCA projection automatically excludes nonrepresentative features. Nevertheless, this effect should be addressed with other datasets and other steps in the analysis pipeline.

Discussion

FS methods are a key step in any scRNA-seq sequencing analysis pipeline as they help us obtain a dimensionally reduced version of the dataset that captures the most relevant information and eases the interpretation and understanding of its

underlying biology. However, every FS method relies on a set of assumptions regarding what characteristics make a gene relevant. FS methods that sort genes according to their dispersion assume that gene expression variability is indicative of its biological relevance. FS methods like *nbumi* and *m3drop* assume that genes showing a proportion of zero-counts that is greater than expected (according to a null distribution) are more likely to be informative. *Triku* assumes that genes that have a localized expression in a subset of cells that share an overall transcriptomic similarity are more likely to define cell types. A general trend in FS method design has been to refine the requirements that a gene must meet in order for it to be selected, from the more general dispersion-based to more sophisticated formulations. It is noteworthy that the requirements in *triku* are consistent with the previous dispersion-based and zero-count-based formulations, but involve a new aspect that we consider essential for an accurate gene selection: a localized expression in neighboring cells. Another important advantage of *triku* over FS methods that consider the zero-count distribution is that, unlike *m3drop* and *nbumi*, *triku* does not assume gene counts to follow any particular distribution, since it estimates the null distribution from the dataset, thus extending the range of single-cell technologies that it can use beyond droplet-based technologies.

We verified the locality of the genes selected by *triku* in different artificial and real scRNA-seq datasests and concluded that, on average, the expression of *triku*-selected genes is restricted to fewer, well-defined clusters. In addition, the clusters obtained when using *triku*-selected genes as input for unsupervised clustering in both artificially generated and biological datasets have a better resolved pattern structure, as shown by their increased Silhouette coefficients. In the case of artificial datasets, where the degree of mixture between clusters can be predefined, *triku* proved to be able to recover the originally-defined cell populations. In fact, we found that the higher the degree of mixture between clusters, the more obvious the advantage of *triku* over the rest of the FS methods tested.

An important difficulty in the interpretation of single-cell data is that we must consider that cell-to-cell variability has both technical and biological components. I.e., it is difficult to know whether a set of genes is differentially expressed between cell clusters due to technical reasons (differences in the efficiency of mRNA capture, amplification and sequencing) or if it constitutes a biological signal. Moreover, there is a wide range of sources of biological variability within a dataset, some of which might not be of interest depending on the experimental context. For instance, fluctuations in genes that regulate the cell cycle constitute a source of biological variability that is often disregarded. This has been extensively studied and addressed in a number of ways: normalization, regression of unwanted sources of variation, etc. [12, 31, 32, 33].

The expression of genes whose variability is associated with technical reasons tend to have a high dispersion but their expression is usually not restricted to a few clusters. A good example of these genes are the ribosomal and mitochondrial genes, which are expressed across all cell types at different levels. Our results show that these genes are in fact selected by the majority of compared FS methods due to their high expression and cell-to-cell variability, but are less likely to be selected by *triku*, since they do not usually meet the locality requirement. Additionally, when performing GOEA, we observed that the list of genes obtained with *triku* were more enriched for terms that are specifically related to a biological process of the system under study.

In our work, we have observed that the genes selected by different FS methods might show little overlap between them. This phenomenon has been described elsewhere [34]. In fact, gene covariation and redundancy is a well characterized effect that has been observed in omics studies. The effect of redundancy arises from the fact that different cell types must have a common large set of pathways to be active. The difference between cell type and cell state is that two cell types might have large sets of pathways that are different between each other, and two cell states will only differ in a few pathways. Since pathways are composed of many genes, only choosing a reduced set of genes from a set of pathways from cell type A and B might be enough to differentiate them, and we might not need to select all genes from all pathways. This "paradigm" explains several effects. Qiu et al. described that scRNA-seq datasets could preserve basic structure after gene expression binarization [35] or by conducting very shallow sequencing experiments [4]. This can be explained by the fact that only a few genes are necessary to describe the main cell populations in a single-cell dataset, and the presence/absence of a certain marker is often more informative than its expression level. This is related to the notion that despite the high dimensionality of omics studies, most biological systems can be explained in a reduced number of dimensions. Moreover, some authors have claimed this low dimensionality to be a natural and fundamental property of gene expression data [4]. This highlights the importance of designing accurate FS methods that extract the fundamental information from single-cell datasets.

Triku Python package is available at https://github.com/alexmascension/triku and can be downloaded using PyPI. *Triku* has been designed to be compatible with *scanpy* syntax, so that *scanpy* users can easily include *triku* into their pipelines. Notebooks developed for figure production and additional results are located in https://github.com/alexmascension/triku_notebooks and https://doi.org/10.5281/zenodo.4016714

Methods

The *triku* workflow is further described in Supplementary Methods.

Artificial and benchmarking datasets

In order to perform the evaluation of the FS methods we used a set of artificial and biological benchmarking datasets. Artificial datasets were constructed using *splatter* R package (v 1.10.1). Each dataset contains 10,000 cells and 15,000 genes, and consists of 9 populations with abundances in the dataset of {25%, 20%, 15%, 10%, 10%, 7%, 5.5%, 4%, 3.5%} of the cells. Each dataset contains a parameter, `de.prob`, that controls the probability that a gene is differentially expressed. Lower `de.prob` values (< 0.05) imply that different populations have fewer differentially expressed genes between them and, therefore, are more difficult to be differentiated. Selected values of `de.prob` are {0.0065, 0.008, 0.01, 0.016, 0.025, 0.05, 0.1, 0.3}. Populations in datasets with `de.prob` values above 0.05 are completely separated in the low-dimensionality representation with UMAP, even without feature selection (Figure S7).

Regarding biological datasets, two benchmarking datasets have been recently published by Mereu et al. [20] and Ding et al. [19]. The aim of these two works is to analyze the diversity of library preparation methods, e.g. 10X, SMART-seq2, CEL-seq2, single nucleus or inDrop. Mereu et al. use mouse colon cells and human PBMCs to perform the benchmarking, whereas Ding et al. use mouse cortex and human PBMCs. There are a total of 14 datasets in Mereu et al. and 9 in Ding et al. An additional characteristic of these datasets is that they have been manually annotated, and this annotation is useful as a *semi* ground truth. Ding dataset files were downloaded from Single Cell Portal (accession numbers SCP424 and SCP425),

and cell type metadata is located within the downloaded files. Mereu datasets were downloaded from GEO database (accession GSE133549), and cell type metadata was provided by the authors after personal request.

FS methods

Triku is compared to the following FS methods:

- *m3drop* [17] [distribution-based]: fits a Michaelis-Menten equation to the percentage of zeros versus μ, and selects features with higher percentages of zeros than expected. The features are selected with the *M3DropFeatureSelection* function from `M3Drop` R package.
- *nbumi* [distribution-based]: it acts in the same manner as *m3drop*, but fitting a negative binomial equation instead of a Michaelis-Menten equation. The features are selected with the *NBumiFeatureSelectionCombinedDrop* function.
- *scanpy* [11] [dispersion-based]: selects features based on a z-scored deviation, adapted from *Seurat*'s method. The features are selected with the *sc.pp.highly_variable_genes* function from *scanpy* (v 1.6.0).
- *seurat* [36] [dispersion-based]: *FindVariableFeatures* function, which fits a line to the relationship of log(variance) and log(mean) using local polynomial regression (loess). Then standardizes the feature values using the observed mean and expected variance.
- *sctransform (sct)* [12] [dispersion-based]: Seurat implementation of *SCTransform* function applies *NormalizeData*, *ScaleData*, and *FindVariableFeatures* with *vst* mode (default in Seurat).
- *scry* [13] [dispersion-based]: computes a deviance statistic for counts based on a multinomial model that assumes each feature has a constant rate. The features are selected with the *devianceFeatureSelection* function from `scry` R package (v 0.99.0).
- Standard deviation (*std*) [dispersion-based]: Computed directly using Numpy (v 1.18.3).
- *brennecke* [8] [dispersion-based]: fits a curve based on the square of the coefficient of variation (CV^2) versus the mean expression (μ) of each gene and selects the features with higher CV^2 and μ. The features are selected with the *BrenneckeGetVariableGenes* function from `M3Drop` R package (v 1.12.0).

FS and dataset preprocessing

To make the comparison between FS methods, each feature is ranked based on the score provided by each FS method. Calculating the ranking instead of just selecting the features allow us to select different numbers of features when needed. By default, the number of features is the one automatically selected by *triku*. Additionally, in some contexts, analyses are performed with all features or with a random selection of features.

After the ranking of genes is computed, dataset processing is performed equally for all methods, in artificial and benchmarking datasets. Datasets are first log transformed –if required by the method–, and PCA with 30 components is calculated. Then, the k-Nearest Neighbors (kNN) matrix is computed setting k as $\sqrt{n_{cells}}$. Uniform Manifold Approximation and Projection (UMAP) (v 0.3.10) is then applied to reduce the dimensionality for plotting. If community detection is required, leiden (v 0.7.0) is applied selecting the resolution that matches the number of cell types manually annotated in the dataset. This procedure is repeated with 10 different seeds. This conditions the output of *triku*, random FS, PCA projection, neighbor graph, leiden community detection, and UMAP.

ARI and NMI calculation in artificial and benchmarking datasets

In order to compare the leiden community detection results with the ground-truth labels from artificial and biological datasets, we used the Adjusted Rand Index (ARI) and the Normalized Mutual Information (NMI) scores [37].

The ARI is a revision of the RI, with correction of the expected RI:

$$ARI = \frac{RI - RI_{Expected}}{RI_{max} - RI_{Expected}}$$

If T and L are the labels of the cell types (true populations) and leiden communities respectively, the NMI between T and L is:

$$NMI(T, L) = \frac{2I(T; L)}{H(T) + H(L)}$$

Where $H(X)$ is the entropy of the labels, and $I(T; L)$ is the mutual information between the two sets of labels. This value is further described in [38]. We used scikit-learn (v 0.23.1) implementation of NMI, *sklearn.metrics.adjusted_mutual_info_score*.

One of the advantages of NMI against other mutual information methods is that it performs better with label sets with class imbalance, which are common in single-cell datasets, where there are differences in the abundance of cell types.

On artificial datasets, leiden was applied using the first 250 and 500 selected features, and the resulting community labels were compared with the population labels from the dataset. On benchmarking datasets, leiden was applied with the manually-curated cell types.

Silhouette coefficient in benchmarking datasets

In order to assess the clustering performance of the communities obtained with benchmarking datasets we used the Silhouette coefficient. The Silhouette coefficient compares the distances of the cells within each cluster (intra-cluster) and between clusters (inter-cluster) within a measurable space. The distance between two cells is the cosine distance between their gene expression vectors, considering only the genes selected by each FS method. The greater the distance between cells that belong to different clusters and the smaller the distance between cells from different cluster, the greater the Silhouette score.

In order to calculate the Silhouette coefficient for a cell c within cluster C_i (out of n clusters), the mean distance between the cell and the rest of the cells within the cluster is computed using the gene expression:

$$a(c) = \frac{1}{|C_i| - 1} \sum_{j \in C_i, c \neq j} d(c, j)$$

Then, the minimum mean distance between that cell and the rest of cells from other clusters is computed:

$$b(c) = \min_{C_k \neq C_i} \left\{ \frac{1}{C_k} \sum_{j \in C_k} d(i, j) \right\} \quad k \in 1, \cdots, n$$

Then the Silhouette coefficient is computed as

$$s(c) = \frac{b(c) - a(c)}{\max b(c), a(c)}$$

Higher Silhouette scores imply a better separation between clusters and, therefore, a better performance of the FS method. We used scikit-learn implementation of Silhouette, *sklearn.metrics.silhouette_score*.

Effect of FS on supervised classification

Two different supervised classifiers (decision tree and KNN classifier from `scikit-learn`) were trained on the Ding and Mereu benchmarking datasets. Their performance on the different feature selected datasets was measured by computing the 10-fold cross validation score. The same feature numbers as in the NMI and ARI analysis were used to train the classifiers.

Overlap between gene lists

In order to calculate the overlap between selected features for each FS method, we applied the Jaccard index [39]: $jaccard(\mathbf{i},\mathbf{j}) = \frac{|\mathbf{i} \cap \mathbf{j}|}{|\mathbf{i} \cup \mathbf{j}|}$, where $\mathbf{i},\mathbf{j}$ are the sets of genes selected by the two FS methods.

Performance of gene selection and locality measures

In order to assess the performance of different FS methods selecting genes that are relevant for the dataset, we applied two different strategies for artificial and biological datasets.

For artificial datasets, we selected 4 representative genes of each of the combinations of genes shown in Figure S4. Then we calculated the mean expression of each of the for genes in each population, and we represent this information in the barplots.

For benchmarking datasets, in order to represent the Figure S5, for each dataset and FS method we used the following procedure: for each gene, the expression was scaled to sum 1 across all cells. Then, leiden clustering was run with resolution parameter value 1.2. For each cluster, the proportion of the expression was calculated, and the clusters were ordered so that the first cluster is the one that concentrates the majority of the expression. To create Figure S5, the average value of the proportion of expression is calculated.

Proportion of ribosomal and mitochondrial genes

When calculating the proportion of mitochondrial and ribosomal genes, the list of existing ribosomal and mitochondrial proteins was calculated by extracting the genes starting with *RPS*, *RPL* or *MT-*. The proportion of mitochondrial or ribosomal genes is the quotient between the genes of the previous list that appear selected by that FS method, and the genes in the list.

GO enrichment analysis

In order to calculate the sets of gene ontologies enriched for the selected features of each FS method, we used python *gseapy* (v 0.9.17) module *gseapy.enrichr* function with the list of the first 1000 selected features against the *GO_Biological_Process_2018* ontology. From the list of enriched ontologies, the 25 with the smallest adjusted p-value were selected.

Ranking and CD

During calculation of NMI and Silhouette coefficients, to evaluate the overall performance of the FS methods across different datasets, the FS methods are ranked, where 1 is the best rank. The methodology proposed by Demšar [40] is used to test for significant differences among FS methods in the datasets: The Friedman rank test is applied to test whether the mean rank values for all FS methods are similar (null hypothesis). If the Friedman rank test rejects the null hypothesis ($\alpha < 0.05$), this implies a statistically significant difference among at least two FS methods. If the null hypothesis is refuted we apply the Quade post-hoc test between all pairs of FS methods to check which pairs of FS methods are significantly different ($\alpha < 0.05$). These results are then plotted in a critical difference diagram.

Cell subtype quality representation in UMAP

For this analysis we are going to use 2 Ding human PBMC datasets–CELseq2 and Seq-Well–. To make the analysis more precise, we subdivided some of the cell types designed in the datasets into different subtypes, based on relevant markers from bibliography, which were also robust across datasets [41, 42, 43, 44].

The cell type division into cell subtypes was the following: B cells were divided into resting naïve B cells (*IGHD*$^+$*TCL1A*$^+$*CD79A*$^+$), proliferative naïve B cells (*IGHD*$^+$*CD69*$^+$*CCR7*$^+$), memory B cells (*IGHG1*$^+$*CD79A*$^+$*CD27*$^+$), and plasma cells (*MZB1*$^+$*JCHAIN*$^+$*IGHA2*$^+$); *CD14*$^+$ monocytes were divided into *CD14*$^+$ resting monocytes (*CIITA*$^+$*CLEC12A*$^+$*NAIP*$^+$) and *CD14*$^+$ active monocytes (*EGR1*$^+$*IFITM3*$^+$*IER2*$^+$); *CD4*$^+$ and *CD8*$^+$ T cells were subdivided into two additional cell types, $\gamma\delta$ T cells (*TRDC*$^+$*KLRC1*$^+$*A2M-AS1*$^+$) and Mucosal-associated invariant T cells (*SLC4A10*$^+$*DPP4*$^+$)

To assign these new cell subtypes we used the population matching algorithm described below. The unsupervised populations used to match the cell subtypes based on their markers were leiden clusters produced with a high resolution value (8 for both datasets), so that several clusters can be assigned to the same subtype.

To create the UMAPs and leiden clusters, once features were selected, the neighbour graph of the cells was computed. This graph is then used to compute both the UMAP and leiden clusters.

Population matching algorithm

The aim of this algorithm is to assign a set of clusters to a set of labels, where each label contains a list of representative markers. For each label we extract the matrix of counts of the genes belonging to the label. Then, we create a new matrix, where we assign to each cell and gene the sum of the counts of the gene within its kNN, divided by the number of neighbors. This steps reduces the noisiness of the expression, and also exacerbates the local expression of a gene and dampens the expression of sparse genes.

Gene expression values are substituted by the ranked index of their expression; and the values are divided by the largest index to sum 1. Therefore, the cell with the highest expression will have a value of 1 for that gene, while the lowest expressed cell will have a value of near 0. After this normalization is applied to the rest of genes within the label, the mean of the normalized values across genes is computed, so that each cell has one value for that label.

After the previous steps are computed for the rest of labels, a new matrix with the number of clusters by the number of labels is computed. For each label and each cluster, the percentile of the normalized values within cells of that cluster is computed (percentile 70 by default). This helps reduce noise on normalized values, and assign a unique number per cluster.

This algorithm allows to choose intermediate states, that is, cell labels with a high similarity. By default, the label with the highest score per cluster is chosen. With the intermediate state option, labels that have a similar value as the label with the highest value are included. The difference in values is set as a threshold (0.05 by default), and labels with difference in value greater than the threshold are not merged.

This algorithm can be found in the following GitHub repository: `https://github.com/alexmascension/cell_asign`, and can be installed via PyPI as `pip install cellassign`.

Availability of supporting data and materials

Project name: Triku

Project home page: https://www.github.com/alexmascension/triku

Notebook repository: https://github.com/alexmascension/triku_notebooks
Notebook output repository: https://doi.org/10.5281/zenodo.4016714
Operating system(s): Platform independent
Programming language: Python
License: BSD 3
RRID:SCR_020977

Declarations

List of abbreviations

Single-cell RNA sequencing: scRNA-seq; Feature Selection: FS; Feature Extraction: FE, Principal Component Analysis: PCA, Negative Binomial: NB, Normalized Mutual Information (NMI); Fluorescence Activated Cell Sorting: FACS; Gene Ontology: GO; Gene Ontology Enrichment Analysis: GOEA; Peripheral Blood Mononuclear Cells: PBMC; Uniform Manifold Approximation and Projection: UMAP; k-Nearest Neighbors: kNN.

Consent for publication

Not applicable.

Competing Interests

The authors declare that they have no competing interests.

Funding

This work was supported by grants from Instituto de Salud Carlos III (AC17/00012 and PI19/01621), cofunded by the European Union (European Regional Development Fund/ European Science Foundation, Investing in your future) and the 4D-HEALING project (ERA-Net program EracoSysMed, JTC-2 2017); Diputación Foral de Gipuzkoa, and the Department of Economic Development and Infrastructures of the Basque Government (KK-2019/00006, KK-2019/00093); European Union FET project Circular Vision (H2020-FETOPEN, Project 899417), Ministry of Science and Innovation of Spain; and MICINN/AEI/FEDER, UE (PID2020-119715GB-I00) co-funded by the European Regional Development Fund (ERDF/ESF, Investing in your future). AMA was supported by a Basque Government Postgraduate Diploma fellowship (PRE_2020_2_0081), and OIS was supported by a Postgraduate Diploma fellowship from la Caixa Foundation (identification document 100010434; code LCF/BQ/IN18/11660065).

Author's Contributions

Conceptualization: AMA; Funding Acquisition: MJA-B, AMA, OI-S; Investigation: AMA, OI-S, MJA-B, AI; Methodology: AMA, OI-S, II; Project Administration: AI, MJA-B; Resources: MJA-B; Software: AMA, OI-S; Supervision: II, AI, MJA-B; Visualization: AMA, OI-S; Writing - Original Draft Preparation: AMA, OI-S; Writing - Review and Editing: AMA, OI-S, II, MJA-B, AI.

Acknowledgements

We would like to thank Amaia Elícegui, Ainhoa Irastorza and Paula Vázquez for the assessment of the immune Gene Ontology terms.

References

1. Trapnell C. Defining cell types and states with single-cell genomics. Genome Research 2015;25(10):1491–1498. https://doi.org/10.1101/gr.190595.115.
2. Maclean AL, Hong T, Nie Q. Exploring intermediate cell states through the lens of single cells. Current Opinion in Systems Biology 2018;9:32–41. https://doi.org/10.1016/j.coisb.2018.02.009.
3. Bzdok D, Altman N, Krzywinski M. Statistics versus machine learning. Nature Methods 2018;15(4):233–234. https://doi.org/10.1038/nmeth.4642.
4. Heimberg G, Bhatnagar R, El-Samad H, Thomson M. Low Dimensionality in Gene Expression Data Enables the Accurate Extraction of Transcriptional Programs from Shallow Sequencing. Cell Systems 2016;2(4):239–250. https://doi.org/10.1016/j.cels.2016.04.001.
5. Saeys Y, Inza I, Larrañaga P. A review of feature selection techniques in bioinformatics. Bioinformatics 2007;23(19):2507–2517. https://doi.org/10.1093/bioinformatics/btm344.
6. Luecken MD, Theis FJ. Current best practices in single-cell RNA-seq analysis: a tutorial. Molecular Systems Biology 2019;15(6). https://doi.org/10.15252/msb.20188746.
7. Su K, Yu T, Wu H. Accurate feature selection improves Single-cell RNA-seq Cell clustering. Briefings in Bioinformatics 2021;22(5). https://doi.org/10.1093/bib/bbab034.
8. Brennecke P, Anders S, Kim JK, Kołodziejczyk AA, Zhang X, Proserpio V, et al. Accounting for technical noise in single-cell RNA-seq experiments. Nature Methods 2013;10(11):1093–1095. https://doi.org/10.1038/nmeth.2645.
9. Osorio D, Yu X, Zhong Y, Li G, Serpedin E, Huang JZ, et al. Single-cell expression variability implies cell function. Cells 2019;9(1):14. https://doi.org/10.3390/cells9010014.
10. Stuart T, Butler A, Hoffman P, Hafemeister C, Papalexi E, Mauck WM, et al. Comprehensive Integration of Single-Cell Data. Cell 2019;177(7):1888–1902. https://doi.org/10.1016/j.cell.2019.05.031.
11. Wolf FA, Angerer P, Theis FJ. SCANPY: large-scale single-cell gene expression data analysis. Genome Biology 2018;19(1). https://doi.org/10.1186/s13059-017-1382-0.
12. Hafemeister C, Satija R. Normalization and variance stabilization of single-cell RNA-seq data using regularized negative binomial regression. Genome Biology 2019;20(1). https://doi.org/10.1186/s13059-019-1874-1.
13. Townes FW, Hicks SC, Aryee MJ, Irizarry RA. Feature selection and dimension reduction for single-cell RNA-Seq based on a multinomial model. Genome Biology 2019;20(1). https://doi.org/10.1186/s13059-019-1861-6.
14. Vieth B, Ziegenhain C, Parekh S, Enard W, Hellmann I. powsimR: Power analysis for bulk and single-cell RNA-seq experiments. Bioinformatics 2017;33(21):3486–3488. https://doi.org/10.1093/bioinformatics/btx435.
15. Chen W, Li Y, Easton J, Finkelstein D, Wu G, Chen X. UMI-count modeling and differential expression analysis FOR single-cell RNA sequencing. Genome Biology 2018;19(1). https://doi.org/10.1186/s13059-018-1438-9.
16. Svensson V. Droplet scRNA-seq is not zero-inflated. Nature Biotechnology 2020;38(2):147–150. https://doi.org/10.1038/s41587-019-0379-5.
17. Andrews TS, Hemberg M. M3Drop: dropout-based feature selection for scRNASeq. Bioinformatics 2018;35(16):2865–2867. https://doi.org/10.1093/bioinformatics/bty1044.
18. Zappi L, Phipson B, Oshlack A. Splatter: simulation of single-cell RNA sequencing data. Genome Biology 2017;18(174). https://doi.org/10.1186/s13059-017-1305-0.

19. Ding J, Adiconis X, Simmons SK, Kowalczyk MS, Hession CC, Marjanovic ND, et al. Systematic comparison of single-cell and single-nucleus RNA-sequencing methods. Nature Biotechnology 2020;38:737–746. https://doi.org/10.1038/s41587-020-0465-8.
20. Mereu E, Lafzi A, Moutinho C, Ziegenhain C, Mccarthy DJ, Álvarez Varela A, et al. Benchmarking single-cell RNA-sequencing protocols for cell atlas projects. Nature Biotechnology 2020;38:747–755. https://doi.org/10.1038/s41587-020-0469-4.
21. Freytag S, Tian L, Lönnstedt I, Ng M, Bahlo M. Comparison of clustering tools in R for medium-sized 10x Genomics single-cell RNA-sequencing data. F1000 Research 2018;7(1297). https://doi.org/10.12688/f1000research.15809.2.
22. Lall S, Ghosh A, Ray S, Bandyopadhyay S. sc-REnF: An Entropy Guided Robust Feature Selection for Single-Cell RNA-seq Data 2021;.
23. Hemphill E, Lindsay J, Lee C, Măndoiu I, Nelson C. Feature selection and classifier performance on diverse biological datasets. BMC Bioinformatics 2014;15. https://doi.org/10.1186/1471-2105-15-S13-S4.
24. Lun ATL, Mccarthy DJ, Marioni JC. A step-by-step workflow for low-level analysis of single-cell RNA-seq data with Bioconductor. F1000 Research 2016;5(2122). https://doi.org/10.12688/f1000research.9501.2.
25. Senabouth A, Lukowski SW, Hernandez JA, Andersen SB, Mei X, Nguyen QH, et al. ascend: R package for analysis of single-cell RNA-seq data. Gigascience 2019;8(8). https://doi.org/10.1093/gigascience/giz087.
26. Chen J, Cheung F, Shi R, Zhou H, Lu W. PBMC fixation and processing for Chromium single-cell RNA sequencing. Journal of Translational Medicine 2018;16(1). https://doi.org/10.1186/s12967-018-1578-4.
27. Massoni-Badosa R, Iacono G, Moutinho C, Kulis M, Palau N, Marchese D, et al. Sampling time-dependent artifacts in single-cell genomics studies. Genome Biology 2020;21(1). https://doi.org/10.1186/s13059-020-02032-0.
28. Villani AC, Satija R, Reynolds G, Sarkizova S, Shekhar K, Fletcher J, et al. Single-cell RNA-seq reveals new types of human blood dendritic cells, monocytes, and progenitors. Science 2017;356(6335). https://doi.org/10.1126/science.aah4573.
29. Zheng GXY, Terry JM, Belgrader P, Ryvkin P, Bent ZW, Wilson R, et al. Massively parallel digital transcriptional profiling of single cells. Nature Communications 2017;8(1). https://doi.org/10.1038/ncomms14049.
30. Zhu L, Yang P, Zhao Y, Zhuang Z, Wang Z, Song R, et al. Single-Cell Sequencing of Peripheral Mononuclear Cells Reveals Distinct Immune Response Landscapes of COVID-19 and Influenza Patients. Immunity 2020;53(3). https://doi.org/10.1016/j.immuni.2020.07.009.
31. Lytal N, Ran D, An L. Normalization Methods on Single-Cell RNA-seq Data: An Empirical Survey. Frontiers in Genetics 2020;11(41). https://doi.org/10.3389/fgene.2020.00041.
32. Nestorowa S, Hamey FK, Sala BP, Diamanti E, Shepherd M, Laurenti E, et al. A single-cell resolution map of mouse hematopoietic stem and progenitor cell differentiation. Blood 2016;128(8):e20–31. https://doi.org/10.1182/blood-2016-05-716480.
33. Tran HTN, Ang KS, Chevrier M, Zhang X, Lee NYS, Goh M, et al. A benchmark of batch-effect correction methods for single-cell RNA sequencing data. Genome Biology 2020;21(12). https://doi.org/10.1186/s13059-019-1850-9.
34. Yip SH, Sham PC, Wang J. Evaluation of tools for highly variable gene discovery from single-cell RNA-seq data. Briefings in Bioinformatics 2018;20(4):1583–1589. https://doi.org/10.1093/bib/bby011.
35. Qiu P. Embracing the dropouts in single-cell RNA-seq analysis. Nature Communications 2020;11(1169). https://doi.org/10.1038/s41467-020-14976-9.
36. Hao Y, Hao S, Andersen-Nissen E, III WMM, Zheng S, Butler A, et al. Integrated analysis of multimodal single-cell data. Cell 2021;https://doi.org/10.1016/j.cell.2021.04.048.
37. Kvalseth TO. On Normalized Mutual Information: Measure Derivations and Properties. Entropy 2017;19(11). https://doi.org/10.3390/e19110631.
38. Liu X, Cheng HM, Zhang ZY, Evaluation of Community Detection Methods; 2019.
39. Jaccard P. The distribution of the flora in the Alpine Zone. The New Phytologist 1912;11(2):37–50.
40. Demšar J. Statistical comparisons of classifiers over multiple data sets. Journal of Machine Learning Research 2006;7:1–30.
41. Sanz I, Wei C, Jenks SA, Cashman KS, Tipton C, Woodruff MC, et al. Challenges and opportunities for consistent classification of Human B cell and plasma cell populations. Frontiers in Immunology 2019;10. https://doi.org/10.3389/fimmu.2019.02458.
42. Shi J, Zhou J, Zhang X, Hu W, Zhao JF, Wang S, et al. Single-cell transcriptomic profiling of MAIT cells in patients with COVID-19. Frontiers in Immunology 2021;12. https://doi.org/10.3389/fimmu.2021.700152.
43. Stewart A, Ng JCF, Wallis G, Tsioligka V, Fraternali F, Dunn-Walters DK. Single-cell transcriptomic analyses define distinct peripheral b cell subsets and discrete development pathways. Frontiers in Immunology 2021;12. https://doi.org/10.3389/fimmu.2021.602539.
44. Wilk AJ, Rustagi A, Zhao NQ, Roque J, Martínez-Colón GJ, McKechnie JL, et al. A single-cell Atlas of the peripheral immune response in patients with Severe covid-19. Nature Medicine 2020;26(7):1070–1076. https://doi.org/10.1038/s41591-020-0944-y.

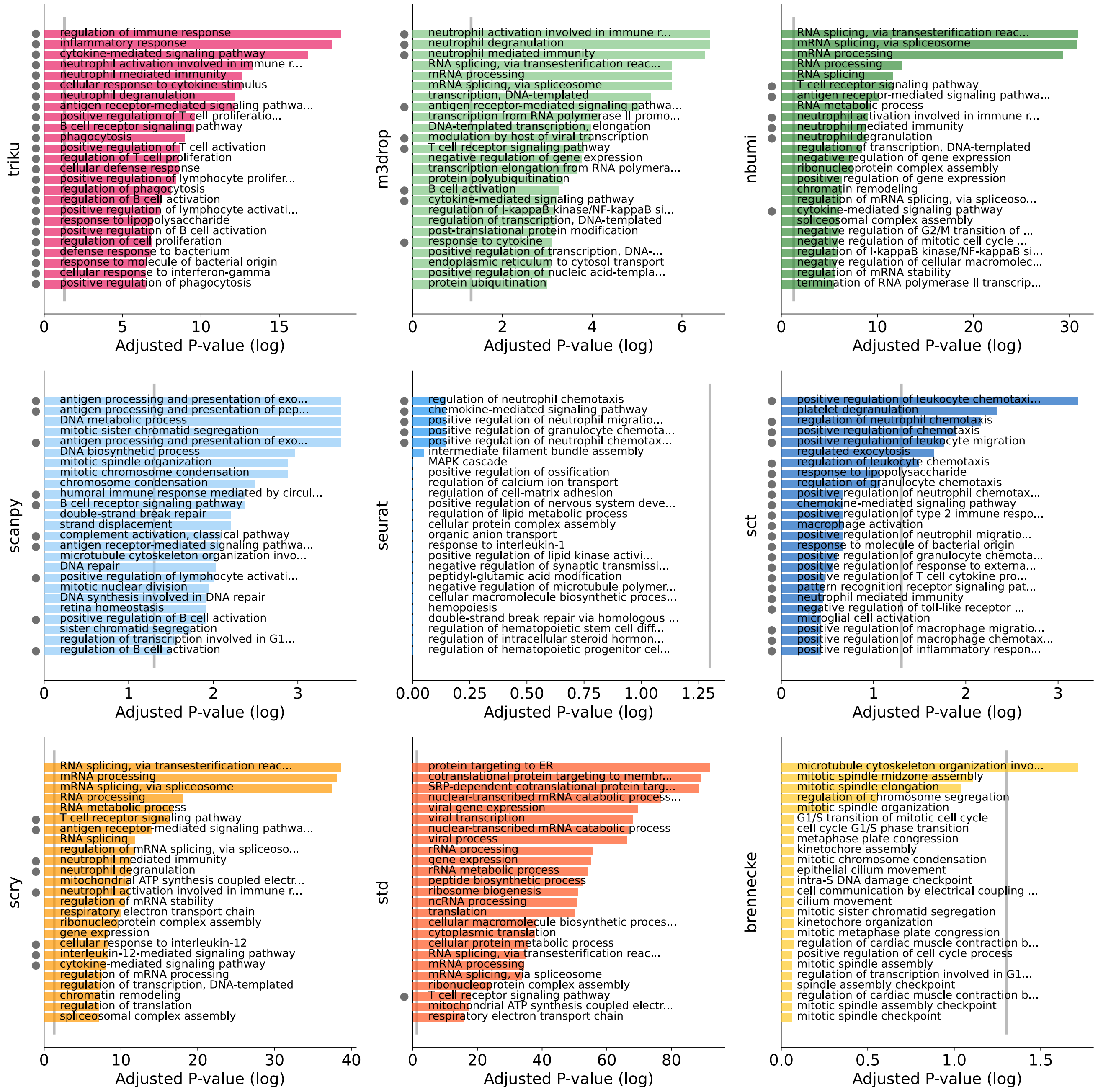

Figure 7. Barplot of p-values of GOEA. Each bin represents the number of features selected for each method, in Ding et al. human Dropseq dataset. The y value is the -log10 adjusted p-value for the best 25 ontologies. On the bottom, the bar plot shows the names of the ontology terms for the case with the best 1000 features. In immune datasets, gray dots at the left of each term represent that that term is directly-related to an immune process. Non-dotted terms refer to more general processes that may or may not be related to immune processes.

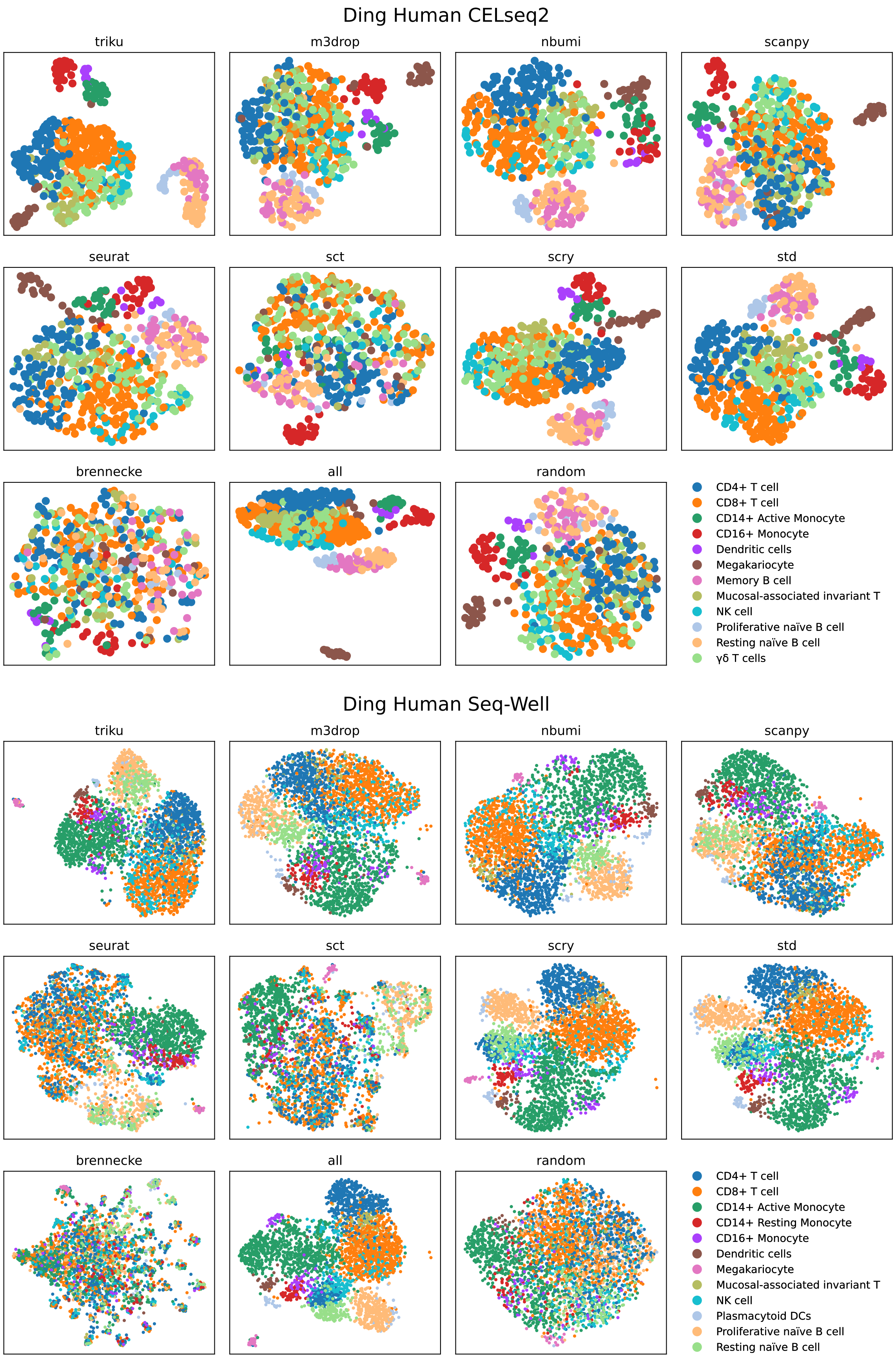

Figure 8. UMAP plots based on features selected by different methods. The UMAPs show cell subtypes from two Ding datasets in human PBMCs. For each FS method, the features were set as highly variable, and UMAP was run based on the neighborhood graph constructed from the selection of features. To assign the cell types, leiden was run to produce a high number of clusters, and a cell matching algorithm was used based on a set of markers to assign clusters to cell types. For each cell subtype, ideally, it should appear in a separate group of cells, and not be mixed with other cell subtypes.

Click here to access/download
Supplementary Material
Triku_supp_2021_09_24.pdf

Click here to access/download;Personal Cover;Comments to reviewers_2021_09_24.pdf

Reviewer #1: In the present manuscript, Ascension and colleagues introduce a new feature selection method for scRNA-seq data to increase the relevance of selected genes for downstream analysis such as clustering. I am happy to see that the tool is deposited as an open-source package that seems easy to install and plugs in seamlessly into the commonly used scanpy workflow / AnnData data structure. The documentation is sufficient to get users started. While the method has merit as a smarter approach to feature selection, the manuscript would benefit from some additional work in terms of both text and analysis.

We thank reviewer #1 for the positive and constructive comments.

Major points
1) While Triku's strategy is being introduced as superior to preexisting methods, it seems that the strong improvements (at least for the NMI summary statistic) in synthetic data turns rather incremental in the real world datasets of Mereu and Deng et al. The authors should discuss reasons for this difference. In the light of small differences and the fact that the performance is only measured in abstract summarized scores, it would be more convincing if the authors presented concrete cases where the application of Triku yields a difference in clustering or downstream analysis of biological relevance. The currently presented Gene Ontology / Geneset enrichment analysis are too diffuse and do not provide the reader with a feeling of the impact Triku could make on their analysis.

We thank the reviewer for their comment and acknowledge that some complementary clarifications and key references were missing in the original manuscript. We have added those references and modified the manuscript in order to provide the reader with a short overview of the methods that have been used in other articles to validate feature selection methods. We have included both articles introducing novel feature selection methods and articles benchmarking existing tools. We understand that these references were crucial to understand the context of our analysis.

It is very challenging to obtain evidence of superior performance of a particular feature selection method using biological datasets, for a number of reasons. On the one hand, the intrinsic read distribution of biological datasets is unknown, unlike that of artificial datasets, where it can be set by the user. On the other hand, many datasets differ in the level of granularity used in their annotation (cell types, subtypes, and states). This has an effect on accuracy: the finer the clustering the higher the chance of some clusters being misslabelled. We observe this in Ding PBMC datasets, where major cell types, such as B cells, can be subdivided into more specific cell subtypes—this point is extended later in the response, and in the revised manuscript—. Therefore, when automatic clustering finds clusters referring to minor cell types, some of these clusters will not be assigned to major cell types, and NMI values may be lower. Lastly, we are aware that Ding and Mereu datasets are heterogeneous in nature (6 to 9 different library preparation methods in 3 different tissues –PBMC, cortex and colon–, in human and mouse) and therefore, there will be unrecognizable intrinsic factors that make NMI statistics highly variable.

This is why most published Feature Selection (FS) methods up to date have either used metrics based on artificial datasets, or metrics on biological datasets while acknowledging that there is no "gold standard" to label cell populations. Here we provide a list of the references that we have added to the manuscript after revision:

- Andrews and Hemberg, 2018 [M3Drop/nbumi] used [1] data simulated from either a zero-inflated negative binomial (ZINB) to match three different Smartseq/2 datasets or data simulated from a negative binomial model with variability in library size (LS-NB), and computed AUC; [2] 5 biological datasets and computed Reproducibility (replication between HVG and genes appearing across all datasets) and Importance (Average fold-change in expression of reproducible features).
- Brennecke et al., 2013 [Brennecke] used enrichment of GO terms.
- Townes et al., 2019 [scry] [1] used calculation of ARI on Zheng 4k and 8k datasets' assigned cell types.
- Aevermann et al. 2020 used [1] visual inspection of t-SNE embeddings using the method, all and a random selection of genes.
- Su et al, 2021 used [1] ARI on 12 datasets using different number of features, comparing clustering results between SC3 defaults and SC3 using the selected features.

We believe that the results from any single analysis cannot be taken as a whole measure of truth but, rather, all the results have to be considered as a whole to provide a general view of how the different FS methods work. In that case, although individual results may be more or less conclusive, we have a strong view that *triku* works among the best for that wide range of metrics. Following the reviewer comment, we have attempted to better characterize the performance of *triku* using additional validation tests that we have included either in the revised manuscript or in the supplementary material.

Firstly, we have used ARI as a complement to NMI, which is supported in the literature [Freytag et al. 2018, Townes et al. 2019, Lall et al. 2021]. The new results are shown in the figures S2A and S3A of the revised manuscript.

Secondly, we have trained two classifiers (decision tree and kNN) using a mean 10-fold cross validation accuracy, based on [Hemphill, 2014]. The new results are shown in the figures 4C, 5C, S2C and S3C of the revised manuscript.

In order to comply with more biologically relevant tests, we have then calculated the overlap between the DEGs in different Ding Human datasets, and the selected features for different FS methods. This overlap refers to the fraction of DEGs (100 DEGs per leiden cluster) that appears also as selected by the FS method. From what we observe in the following table, for all datasets, all the DEGs

are also selected features. Therefore, we believe that using DEG overlap as a representation of goodness of the FS method is not discriminative enough.

	**10X**	**CELseq2**	**Dropseq**	**inDrop**	**Single Nuclei**	**SMARTseq2**	**Seq-Well**
**triku**	1	1	1	1	1	1	1
**m3drop**	1	1	1	1	1	1	1
**nbumi**	1	1	1	1	1	1	1
**scanpy**	1	1	1	1	1	1	1
**seurat**	1	1	1	1	1	1	1
**sct**	1	1	1	1	1	1	1
**scry**	1	1	1	1	1	1	1
**std**	1	1	1	1	1	1	1
**brennecke**	1	1	1	1	1	1	1

Thus, to gain more insight into the biological relevance of the FS methods, we compared UMAP visual appearance on different Ding Human datasets. To do that comparison we performed an in-depth cell subtype characterization, as we observed that the main cell types named in the datasets were not deep enough to appreciate details on the UMAPs. For instance, we divided B cells in 3 subtypes, added gdT and MAIT cells as subtypes of CD4/CD8/NK cells, or divided CD14+ monocytes into resting or active monocytes. All these subdivisions were based on markers shared across datasets and supported by the literature (Wilk et al. 2020, Stewart et al. 2021, Sanz et al. 2019, Shi et al. 2021). The results of this new analysis are shown in Figure 8. We observe that, in comparison with *triku*, other FS methods tend to mix cell subtypes, thus losing granularity of cell subtype characterization.

The detailed results can be observed in the 1B notebook, section “DEG and UMAP analysis” within the zenodo repository (10.5281/zenodo.4016714).

We hope the added analyses respond adequately to the request of further demonstration on Triku performance.

2) Comparison to other FS methods: Currently, the most widely used method would probably be Seurat's FindVariableFeatures. It would be good to run the presented example data also via Seurat and include it in all comparisons (eg. Fig. 3-6).

We have updated the main figures of the text to include the results from Seurat and ScTransform. From the results we conclude that the performances of Seurat and ScTransform lay within the ranges of scanpy, scry of nbumi for most of the metrics (ARI/NMI/Silhouette scores), and *triku* is still overall better than the rest of FS methods. These results are shown in the updated Figures 3, S1, 4, S2, 5 and S3, as well as in figures of secondary biological results, such as S5, 6 and S6 of the revised manuscript.

3) Precision of text: There are quite a few statements throughout the text that seem slightly inaccurate and the authors should work in their revision on precision and guiding the reader better through the background & performed work with a bit more clarity. Example: discussion of observed zeroes in UMI-data being well described by the Poisson or NB distributions was not realized by Svensson et al but rather had been described several years before. Compare Vieth et al., 2017 Bioinformatics & Chen et al., 2018 Genome Biology

We have now introduced the references stated by the reviewer, as follows: "Early studies observed that the read distribution of most of the single-cell studies could be fitted to a negative binomial (NB) [Vieth et al, 2017]. More specifically, read counts produced a zero-inflated bimodal distribution, whereas UMI counts produced a NB distribution [Chen et al, 2018]. These results were later replicated by Svensson, stating that the proportion of zeros in droplet-based scRNA-seq data, originally assumed to be dropouts, was tightly related to the mean expression of genes, following a NB curve [Svensson, 2020]."

4) One of the main assumptions of Triku is that import genes get "switched on", ie. change their state from rather not expressed to a relatively high expression level. I am wondering if the authors can comment on the performance of Triku in cases where the main difference between cells is a gradual change in already expressed genes and whether such difference might get lost/masked by the selection performed by Triku.

Thanks for this important comment. To answer the reviewer's question, we have applied triku on datasets with differentiation trajectories. The processed datasets are a Pancreas dataset, from Bastidas-Ponce et al. (2019); and a Dentate gyrus dataset, from

Hochgerner et al. (2018). Both datasets were downloaded and processed using the scvelo package. We followed the standard processing pipeline to reproduce their findings.

Additionally, we run *triku* on the processed dataset to get the features of interest, and used the scvelo.tl.rank_velocity_genes() function to obtain the relevant genes involved in the differentiation trajectory. To ensure that different kinds of genes are obtained, we ranged the min_corr parameter between 0.2 and 0.95, in 0.05 intervals.

min_corr parameter refers to the Minimum Spearman correlation coefficient between spliced and unspliced mRNAs. As explained in the RNA velocity basics page (https://scvelo.readthedocs.io/VelocityBasics/):
"The black line corresponds to the estimated ‘steady-state’ ratio, i.e. the ratio of unspliced to spliced mRNA abundance which is in a constant transcriptional state. RNA velocity for a particular gene is determined as the residual, i.e. how much an observation deviates from that steady-state line. Positive velocity indicates that a gene is up-regulated, which occurs for cells that show higher abundance of unspliced mRNA for that gene than expected in steady state. Conversely, negative velocity indicates that a gene is down-regulated."

Therefore, genes with high correlation imply that the unspliced/spliced ratio is proportional and, therefore, that the gene expression is located within the steady-state regime. Thus, that gene is not especially important in the differentiation trajectory. Genes with lower correlation values tend to show a greater unspliced/spliced ratio, and might be drivers of differentiation. The values of correlation cannot be very low (~ 0), otherwise we would be selecting random noise genes.

To compare the genes selected by triku and the genes selected by scvelo, we calculated the ratio of the set of genes detected by *triku* and scvelo, divided by all the genes selected by scvelo. This ratio, thus, explains the proportion of genes that are associated to differentiation trajectories, and which are selected by triku.

For both the pancreas and the dentate gyrus datasets, for the whole range of min_corr values, this ratio is 1, that is, all the trajectory-relevant genes are also genes detected by triku. Therefore, *triku* detects all genes associated to differentiation trajectories.

The detailed results can be observed in the 1B notebook, section “Analysis on continuous datasets” within the zenodo repository (10.5281/zenodo.4016714).

These results are expected due to the nature of triku and of the trajectory-related genes. These genes, despite being expressed in more than one cell type, are somewhat "localized" in comparison to the whole dataset, and therefore are candidates to be selected based on the rationale of triku.

Minor points
5) What is the rationale for selecting the % of zero expression as the descriptive statistics within the knn neighborhood? If a gene occurs in less cells but with higher expression, it's dispersion would be higher too. It would be needed to justify this more precisely and ideally the authors would add a version of Triku that works on dispersion (to show possible differences).

Our intention is not to use the percentage of zeros as the descriptive statistic within the kNN neighbor. We observed that excluding (or including) zeros to calculate the observed and expected count distributions for the calculation of the Wasserstein distance could have a direct effect on the distance calculation. For more information we kindly ask the reviewer to revise the “Why cells with zero counts are not considered to build the kNN count distribution” section in Supplementary Results.

Additionally, the percentage of zeros proposed in the rationale is already an accurate descriptor that acts in the same manner -if not better- than only the dispersion. For instance, in the example provided by the reviewer, a gene with high expression in fewer cells would yield a high percentage of zeros. On the other hand, a gene with low expression in few cells, in spite of having a high percentage of zeros and a low dispersion, can be a candidate for election by Triku if the expression is localized in a small subset of similar cells. In fact, this effect was described before (Wu and Zhang, 2020): "Despite having low overall expression levels within a cell, such genes [transcription factors] might be important in uncovering the fate of that cell.".

The use of the dispersion might only be inefficient for gene classification. In Supplementary Figure 8 we now show examples of genes with low, medium and high expression levels and similar dispersions, but different colocalization levels. For each level of expression, it is easy to find genes that are expressed in similar cells, and genes expressed in dissimilar cells. These later genes would not be discerned by using dispersion-based FS methods alone.

We kindly ask the reviewer to consult the 2A notebook in our zenodo repository (10.5281/zenodo.4016714) to see extended information on the rationale and initial design stages of triku.

6) Three main types of feature selection methods are introduced but not defined/explained further (p. 2)

We have included a more precise description of the methods.

7) Since Triku performs more calculations/steps than existing methods for FS, the runtime is presumably higher. The authors should compare and comment on runtime.

Thank you very much for this important remark. Following your suggestion, we have spent some more time trying to optimize the computational efficiency of our method, which has resulted in a new version (v2) of the method with improved running times. Additionally, we have added a discussion on the runtime efficiency of our method in the supplementary materials.

Computational cost optimization was done by enabling triku to work both with dense and sparse matrices, as well as by identifying and resolving bottlenecks. We also made the decision to rely the k-NN graph calculation to the original from scanpy (which also uses UMAP neighbors' function to calculate the graph). This decision was based on two factors. Firstly, oftentimes the neighbor graph is previously computed; therefore, computing it again is a waste of time that can be avoided by directly using the graph computed by scanpy. Secondly, if the user runs the neighbor graph using scanpy, and this graph is again computed using triku, the graphs might be different (due to a different default number of neighbors, or due to intrinsic differences in the heuristics of neighbor calculation of both methods). This makes the results less reproducible, since the graph computed by triku is discarded once the method is run. Therefore, for v2 we made the decision to deprecate PCA/kNN graph calculation, using the graph computed by scanpy instead. If the graph is not computed, the user has to compute it first (an error is raised).

Regarding the cost-efficiency of *triku* compared to other feature selection methods, we must consider that most feature selection methods rely on the calculation of a single univariate measurement, whose calculation is virtually immediate. However, other steps in single-cell RNAseq analysis pipelines are much more time consuming. We consider that *triku* has an acceptable running time, compared to other steps from a common single-cell RNAseq processing pipeline and that its use does not perceivably increase the overall running time of the analysis. In order to show this, we downloaded datasets with a range of cells (901 to 242106), also considering different batches, and ran a standard processing pipeline consisting of the following steps: QC and filtering, PCA, kNN, triku, UMAP/t-SNE, leiden/louvain, and PAGA. For datasets with batches, bbknn and harmony are used.

In general, we observed that *triku* shows similar runtimes to other steps in the processing pipeline, such as PCA or kNN. The results of this analysis can be found in the section "*triku* processing times" in the Supplementary Materials.

Reviewer #2: The manuscript presents a new method, Triku, for feature selection in single-cell RNA-seq data. Feature selection is performed upstream of tasks such as clustering and differential expression to reduce the effect of genes with noisy expression. Triku uses a KNN approach to identify features that are unexpected within cells that are transcriptionally close. Overall, the manuscript is well-written, presented clearly, and is a promising new method for feature selection. The figures are also very nice.

We thank reviewer #2 for the positive and constructive comments.

Major:
1. In Figure 4, it is not obvious why different methods would rank so differently between the two datasets. What methods did those papers originally use for feature selection (if available). Does that partially explain the differences?

The authors of the original papers state the following in their methods section:

- Ding et al.: "We then performed principal-component analysis using all genes, did clustering analysis (Louvain clustering of the k-nearest neighbor (k-NN) graph built from the first 50 principal components [...] followed by differential gene expression analysis with the FindAllMarkers command in Seurat to find cluster-specific (up-regulated) marker genes."
- Mereu et al.: "We selected HVGs by evaluating the relationship between gene dispersion (y.cutoff = 0.5) and the log mean expression"

Thus, Ding et al. do not use any form of feature selection (FS) method, since FindAllMarkers is a differentially expressed gene selection method; and Mereu et al. apply a method based on dispersion, such as the one based in Scanpy or Seurat. Therefore, the differences of the methods cannot be directly explained by the FS method used, since otherwise the method "all" (use all genes) would rank the highest in Ding et al., and methods such as scanpy or seurat would rank highest. However, this is not the case. We believe that the main driver in the results is the decision of cell labelling which, being semi-supervised, might not be completely accurate in the sense that some important cell subtypes might be missing (because they would be merged).

2. Figure 6, the left-most plot does not belong? It is not described in the legend.

We apologize for this omission. We have now updated the caption of the figure to explain the subplot referring to the NMI values of the datasets.

3. It would be helpful to note somewhere which category of methods the others belong to (i.e. variance based or distribution based).

We have updated the information in the Materials and Methods section of the paper. Now, in the presentation of the FS methods we include the category they belong to.

4. Some additional results and discussion on the number of genes selected. 250-500 is quite low and may explain the poor overlap between genes selected. In my experience with commonly used methods from the scran or Seurat package a more typical number of genes selected is around 2,000. What are the typical numbers used/recommended for the other methods compared to here? Does the performance difference remain when expanded to the top 2,000 genes? And is the performance better for Triku on 250 compared to 2,000?

The case of 250-500 does only apply to artificial datasets generated by scatter. Those datasets are not used for the creation of the overlap figure. Instead, for that figure, the genes selected are the ones automatically detected by *triku*. As referenced now in Supplementary Figure 1, the number of selected features for most of the datasets are in the range 1500 to 3000, which are commonly used values. Therefore, we consider that the poor overlap between features is justified based on the number of features.
As for the election of 250 to 500 features in artificial dataset, we made the choice based on the fact that the count properties (mean, distribution, noise, outliers, etc.) of artificial datasets differ from biological ones, and require a lower set of features to explain the variability of the dataset. We performed some analyses and observed that, for datasets with poor overlaps (0.0065 - 0.025) the number of automatically-selected features ranged between 400 and 700 features, which is in range with the examples of 250 and 500 features.

5. In methods, "By default, the number of features is the one automatically selected by triku." These values should be put into the supplement to get a better idea of how many genes are being selected by default.

According to the reviewer's suggestions, we have added in Supplementary Table 1 the number of features selected for each method.

Minor:
1. In Figure 3, I would label the top and bottom as A and B, I initially misread the legend as top 250 and bottom 500 genes.

For improved clarity, the suggested changes have been applied in the corrected figure.

2. What are the approximate run times a user can expect for this method?

Please see the answer to the related point 7 of reviewer #1.

Reviewer #3: This manuscript introduces a k-NN based feature selection method, Triku, as one key step to secure informative features in analyzing single cell RNA sequencing datasets. The authors argue that most of the current feature selection methods bias to the highly expressed genes instead of the actual gene markers defining the cell populations. Instead, they focus on the local signature of gene expression for each gene and compute how each of them deviates from their null distributions. The ranked gene list concerning the deviation will be derived after the median correction. The authors use Silhouette coefficient to validate their conclusion of better modularity by comparing to other methods. Additionally, the randomness and the robustness of the method are well discussed. In general, this article is well-organized and well-written. The examples of artificial and benchmark datasets showing certain aspects of improvements compared to current methods are illustrative.

Triku will be a valuable contribution to the single cell analysis field.

We thank reviewer #3 for the positive and constructive comments.

The reviewer has some minor comments to help improve the manuscript further:

1. The authors compare Triku to many other widely-used benchmark methods but excluding Seurat. Although Seurat method is adopted in Scanpy, as they claim in the "FS methods", the default flavor of Scanpy is "Seurat" instead of "Seurat_v3", the default feature selection method in the latest version of Seurat. It might be good to make it clear. Also, another alternative yet popular method, sctransform, from Seurat is not on the comparing list.

We have updated the main figures of the text to include the results from *Seurat* and *ScTransform*. From the results we conclude that *Seurat* and *ScTransform* lay within the ranges of *scanpy*, *scry* of *nbumi* for most of the metrics (ARI/NMI/Silhouette scores), and *triku* is still overall better than the rest of FS methods. These results can be observed in the updated Figures 3, S1, 4, S2, 5 and S3, as well as in figures of secondary biological results, such as S5, 6 and S6 of the revised manuscript.

2. The evidence of "we observed that in certain datasets the Wasserstein distances tend to slightly increase with the mean expression of the genes" could be shown to introduce the necessity of further correction. And the reason why the median correction outperforms other correction methods is left unexplained. For example, Seurat, which also considers binning correction method, uses mean to control the strong relationship between variability and average expression.

The choice of using median for correction instead of mean is because we could not assume that the distribution of Wasserstein distances per bin is normally distributed, hence a non-bias central descriptor is more appropriate. Nonetheless, in the version 2.1.2 of the package we have included the option for the user to choose between mean and median correction.

The comparison between mean and median for distance correction can be observed in the section “Difference between mean and median for window correction” of Supplementary Materials.

We see that for some datasets this trend is apparent and can affect gene selection, that is, genes with higher mean will be selected only because of having higher mean, which is somewhat related to the Wasserstein distance. Regarding the use of mean or median, we do not observe abrupt differences. In some cases, using mean selects highly-expressed genes. However, the overlap of selected genes (by Jaccard index) is always greater than 0.9, which implies that the most relevant genes are always selected.

3. Since the authors integrate into the pipeline the k-NN module, which is considered computationally expensive, it would be great to evaluate the time complexity/running speed compared with other methods.

Please see the answer to the related point 7 of reviewer #1.

4. Triku assumes that the local transcriptomic similarity is more likely to define cell types. Apart from clustering, which might be better-quality after Triku, it would be interesting to show any potential effects to other popular downstream analyses in the single cell field, such as trajectory inference, given that Triku is subject to locality.

Please see the answer to the related point 4 of reviewer #1.

5. Triku builds k-NN graph on UMAP all the way around. To validate the robustness of Triku, one could also discuss alternative low embedding methods like t-SNE in the section of "robustness".

In the latest version of *triku* the neighbor graph is delegated to *scanpy*, which by default uses also UMAP. The name UMAP in both the previous manner of choosing the neighbors, and the one implemented by *scanpy*, are not related to the dimensionality reduction method by itself, but rather by the fact that this neighbor graph method and the dimensionality reduction methods are located within the same package.

6. Since Triku is likely to identify locally over-expressed genes, it would be interesting to see the overlap between features selected by Triku and the differential expressed genes, if the setting is possible to arrange to make the two comparable.

Please see the answer to the related point 1 of reviewer #1.

7. In the section of previous work, some claims were made without references. For instance, "Early methods for FS in scRNA-seq data were based on the idea that genes whose expression show a greater dispersion across the dataset are the ones that best capture the biological structure of the dataset". Another example of relevant references missing is https://pubmed.ncbi.nlm.nih..

We apologize for this omission. We have now added support to that claim using the papers by Brennecke et al. (2013) and Osorio et al. (2019). "Early methods for FS in scRNA-seq data were based on the idea that genes whose expression show a greater dispersion across the dataset are the ones that best capture the biological structure of the dataset [Brennecke et al, 2013, Osorio et al, 2019]."

8. Fig. S2 does not show exact gene names. For artificial data, why those four genes are representative is left unexplained.

The exact gene names are not relevant because, for being artificial, they are represented simply as numbers. As for the choice of those genes specifically, we tested the effect on many other gene combinations and, after seeing that they had the same effect, we chose one of the combinations of 4 genes to keep the reproducibility of the plots.

9. The authors classify reference 11, the dropout-based method as "a new generation". As far as I know, the benchmark M3Drop was published in 2018.

We have modified the statement to the following: "This finding opened the path for new FS methods that would rely on genes that showed a greater than expected proportion of zeros, according to their mean expression."
