# Supplementary material for: Triku: a feature selection method based on nearest neighbors for single-cell data
Source: Gigascience. 2022 Mar 12;11:giac017. doi: 10.1093/gigascience/giac017 (PMC8917514; doi:10.1093/gigascience/giac017)
Supplement: giac017_Response_to_Reviewer_Comments_Revision_1 [file giac017_response_to_reviewer_comments_revision_1.pdf]

Author's Response To Reviewer Comments

Close

Reviewer #1: In the present manuscript, Ascension and colleagues introduce a new feature selection method for scRNA-seq data to increase the relevance of selected genes for downstream analysis such as clustering. I am happy to see that the tool is deposited as an open-source package that seems easy to install and plugs in seamlessly into the commonly used scanpy workflow / AnnData data structure. The documentation is sufficient to get users started. While the method has merit as a smarter approach to feature selection, the manuscript would benefit from some additional work in terms of both text and analysis.

We thank reviewer #1 for the positive and constructive comments.

Major points
1) While Triku's strategy is being introduced as superior to preexisting methods, it seems that the strong improvements (at least for the NMI summary statistic) in synthetic data turns rather incremental in the real world datasets of Mereu and Deng et al. The authors should discuss reasons for this difference. In the light of small differences and the fact that the performance is only measured in abstract summarized scores, it would be more convincing if the authors presented concrete cases where the application of Triku yields a difference in clustering or downstream analysis of biological relevance. The currently presented Gene Ontology / Geneset enrichment analysis are too diffuse and do not provide the reader with a feeling of the impact Triku could make on their analysis.

We thank the reviewer for their comment and acknowledge that some complementary clarifications and key references were missing in the original manuscript. We have added those references and modified the manuscript in order to provide the reader with a short overview of the methods that have been used in other articles to validate feature selection methods. We have included both articles introducing novel feature selection methods and articles benchmarking existing tools. We understand that these references were crucial to understand the context of our analysis.

It is very challenging to obtain evidence of superior performance of a particular feature selection method using biological datasets, for a number of reasons. On the one hand, the intrinsic read distribution of biological datasets is unknown, unlike that of artificial datasets, where it can be set by the user. On the other hand, many datasets differ in the level of granularity used in their annotation (cell types, subtypes, and states). This has an effect on accuracy: the finer the clustering the higher the chance of some clusters being misslabelled. We observe this in Ding PBMC datasets, where major cell types, such as B cells, can be subdivided into more specific cell subtypes—this point is extended later in the response, and in the revised manuscript—. Therefore, when automatic clustering finds clusters referring to minor cell types, some of these clusters will not be assigned to major cell types, and NMI values may be lower. Lastly, we are aware that Ding and Mereu datasets are heterogeneous in nature (6 to 9 different library preparation methods in 3 different tissues –PBMC, cortex and colon–, in human and mouse) and therefore, there will be unrecognizable intrinsic factors that make NMI statistics highly variable.

This is why most published Feature Selection (FS) methods up to date have either used metrics based on artificial datasets, or metrics on biological datasets while acknowledging that there is no "gold standard" to label cell populations. Here we provide a list of the references that we have added to the manuscript after revision:

- Andrews and Hemberg, 2018 [M3Drop/nbumi] used [1] data simulated from either a zero-inflated negative binomial (ZINB) to match three different Smartseq/2 datasets or data simulated from a negative binomial model with variability in library size (LS-NB), and computed AUC; [2] 5 biological datasets and computed Reproducibility (replication between HVG and genes appearing across all datasets) and Importance (Average fold-change in expression of reproducible features).
- Brennecke et al., 2013 [Brennecke] used enrichment of GO terms.
- Townes et al., 2019 [scry] [1] used calculation of ARI on Zheng 4k and 8k datasets' assigned cell types.

• Aevermann et al. 2020 used [1] visual inspection of t-SNE embeddings using the method, all and a random selection of genes.
• Su et al, 2021 used [1] ARI on 12 datasets using different number of features, comparing clustering results between SC3 defaults and SC3 using the selected features.

We believe that the results from any single analysis cannot be taken as a whole measure of truth but, rather, all the results have to be considered as a whole to provide a general view of how the different FS methods work. In that case, although individual results may be more or less conclusive, we have a strong view that triku works among the best for that wide range of metrics. Following the reviewer comment, we have attempted to better characterize the performance of triku using additional validation tests that we have included either in the revised manuscript or in the supplementary material.

Firstly, we have used ARI as a complement to NMI, which is supported in the literature [Freytag et al. 2018, Townes et al. 2019, Lall et al. 2021]. The new results are shown in the figures S2A and S3A of the revised manuscript.

Secondly, we have trained two classifiers (decision tree and kNN) using a mean 10-fold cross validation accuracy, based on [Hemphill, 2014]. The new results are shown in the figures 4C, 5C, S2C and S3C of the revised manuscript.

In order to comply with more biologically relevant tests, we have then calculated the overlap between the DEGs in different Ding Human datasets, and the selected features for different FS methods. This overlap refers to the fraction of DEGs (100 DEGs per leiden cluster) that appears also as selected by the FS method. From what we observe in the following table, for all datasets, all the DEGs are also selected features. Therefore, we believe that using DEG overlap as a representation of goodness of the FS method is not discriminative enough. (See table if the attached pdf of the comments to the reviewers)
Thus, to gain more insight into the biological relevance of the FS methods, we compared UMAP visual appearance on different Ding Human datasets. To do that comparison we performed an in-depth cell subtype characterization, as we observed that the main cell types named in the datasets were not deep enough to appreciate details on the UMAPs. For instance, we divided B cells in 3 subtypes, added gdT and MAIT cells as subtypes of CD4/CD8/NK cells, or divided CD14+ monocytes into resting or active monocytes. All these subdivisions were based on markers shared across datasets and supported by the literature (Wilk et al. 2020, Stewart et al. 2021, Sanz et al. 2019, Shi et al. 2021). The results of this new analysis are shown in Figure 8. We observe that, in comparison with triku, other FS methods tend to mix cell subtypes, thus losing granularity of cell subtype characterization.

The detailed results can be observed in the 1B notebook, section “DEG and UMAP analysis” within the zenodo repository (10.5281/zenodo.4016714).

We hope the added analyses respond adequately to the request of further demonstration on Triku performance.

2) Comparison to other FS methods: Currently, the most widely used method would probably be Seurat's FindVariableFeatures. It would be good to run the presented example data also via Seurat and include it in all comparisons (eg. Fig. 3-6).

We have updated the main figures of the text to include the results from Seurat and ScTransform. From the results we conclude that the performances of Seurat and ScTransform lay within the ranges of scanpy, scry of nbumi for most of the metrics (ARI/NMI/Silhouette scores), and triku is still overall better than the rest of FS methods. These results are shown in the updated Figures 3, S1, 4, S2, 5 and S3, as well as in figures of secondary biological results, such as S5, 6 and S6 of the revised manuscript.

3) Precision of text: There are quite a few statements throughout the text that seem slightly inaccurate and the authors should work in their revision on precision and guiding the reader better through the background & performed work with a bit more clarity. Example: discussion of observed zeroes in UMI-data being well described by the Poisson or NB distributions was not realized by Svensson et al but rather had been described several years before. Compare Vieth et al., 2017 Bioinformatics & Chen et al., 2018 Genome Biology

We have now introduced the references stated by the reviewer, as follows: "Early studies observed that the read distribution of most of the single-cell studies could be fitted to a negative binomial (NB) [Vieth et al, 2017]. More specifically, read counts produced a zero-inflated bimodal distribution, whereas UMI

counts produced a NB distribution [Chen et al, 2018]. These results were later replicated by Svensson, stating that the proportion of zeros in droplet-based scRNA-seq data, originally assumed to be dropouts, was tightly related to the mean expression of genes, following a NB curve [Svensson, 2020]."

4) One of the main assumptions of Triku is that import genes get "switched on", ie. change their state from rather not expressed to a relatively high expression level. I am wondering if the authors can comment on the performance of Triku in cases where the main difference between cells is a gradual change in already expressed genes and whether such difference might get lost/masked by the selection performed by Triku.

Thanks for this important comment. To answer the reviewer's question, we have applied triku on datasets with differentiation trajectories. The processed datasets are a Pancreas dataset, from Bastidas-Ponce et al. (2019); and a Dentate gyrus dataset, from Hochgerner et al. (2018). Both datasets were downloaded and processed using the scvelo package. We followed the standard processing pipeline to reproduce their findings.

Additionally, we run triku on the processed dataset to get the features of interest, and used the scvelo.tl.rank_velocity_genes() function to obtain the relevant genes involved in the differentiation trajectory. To ensure that different kinds of genes are obtained, we ranged the min_corr parameter between 0.2 and 0.95, in 0.05 intervals.

min_corr parameter refers to the Minimum Spearman correlation coefficient between spliced and unspliced mRNAs. As explained in the RNA velocity basics page (https://scvelo.readthedocs.io/VelocityBasics/):
"The black line corresponds to the estimated 'steady-state' ratio, i.e. the ratio of unspliced to spliced mRNA abundance which is in a constant transcriptional state. RNA velocity for a particular gene is determined as the residual, i.e. how much an observation deviates from that steady-state line. Positive velocity indicates that a gene is up-regulated, which occurs for cells that show higher abundance of unspliced mRNA for that gene than expected in steady state. Conversely, negative velocity indicates that a gene is down-regulated."

Therefore, genes with high correlation imply that the unspliced/spliced ratio is proportional and, therefore, that the gene expression is located within the steady-state regime. Thus, that gene is not especially important in the differentiation trajectory. Genes with lower correlation values tend to show a greater unspliced/spliced ratio, and might be drivers of differentiation. The values of correlation cannot be very low (~ 0), otherwise we would be selecting random noise genes.

To compare the genes selected by triku and the genes selected by scvelo, we calculated the ratio of the set of genes detected by triku and scvelo, divided by all the genes selected by scvelo. This ratio, thus, explains the proportion of genes that are associated to differentiation trajectories, and which are selected by triku.

For both the pancreas and the dentate gyrus datasets, for the whole range of min_corr values, this ratio is 1, that is, all the trajectory-relevant genes are also genes detected by triku. Therefore, triku detects all genes associated to differentiation trajectories.

The detailed results can be observed in the 1B notebook, section "Analysis on continuous datasets" within the zenodo repository (10.5281/zenodo.4016714).

These results are expected due to the nature of triku and of the trajectory-related genes. These genes, despite being expressed in more than one cell type, are somewhat "localized" in comparison to the whole dataset, and therefore are candidates to be selected based on the rationale of triku.

Minor points
5) What is the rationale for selecting the % of zero expression as the descriptive statistics within the knn neighborhood? If a gene occurs in less cells but with higher expression, it's dispersion would be higher too. It would be needed to justify this more precisely and ideally the authors would add a version of Triku that works on dispersion (to show possible differences).

Our intention is not to use the percentage of zeros as the descriptive statistic within the kNN neighbor. We observed that excluding (or including) zeros to calculate the observed and expected count distributions for the calculation of the Wasserstein distance could have a direct effect on the distance

calculation. For more information we kindly ask the reviewer to revise the “Why cells with zero counts are not considered to build the kNN count distribution” section in Supplementary Results.

Additionally, the percentage of zeros proposed in the rationale is already an accurate descriptor that acts in the same manner -if not better- than only the dispersion. For instance, in the example provided by the reviewer, a gene with high expression in fewer cells would yield a high percentage of zeros. On the other hand, a gene with low expression in few cells, in spite of having a high percentage of zeros and a low dispersion, can be a candidate for election by Triku if the expression is localized in a small subset of similar cells. In fact, this effect was described before (Wu and Zhang, 2020): "Despite having low overall expression levels within a cell, such genes [transcription factors] might be important in uncovering the fate of that cell.".

The use of the dispersion might only be inefficient for gene classification. In Supplementary Figure 8 we now show examples of genes with low, medium and high expression levels and similar dispersions, but different colocalization levels. For each level of expression, it is easy to find genes that are expressed in similar cells, and genes expressed in dissimilar cells. These later genes would not be discerned by using dispersion-based FS methods alone.

We kindly ask the reviewer to consult the 2A notebook in our zenodo repository (10.5281/zenodo.4016714) to see extended information on the rationale and initial design stages of triku.

6) Three main types of feature selection methods are introduced but not defined/explained further (p. 2)

We have included a more precise description of the methods.

7) Since Triku performs more calculations/steps than existing methods for FS, the runtime is presumably higher. The authors should compare and comment on runtime.

Thank you very much for this important remark. Following your suggestion, we have spent some more time trying to optimize the computational efficiency of our method, which has resulted in a new version (v2) of the method with improved running times. Additionally, we have added a discussion on the runtime efficiency of our method in the supplementary materials.

Computational cost optimization was done by enabling triku to work both with dense and sparse matrices, as well as by identifying and resolving bottlenecks. We also made the decision to rely the k-NN graph calculation to the original from scanpy (which also uses UMAP neighbors’ function to calculate the graph). This decision was based on two factors. Firstly, oftentimes the neighbor graph is previously computed; therefore, computing it again is a waste of time that can be avoided by directly using the graph computed by scanpy. Secondly, if the user runs the neighbor graph using scanpy, and this graph is again computed using triku, the graphs might be different (due to a different default number of neighbors, or due to intrinsic differences in the heuristics of neighbor calculation of both methods). This makes the results less reproducible, since the graph computed by triku is discarded once the method is run. Therefore, for v2 we made the decision to deprecate PCA/kNN graph calculation, using the graph computed by scanpy instead. If the graph is not computed, the user has to compute it first (an error is raised).

Regarding the cost-efficiency of triku compared to other feature selection methods, we must consider that most feature selection methods rely on the calculation of a single univariate measurement, whose calculation is virtually immediate. However, other steps in single-cell RNAseq analysis pipelines are much more time consuming. We consider that triku has an acceptable running time, compared to other steps from a common single-cell RNAseq processing pipeline and that its use does not perceivably increase the overall running time of the analysis. In order to show this, we downloaded datasets with a range of cells (901 to 242106), also considering different batches, and ran a standard processing pipeline consisting of the following steps: QC and filtering, PCA, kNN, triku, UMAP/t-SNE, leiden/louvain, and PAGA. For datasets with batches, bbknn and harmony are used.

In general, we observed that triku shows similar runtimes to other steps in the processing pipeline, such as PCA or kNN. The results of this analysis can be found in the section “triku processing times” in the Supplementary Materials.

Reviewer #2: The manuscript presents a new method, Triku, for feature selection in single-cell RNA-seq data. Feature selection is performed upstream of tasks such as clustering and differential expression to reduce the effect of genes with noisy expression. Triku uses a KNN approach to identify features that are unexpected within cells that are transcriptionally close. Overall, the manuscript is well-written, presented clearly, and is a promising new method for feature selection. The figures are also very nice.

We thank reviewer #2 for the positive and constructive comments.

Major:

1. In Figure 4, it is not obvious why different methods would rank so differently between the two datasets. What methods did those papers originally use for feature selection (if available). Does that partially explain the differences?

The authors of the original papers state the following in their methods section:

- Ding et al.: "We then performed principal-component analysis using all genes, did clustering analysis (Louvain clustering of the k-nearest neighbor (k-NN) graph built from the first 50 principal components [...] followed by differential gene expression analysis with the FindAllMarkers command in Seurat to find cluster-specific (up-regulated) marker genes."
- Mereu et al.: "We selected HVGs by evaluating the relationship between gene dispersion (y.cutoff = 0.5) and the log mean expression"

Thus, Ding et al. do not use any form of feature selection (FS) method, since FindAllMarkers is a differentially expressed gene selection method; and Mereu et al. apply a method based on dispersion, such as the one based in Scanpy or Seurat. Therefore, the differences of the methods cannot be directly explained by the FS method used, since otherwise the method "all" (use all genes) would rank the highest in Ding et al., and methods such as scanpy or seurat would rank highest. However, this is not the case. We believe that the main driver in the results is the decision of cell labelling which, being semi-supervised, might not be completely accurate in the sense that some important cell subtypes might be missing (because they would be merged).

2. Figure 6, the left-most plot does not belong? It is not described in the legend.

We apologize for this omission. We have now updated the caption of the figure to explain the subplot referring to the NMI values of the datasets.

3. It would be helpful to note somewhere which category of methods the others belong to (i.e. variance based or distribution based).

We have updated the information in the Materials and Methods section of the paper. Now, in the presentation of the FS methods we include the category they belong to.

4. Some additional results and discussion on the number of genes selected. 250-500 is quite low and may explain the poor overlap between genes selected. In my experience with commonly used methods from the scran or Seurat package a more typical number of genes selected is around 2,000. What are the typical numbers used/recommended for the other methods compared to here? Does the performance difference remain when expanded to the top 2,000 genes? And is the performance better for Triku on 250 compared to 2,000?

The case of 250-500 does only apply to artificial datasets generated by scatter. Those datasets are not used for the creation of the overlap figure. Instead, for that figure, the genes selected are the ones automatically detected by triku. As referenced now in Supplementary Figure 1, the number of selected features for most of the datasets are in the range 1500 to 3000, which are commonly used values. Therefore, we consider that the poor overlap between features is justified based on the number of features.
As for the election of 250 to 500 features in artificial dataset, we made the choice based on the fact that the count properties (mean, distribution, noise, outliers, etc.) of artificial datasets differ from biological ones, and require a lower set of features to explain the variability of the dataset. We performed some analyses and observed that, for datasets with poor overlaps (0.0065 - 0.025) the number of automatically-selected features ranged between 400 and 700 features, which is in range with the examples of 250 and 500 features.

5. In methods, "By default, the number of features is the one automatically selected by triku." These

values should be put into the supplement to get a better idea of how many genes are being selected by default.

According to the reviewer's suggestions, we have added in Supplementary Table 1 the number of features selected for each method.

Minor:
1. In Figure 3, I would label the top and bottom as A and B, I initially misread the legend as top 250 and bottom 500 genes.

For improved clarity, the suggested changes have been applied in the corrected figure.

2. What are the approximate run times a user can expect for this method?

Please see the answer to the related point 7 of reviewer #1.

Reviewer #3: This manuscript introduces a k-NN based feature selection method, Triku, as one key step to secure informative features in analyzing single cell RNA sequencing datasets. The authors argue that most of the current feature selection methods bias to the highly expressed genes instead of the actual gene markers defining the cell populations. Instead, they focus on the local signature of gene expression for each gene and compute how each of them deviates from their null distributions. The ranked gene list concerning the deviation will be derived after the median correction. The authors use Silhouette coefficient to validate their conclusion of better modularity by comparing to other methods. Additionally, the randomness and the robustness of the method are well discussed. In general, this article is well-organized and well-written. The examples of artificial and benchmark datasets showing certain aspects of improvements compared to current methods are illustrative.

Triku will be a valuable contribution to the single cell analysis field.

We thank reviewer #3 for the positive and constructive comments.

The reviewer has some minor comments to help improve the manuscript further:
1. The authors compare Triku to many other widely-used benchmark methods but excluding Seurat. Although Seurat method is adopted in Scanpy, as they claim in the "FS methods", the default flavor of Scanpy is "Seurat" instead of "Seurat_v3", the default feature selection method in the latest version of Seurat. It might be good to make it clear. Also, another alternative yet popular method, sctransform, from Seurat is not on the comparing list.

We have updated the main figures of the text to include the results from Seurat and ScTransform. From the results we conclude that Seurat and ScTransform lay within the ranges of scanpy, scry of nbumi for most of the metrics (ARI/NMI/Silhouette scores), and triku is still overall better than the rest of FS methods. These results can be observed in the updated Figures 3, S1, 4, S2, 5 and S3, as well as in figures of secondary biological results, such as S5, 6 and S6 of the revised manuscript.

2. The evidence of "we observed that in certain datasets the Wasserstein distances tend to slightly increase with the mean expression of the genes" could be shown to introduce the necessity of further correction. And the reason why the median correction outperforms other correction methods is left unexplained. For example, Seurat, which also considers binning correction method, uses mean to control the strong relationship between variability and average expression.

The choice of using median for correction instead of mean is because we could not assume that the distribution of Wasserstein distances per bin is normally distributed, hence a non-bias central descriptor is more appropriate. Nonetheless, in the version 2.1.2 of the package we have included the option for the user to choose between mean and median correction.

The comparison between mean and median for distance correction can be observed in the section “Difference between mean and median for window correction” of Supplementary Materials.

We see that for some datasets this trend is apparent and can affect gene selection, that is, genes with higher mean will be selected only because of having higher mean, which is somewhat related to the Wasserstein distance. Regarding the use of mean or median, we do not observe abrupt differences. In

some cases, using mean selects highly-expressed genes. However, the overlap of selected genes (by Jaccard index) is always greater than 0.9, which implies that the most relevant genes are always selected.

3. Since the authors integrate into the pipeline the k-NN module, which is considered computationally expensive, it would be great to evaluate the time complexity/running speed compared with other methods.

Please see the answer to the related point 7 of reviewer #1.

4. Triku assumes that the local transcriptomic similarity is more likely to define cell types. Apart from clustering, which might be better-quality after Triku, it would be interesting to show any potential effects to other popular downstream analyses in the single cell field, such as trajectory inference, given that Triku is subject to locality.

Please see the answer to the related point 4 of reviewer #1.

5. Triku builds k-NN graph on UMAP all the way around. To validate the robustness of Triku, one could also discuss alternative low embedding methods like t-SNE in the section of "robustness".

In the latest version of triku the neighbor graph is delegated to scanpy, which by default uses also UMAP. The name UMAP in both the previous manner of choosing the neighbors, and the one implemented by scanpy, are not related to the dimensionality reduction method by itself, but rather by the fact that this neighbor graph method and the dimensionality reduction methods are located within the same package.

6. Since Triku is likely to identify locally over-expressed genes, it would be interesting to see the overlap between features selected by Triku and the differential expressed genes, if the setting is possible to arrange to make the two comparable.

Please see the answer to the related point 1 of reviewer #1.

7. In the section of previous work, some claims were made without references. For instance, "Early methods for FS in scRNA-seq data were based on the idea that genes whose expression show a greater dispersion across the dataset are the ones that best capture the biological structure of the dataset". Another example of relevant references missing is https://pubmed.ncbi.nlm.nih..

We apologize for this omission. We have now added support to that claim using the papers by Brennecke et al. (2013) and Osorio et al. (2019). "Early methods for FS in scRNA-seq data were based on the idea that genes whose expression show a greater dispersion across the dataset are the ones that best capture the biological structure of the dataset [Brennecke et al, 2013, Osorio et al, 2019]."

8. Fig. S2 does not show exact gene names. For artificial data, why those four genes are representative is left unexplained.

The exact gene names are not relevant because, for being artificial, they are represented simply as numbers. As for the choice of those genes specifically, we tested the effect on many other gene combinations and, after seeing that they had the same effect, we chose one of the combinations of 4 genes to keep the reproducibility of the plots.

9. The authors classify reference 11, the dropout-based method as "a new generation". As far as I know, the benchmark M3Drop was published in 2018.

We have modified the statement to the following: "This finding opened the path for new FS methods that would rely on genes that showed a greater than expected proportion of zeros, according to their mean expression."

Close
