# Supplementary material for: Triku: a feature selection method based on nearest neighbors for single-cell data
Source: Gigascience. 2022 Mar 12;11:giac017. doi: 10.1093/gigascience/giac017 (PMC8917514; doi:10.1093/gigascience/giac017)
Supplement: giac017_Response_to_Reviewer_Comments_Revision_2 [file giac017_response_to_reviewer_comments_revision_2.pdf]

Author's Response To Reviewer Comments

Close

Reviewer reports:
Reviewer #1: In this revision, Ascension and colleagues have clearly worked on improving the manuscript.
As with the initial submission, I think there is value in Triku as a new alternative in the large toolbox of available single-cell RNA-seq analysis steps. While the authors have made some steps forward in adding substance to the evaluation of Triku, some further work should be done as outlined below.

As in my initial review, I still think the text would benefit from more work in terms of precision of statements and guiding the reader through the work. I appreciate that the authors have expanded the discussion regarding proportion of zeroes / NB distribution of scRNA-seq data mentioned in the first review, but the comment was meant to be more general throughout the text.

Answer
We agree with the reviewer in that some sentences in the introduction lacked precision, and we have reworked some parts of the manuscript in order to improve its clarity. We have made an effort to provide a more sensible ground and to make the reasoning behind Triku more explicit.

Examples:
"...their sparsity, which are worsened in single-cell datasets due the high proportion of zeros from low signal recovery" - There are huge differences between methods in scRNA-seq with some recovering in the order of ~1000s of RNA molecules to ~100,000 RNA molecules

We have added a more precise explanation of the sparsity in single-cell RNA-seq and its possible sources. Also, we acknowledge the fact that there is great variability between methods regarding their RNA recovery efficiency.

Before: scRNA-seq datasets are multidimensional, i.e. the expression profile per cell consists of multiple genes. Two common characteristics of multidimensional datasets are their high dimensionality and their sparsity, which are worsened in single-cell datasets due the high proportion of zeros from low signal recovery \cite{bzdok_altman_krzywinski_2018}. This sparsity affects downstream methods such as cell type detection or differential gene expression

After: Gene expression datasets are highly dimensional, as the expression of tens of thousands of genes is measured in any given experiment. A direct consequence of this is the curse of dimensionality, where the amount of data needed to fill the sampling space increases exponentially with the dimensions, resulting in a sparsity of the data \cite{bellman_2015}. Additionally, this sparsity is exacerbated by the low capture efficiency of mRNA in single-cell experiments, due to the tiny amounts genetic material to be amplified, even though there are considerable recovery differences across methods \cite{bzdok_altman_krzywinski_2018}. This sparsity affects downstream methods such as cell type detection or differential gene expression \cite{heimberg_bhatnagar_el_samad_thomson_2016}.

"However, we observe that there are three main patterns of expression according to the distribution of zeros of a particular gene and overall transcriptional similarity (expression of all genes)" - This should be more along the lines of "We consider three main cases of expression patterns" since these are just hypothetical/assumption & and not derived from an analysis presented here.

We have rephrased the text as the reviewer suggestion, which seems more appropriate.

Before: However, we observe that there are three main patterns of expression according to the distribution of zeros of a particular gene and overall transcriptional similarity (expression of all genes)
[...]

After: However, we consider that there are three main patterns of expression according to the distribution of zeros of a particular gene and overall transcriptional similarity (expression of all genes) [...]

Nonetheless, those observations are not hypothetical and can be observed in Supp. Fig. 8, where Lyz2 /Vtn and Cog3 are examples of genes expressed in a few cells, but Cog3 not as focused to a subset of cells as Lyz2 or Vtn (Cog3 would be b1 in Figure 1, and Lyz2/Vtn would be b2); and Dhx30 is a clear example of case a from Figure 1.

We have included a similar statement following to the previous one: “These patterns can be seen, for instance, in genes Dhx30, Cog3 and Lyz2 in Figure S8.”

Run time of Triku: The comment in my initial review was not meant as a criticism. Even though a supplementary note was added, it would be much more transparent for users to discuss this in the main text.

We thank the reviewer for the suggestion. We do agree that the information regarding the run-time efficiency analysis was missing in the manuscript. Therefore, we have mention the run-time analysis at the start of the methods so that readers can easily localize the full analysis in the Supplementary Material. In general, we have tried to focus on the outputs and evaluation of Triku rather than the technical aspects, in order to make the article more readable to the putative users of our software. However, we do agree that the methodological aspects of Triku are of interest to researchers and developers of new methods and need to be available.

While the response to reviewers file contained a lot of text from the authors regarding the disparity between metrics, it would be much better to add some of this discussion to the manuscript for readers to get a better feeling.

We agree with the reviewer in that a general explanation of the range of metrics was missing. We have, thus, added the following paragraph in the discussion:

“In general, a single metric is not sufficient to properly evaluate a novel computational method but rather, all the results have to be considered as a whole to provide a general view of how the different FS methods work. In the present work, we have used a number of metrics (NMI, ARI, Silhouette, cross-validation accuracy of two supervised classifiers—decision tree and kNN--, and degree of separation of distinct cell populations on UMAP plots). Although individual results may be more or less conclusive, we have a strong view that Triku works among the best for that wide range of metrics.”

FS method gene set overlap: The comment on the lack of correlation between clustering solution and gene set overlap seems a bit out of place and anecdotal.

Although contradictory, we believe these results are very important to provide context to the reader about the feature redundancy of biological datasets, extended in discussion. We think that some researchers that are new to the field might not be familiar with the fact that it is possible to distinguish populations in a scRNA-seq dataset using two completely different sets of genes, as a cause of this redundancy. Additionally, none of the already published papers on FS methods address this issue, which we believe is important.
Reviewer #2: All major comments are satisfied by the author's response and updated manuscript. Two minor comments:

1. For Figure 3, the caption should be changed from "top 250 (top) and 500 (bottom) features for each FS methods" to "top 250 (A) and 500 (B) features of each FS method."

We thank the reviewer for the appreciation. We have changed the labels on the figure.

2. Were the same parameters used to generate UMAP dimensions across all methods in Figure 8? This information should be added to the methods section either way.

Yes, all UMAPs from Figure 8 we created on the same parameters. All of the parameters can be found on the corresponding notebooks for replication purposes. Additionally, this information has been added to

the methods and into the caption of Figure 8.

Close
