# Supplementary material for: Triku: a feature selection method based on nearest neighbors for single-cell data
Source: Gigascience. 2022 Mar 12;11:giac017. doi: 10.1093/gigascience/giac017 (PMC8917514; doi:10.1093/gigascience/giac017)
Supplement: giac017_Response_to_Reviewer_Comments_Revision_3 [file giac017_response_to_reviewer_comments_revision_3.pdf]

Author's Response To Reviewer Comments

Close

Dear Hans,

We have add our new GigaDB repository to the bibliography, including the doi link.
And included Github and Zenodo repositories in the bibliography and citing them by number from the main text.

All the best,

Marcos

Close
