# Supplementary material for: Triku: a feature selection method based on nearest neighbors for single-cell data
Source: Gigascience. 2022 Mar 12;11:giac017. doi: 10.1093/gigascience/giac017 (PMC8917514; doi:10.1093/gigascience/giac017)
Supplement: giac017_Reviewer_1_Report_Original_Submission — Christoph Ziegenhain -- 6/9/2021 Reviewed [file giac017_reviewer_1_report_original_submission.pdf]

Reviewer Report

Title: Triku: a feature selection method based on nearest neighbors for single-cell data

Version: Original Submission **Date:** 6/9/2021

Reviewer name: Christoph Ziegenhain

Reviewer Comments to Author:

In the present manuscript, Ascension and colleagues introduce a new feature selection method for scRNA-seq data to increase the relevance of selected genes for downstream analysis such as clustering. I am happy to see that the tool is deposited as an open-source package that seems easy to install and plugs in seamlessly into the commonly used scanpy workflow / AnnData data structure. The documentation is sufficient to get users started. While the method has merit as a smarter approach to feature selection, the manuscript would benefit from some additional work in terms of both text and analysis.

Major points

1) While Triku's strategy is being introduced as superior to preexisting methods, it seems that the strong improvements (at least for the NMI summary statistic) in synthetic data turns rather incremental in the real world datasets of Mereu and Deng et al. The authors should discuss reasons for this difference. In the light of small differences and the fact that the performance is only measured in abstract summarized scores, it would be more convincing if the authors presented concrete cases where the application of Triku yields a difference in clustering or downstream analysis of biological relevance. The currently presented Gene Ontology / Geneset enrichment analysis are too diffuse and do not provide the reader with a feeling of the impact Triku could make on their analysis.

2) Comparison to other FS methods: Currently, the most widely used method would probably be Seurat's FindVariableFeatures. It would be good to run the presented example data also via Seurat and include it in all comparisons (eg. Fig. 3-6).

3) Precision of text: There are quite a few statements throughout the text that seem slightly inaccurate and the authors should work in their revision on precision and guiding the reader better through the background & performed work with a bit more clarity. Example: discussion of observed zeroes in UMI-data being well described by the Poisson or NB distributions was not realized by Svensson et al but rather had been described several years before. Compare Vieth et al., 2017 Bioinformatics & Chen et al., 2018 Genome Biology

4) One of the main assumptions of Triku is that import genes get "switched on", ie. change their state from rather not expressed to a relatively high expression level. I am wondering if the authors can comment on the performance of Triku in cases where the main difference between cells is a gradual change in already expressed genes and whether such difference might get lost/masked by the selection performed by Triku.

Minor points

5) What is the rationale for selecting the % of zero expression as the descriptive statistics within the knn neighborhood? If a gene occurs in less cells but with higher expression, it's dispersion would be higher

too. It would be needed to justify this more precisely and ideally the authors would add a version of Triku that works on dispersion (to show possible differences).
6) Three main types of feature selection methods are introduced but not defined/explained further (p. 2)
7) Since Triku performs more calculations/steps than existing methods for FS, the runtime is presumably higher. The authors should compare and comment on runtime.

Level of Interest

Please indicate how interesting you found the manuscript: Choose an item.

Quality of Written English

Please indicate the quality of language in the manuscript: Choose an item.

Declaration of Competing Interests

Please complete a declaration of competing interests, considering the following questions:

- Have you in the past five years received reimbursements, fees, funding, or salary from an organisation that may in any way gain or lose financially from the publication of this manuscript, either now or in the future?
- Do you hold any stocks or shares in an organisation that may in any way gain or lose financially from the publication of this manuscript, either now or in the future?
- Do you hold or are you currently applying for any patents relating to the content of the manuscript?
- Have you received reimbursements, fees, funding, or salary from an organization that holds or has applied for patents relating to the content of the manuscript?
- Do you have any other financial competing interests?
- Do you have any non-financial competing interests in relation to this paper?

If you can answer no to all of the above, write 'I declare that I have no competing interests' below. If your reply is yes to any, please give details below.

I declare that I have no competing interests

I agree to the open peer review policy of the journal. I understand that my name will be included on my report to the authors and, if the manuscript is accepted for publication, my named report including any attachments I upload will be posted on the website along with the authors' responses. I agree for my report to be made available under an Open Access Creative Commons CC-BY license (http://creativecommons.org/licenses/by/4.0/). I understand that any comments which I do not wish to

be included in my named report can be included as confidential comments to the editors, which will not be published.

Choose an item.

To further support our reviewers, we have joined with Publons, where you can gain additional credit to further highlight your hard work (see: https://publons.com/journal/530/gigascience). On publication of this paper, your review will be automatically added to Publons, you can then choose whether or not to claim your Publons credit. I understand this statement.

Yes Choose an item.
