# Supplementary material for: Triku: a feature selection method based on nearest neighbors for single-cell data
Source: Gigascience. 2022 Mar 12;11:giac017. doi: 10.1093/gigascience/giac017 (PMC8917514; doi:10.1093/gigascience/giac017)
Supplement: giac017_Reviewer_1_Report_Revision_1 — Christoph Ziegenhain -- 10/8/2021 Reviewed [file giac017_reviewer_1_report_revision_1.pdf]

Reviewer Report

Title: Triku: a feature selection method based on nearest neighbors for single-cell data

Version: Revision 1 **Date:** 10/8/2021

Reviewer name: Christoph Ziegenhain

Reviewer Comments to Author:

In this revision, Ascension and colleagues have clearly worked on improving the manuscript.
As with the initial submission, I think there is value in Triku as a new alternative in the large toolbox of available single-cell RNA-seq analysis steps. While the authors have made some steps forward in adding substance to the evaluation of Triku, some further work should be done as outlined below.
As in my initial review, I still think the text would benefit from more work in terms of precision of statements and guiding the reader through the work. I appreciate that the authors have expanded the discussion regarding proportion of zeroes / NB distribution of scRNA-seq data mentioned in the first review, but the comment was meant to be more general throughout the text.
Examples:
"...their sparsity, which are worsened in single-cell datasets due the high proportion of zeros from low signal recovery" - There are huge differences between methods in scRNA-seq with some recovering in the order of ~1000s of RNA molecules to ~100,000 RNA molecules
"However, we observe that there are three main patterns of expression according to the distribution of zeros of a particular gene and overall transcriptional similarity (expression of all genes)" - This should be more along the lines of "We consider three main cases of expression patterns" since these are just hypothetical/assumption & and not derived from an analysis presented here.
Run time of Triku: The comment in my initial review was not meant as a criticism. Even though a supplementary note was added, it would be much more transparent for users to discuss this in the main text.
While the response to reviewers file contained a lot of text from the authors regarding the disparity between metrics, it would be much better to add some of this discussion to the manuscript for readers to get a better feeling.
FS method gene set overlap: The comment on the lack of correlation between clustering solution and gene set overlap seems a bit out of place and anecdotal.

Level of Interest

Please indicate how interesting you found the manuscript: Choose an item.

Quality of Written English

Please indicate the quality of language in the manuscript: Choose an item.

Declaration of Competing Interests

Please complete a declaration of competing interests, considering the following questions:

- Have you in the past five years received reimbursements, fees, funding, or salary from an organisation that may in any way gain or lose financially from the publication of this manuscript, either now or in the future?
- Do you hold any stocks or shares in an organisation that may in any way gain or lose financially from the publication of this manuscript, either now or in the future?
- Do you hold or are you currently applying for any patents relating to the content of the manuscript?
- Have you received reimbursements, fees, funding, or salary from an organization that holds or has applied for patents relating to the content of the manuscript?
- Do you have any other financial competing interests?
- Do you have any non-financial competing interests in relation to this paper?

If you can answer no to all of the above, write 'I declare that I have no competing interests' below. If your reply is yes to any, please give details below.

I declare that I have no competing interests

I agree to the open peer review policy of the journal. I understand that my name will be included on my report to the authors and, if the manuscript is accepted for publication, my named report including any attachments I upload will be posted on the website along with the authors' responses. I agree for my report to be made available under an Open Access Creative Commons CC-BY license (http://creativecommons.org/licenses/by/4.0/). I understand that any comments which I do not wish to be included in my named report can be included as confidential comments to the editors, which will not be published.

Choose an item.

To further support our reviewers, we have joined with Publons, where you can gain additional credit to further highlight your hard work (see: https://publons.com/journal/530/gigascience). On publication of this paper, your review will be automatically added to Publons, you can then choose whether or not to claim your Publons credit. I understand this statement.

Yes Choose an item.
