# Supplementary material for: Triku: a feature selection method based on nearest neighbors for single-cell data
Source: Gigascience. 2022 Mar 12;11:giac017. doi: 10.1093/gigascience/giac017 (PMC8917514; doi:10.1093/gigascience/giac017)
Supplement: giac017_Reviewer_2_Report_Original_Submission — Rhonda Bacher -- 6/21/2021 Reviewed [file giac017_reviewer_2_report_original_submission.pdf]

Reviewer Report

Title: Triku: a feature selection method based on nearest neighbors for single-cell data

Version: Original Submission **Date:** 6/21/2021

Reviewer name: Rhonda Bacher

Reviewer Comments to Author:

The manuscript presents a new method, Triku, for feature selection in single-cell RNA-seq data. Feature selection is performed upstream of tasks such as clustering and differential expression to reduce the effect of genes with noisy expression. Triku uses a KNN approach to identify features that are unexpected within cells that are transcriptionally close. Overall, the manuscript is well-written, presented clearly, and is a promising new method for feature selection. The figures are also very nice.

Major:

1. In Figure 4, it is not obvious why different methods would rank so differently between the two datasets. What methods did those papers originally use for feature selection (if available). Does that partially explain the differences?
2. Figure 6, the left-most plot does not belong? It is not described in the legend.
3. It would be helpful to note somewhere which category of methods the others belong to (i.e. variance based or distribution based).
4. Some additional results and discussion on the number of genes selected. 250-500 is quite low and may explain the poor overlap between genes selected. In my experience with commonly used methods from the scran or Seurat package a more typical number of genes selected is around 2,000. What are the typical numbers used/recommended for the other methods compared to here? Does the performance difference remain when expanded to the top 2,000 genes? And is the performance better for Triku on 250 compared to 2,000?
5. In methods, "By default, the number of features is the one automatically selected by triku." These values should be put into the supplement to get a better idea of how many genes are being selected by default.

Minor:

1. In Figure 3, I would label the top and bottom as A and B, I initially misread the legend as top 250 and bottom 500 genes.
2. What are the approximate run times a user can expect for this method?

Level of Interest

Please indicate how interesting you found the manuscript: Choose an item.

Quality of Written English

Please indicate the quality of language in the manuscript: Choose an item.

Declaration of Competing Interests

Please complete a declaration of competing interests, considering the following questions:

- Have you in the past five years received reimbursements, fees, funding, or salary from an organisation that may in any way gain or lose financially from the publication of this manuscript, either now or in the future?
- Do you hold any stocks or shares in an organisation that may in any way gain or lose financially from the publication of this manuscript, either now or in the future?
- Do you hold or are you currently applying for any patents relating to the content of the manuscript?
- Have you received reimbursements, fees, funding, or salary from an organization that holds or has applied for patents relating to the content of the manuscript?
- Do you have any other financial competing interests?
- Do you have any non-financial competing interests in relation to this paper?

If you can answer no to all of the above, write 'I declare that I have no competing interests' below. If your reply is yes to any, please give details below.

I declare that I have no competing interests.

I agree to the open peer review policy of the journal. I understand that my name will be included on my report to the authors and, if the manuscript is accepted for publication, my named report including any attachments I upload will be posted on the website along with the authors' responses. I agree for my report to be made available under an Open Access Creative Commons CC-BY license (http://creativecommons.org/licenses/by/4.0/). I understand that any comments which I do not wish to be included in my named report can be included as confidential comments to the editors, which will not be published.

Choose an item.

To further support our reviewers, we have joined with Publons, where you can gain additional credit to further highlight your hard work (see: https://publons.com/journal/530/gigascience). On publication of this paper, your review will be automatically added to Publons, you can then choose whether or not to claim your Publons credit. I understand this statement.

Yes Choose an item.
