# Supplementary material for: Triku: a feature selection method based on nearest neighbors for single-cell data
Source: Gigascience. 2022 Mar 12;11:giac017. doi: 10.1093/gigascience/giac017 (PMC8917514; doi:10.1093/gigascience/giac017)
Supplement: giac017_Reviewer_3_Report_Original_Submission — James J Cai -- 6/26/2021 Reviewed [file giac017_reviewer_3_report_original_submission.pdf]

Reviewer Report

Title: Triku: a feature selection method based on nearest neighbors for single-cell data

Version: Original Submission **Date:** 6/26/2021

Reviewer name: James J Cai

Reviewer Comments to Author:

This manuscript introduces a k-NN based feature selection method, Triku, as one key step to secure informative features in analyzing single cell RNA sequencing datasets. The authors argue that most of the current feature selection methods bias to the highly expressed genes instead of the actual gene markers defining the cell populations. Instead, they focus on the local signature of gene expression for each gene and compute how each of them deviates from their null distributions. The ranked gene list concerning the deviation will be derived after the median correction. The authors use Silhouette coefficient to validate their conclusion of better modularity by comparing to other methods. Additionally, the randomness and the robustness of the method are well discussed. In general, this article is well-organized and well-written. The examples of artificial and benchmark datasets showing certain aspects of improvements compared to current methods are illustrative. Triku will be a valuable contribution to the single cell analysis field. The reviewer has some minor comments to help improve the manuscript further:

1. The authors compare Triku to many other widely-used benchmark methods but excluding Seurat. Although Seurat method is adopted in Scanpy, as they claim in the "FS methods", the default flavor of Scanpy is "Seurat" instead of "Seurat_v3", the default feature selection method in the latest version of Seurat. It might be good to make it clear. Also, another alternative yet popular method, sctransform, from Seurat is not on the comparing list.
2. The evidence of "we observed that in certain datasets the Wasserstein distances tend to slightly increase with the mean expression of the genes" could be shown to introduce the necessity of further correction. And the reason why the median correction outperforms other correction methods is left unexplained. For example, Seurat, which also considers binning correction method, uses mean to control the strong relationship between variability and average expression.
3. Since the authors integrate into the pipeline the k-NN module, which is considered computationally expensive, it would be great to evaluate the time complexity/running speed compared with other methods.
4. Triku assumes that the local transcriptomic similarity is more likely to define cell types. Apart from clustering, which might be better-quality after Triku, it would be interesting to show any potential effects to other popular downstream analyses in the single cell field, such as trajectory inference, given that Triku is subject to locality.
5. Triku builds k-NN graph on UMAP all the way around. To validate the robustness of Triku, one could also discuss alternative low embedding methods like t-SNE in the section of "robustness".
6. Since Triku is likely to identify locally over-expressed genes, it would be interesting to see the overlap between features selected by Triku and the differential expressed genes, if the setting is possible to

arrange to make the two comparable.
7. In the section of previous work, some claims were made without references. For instance, "Early methods for FS in scRNA-seq data were based on the idea that genes whose expression show a greater dispersion across the dataset are the ones that best capture the biological structure of the dataset". Another example of relevant references missing is https://pubmed.ncbi.nlm.nih.gov/31861624/.
8. Fig. S2 does not show exact gene names. For artificial data, why those four genes are representative is left unexplained.
9. The authors classify reference 11, the dropout-based method as "a new generation". As far as I know, the benchmark M3Drop was published in 2018.
This Reviewer's comments were prepared with assistance from my graduate student Yongjian Yang.

Level of Interest

Please indicate how interesting you found the manuscript: Choose an item.

Quality of Written English

Please indicate the quality of language in the manuscript: Choose an item.

Declaration of Competing Interests

Please complete a declaration of competing interests, considering the following questions:

- Have you in the past five years received reimbursements, fees, funding, or salary from an organisation that may in any way gain or lose financially from the publication of this manuscript, either now or in the future?
- Do you hold any stocks or shares in an organisation that may in any way gain or lose financially from the publication of this manuscript, either now or in the future?
- Do you hold or are you currently applying for any patents relating to the content of the manuscript?
- Have you received reimbursements, fees, funding, or salary from an organization that holds or has applied for patents relating to the content of the manuscript?
- Do you have any other financial competing interests?
- Do you have any non-financial competing interests in relation to this paper?

If you can answer no to all of the above, write 'I declare that I have no competing interests' below. If your reply is yes to any, please give details below.

I declare that I have no competing interests.

I agree to the open peer review policy of the journal. I understand that my name will be included on my report to the authors and, if the manuscript is accepted for publication, my named report including any attachments I upload will be posted on the website along with the authors' responses. I agree for my report to be made available under an Open Access Creative Commons CC-BY license (http://creativecommons.org/licenses/by/4.0/). I understand that any comments which I do not wish to be included in my named report can be included as confidential comments to the editors, which will not be published.

Choose an item.

To further support our reviewers, we have joined with Publons, where you can gain additional credit to further highlight your hard work (see: https://publons.com/journal/530/gigascience). On publication of this paper, your review will be automatically added to Publons, you can then choose whether or not to claim your Publons credit. I understand this statement.

Yes Choose an item.
